# QM/MM study of the reaction mechanism of sulfite oxidase

**DOI:** 10.1038/s41598-018-22751-6

**Published:** 2018-03-16

**Authors:** Octav Caldararu, Milica Feldt, Daniela Cioloboc, Marie-Céline van Severen, Kerstin Starke, Ricardo A. Mata, Ebbe Nordlander, Ulf Ryde

**Affiliations:** 1 Department of Theoretical Chemistry, Lund University, Chemical Centre, P. O. Box 124, SE-221 00 Lund, Sweden; 2 Institut für Physikalische Chemie, Universität Göttingen, Tammannstrasse 6, D-37077 Göttingen, Germany; 3 Chemical Physics, Department of Chemistry, Lund University, Chemical Centre, P. O. Box 124, SE-221 00 Lund, Sweden; 4 Present Address: Division of Quantum Chemistry and Physical Chemistry, Department of Chemistry, Katholieke Universiteit Leuven, Celestijnenlaan 200F, 3001 Leuven, Belgium

## Abstract

Sulfite oxidase is a mononuclear molybdenum enzyme that oxidises sulfite to sulfate in many organisms, including man. Three different reaction mechanisms have been suggested, based on experimental and computational studies. Here, we study all three with combined quantum mechanical (QM) and molecular mechanical (QM/MM) methods, including calculations with large basis sets, very large QM regions (803 atoms) and QM/MM free-energy perturbations. Our results show that the enzyme is set up to follow a mechanism in which the sulfur atom of the sulfite substrate reacts directly with the equatorial oxo ligand of the Mo ion, forming a Mo-bound sulfate product, which dissociates in the second step. The first step is rate limiting, with a barrier of 39–49 kJ/mol. The low barrier is obtained by an intricate hydrogen-bond network around the substrate, which is preserved during the reaction. This network favours the deprotonated substrate and disfavours the other two reaction mechanisms. We have studied the reaction with both an oxidised and a reduced form of the molybdopterin ligand and quantum-refinement calculations indicate that it is in the normal reduced tetrahydro form in this protein.

## Introduction

Molybdenum (Mo) is the only second-row transition metal that is used in biological systems^1^. It is employed in nitrogenases, as well as in a large group of molybdenum oxo-transfer enzymes. The latter is normally divided into three families, depending on the ligands in the active site, viz. the dimethyl sulfoxide reductase (DMSOR), sulfite oxidase (SO) and xanthine oxidase (XO) families (named after a prototypical member of each family)^2,3^. In all three families, Mo is coordinated to a special ligand, the metal-binding pyranopterin ene-1,2-dithiolate, often called molybdopterin (MPT). In most of the enzymes, the Mo ion cycles between the reduced Mo(IV) state and the oxidised Mo(VI) state. In the reduced state, enzymes in the DMSOR family have two MPT ligands and a protein-derived ligand. Enzymes in the SO family have one MPT ligand, an oxo group and a protein-derived ligand, typically cysteine (Cys), whereas those in the XO family have one MPT ligand, one oxo, one sulfido and one hydroxyl ligand^2^. In the oxidised state, all enzymes have an extra oxo group. A comparison of the three families has indicated that the ligands are selected mainly to make the reaction close to thermoneutral, so that the re-reduction or re-oxidation of the active site after the chemical reaction becomes possible^4^.

Sulfite oxidase is an enzyme present in most types of organisms, including man^5,6^. It catalyses the oxidation of sulfite to sulfate in the final step of the degradation of sulfur-containing amino acids:

1 $${{\rm{SO}}}_{3}^{2-}+{{\rm{Mo}}}^{{\rm{VI}}}={\rm{O}}\to {{\rm{SO}}}_{4}^{2-}+{{\rm{Mo}}}^{{\rm{IV}}}$$

The active-site Mo ion is then re-oxidised by cytochrome *c* in two one-electron steps. The electrons are relayed by a heme site in the protein. Deficiency of SO in man may lead to attenuation of brain growth, mental retardation, neurological problems and dislocation of the ocular lens^6^. Crystal structures of SO from several organisms are available^7–11^, showing an active site with Mo bound to MPT, Cys and one (reduced) or two (oxidised) oxo groups. The oxidised structure is approximately square pyramidal with one of the oxo groups in the axial position.

Three different reaction mechanisms have been suggested for SO. Hille suggested that the lone pair of the sulfur atom of ${{\rm{SO}}}_{3}^{2-}$ attacks the equatorial oxo ligand of Mo, directly forming sulfate (S → OMo mechanism)^12,13^. It is supported by the crystal structure of chicken SO, which shows the sulfate product positioned in an ideal way for such a mechanism^7^. On the other hand, Sarkar and coworkers have suggested that sulfite first coordinates to the Mo ion with one of the oxygen atoms, before it reacts with the oxo group (O → Mo mechanism)^14,15^. They showed that a functional inorganic model of SO showed saturation kinetics (in contrast to other oxygen acceptors, e.g. phosphines and phosphites^16^), indicating that an enzyme–substrate complex is formed before the new S–O bond is formed. This mechanism is indirectly supported by EXAFS studies of the complex of SO with arsenate, indicating an As–O–Mo bond^17^. Moreover, EPR studies have indicated that sulfite coordinates to Mo by the oxygen atom^18,19^, but the studies were performed on inhibited Mo(V) forms of a mutant enzyme, indicating that they may represent dead-end species that are not directly involved in the reaction mechanism. Third, it has been suggested that sulfite may instead coordinate to Mo by the sulfur atom, before it reacts (S → Mo mechanism)^20^.

The reaction mechanism of SO has been studied by quantum mechanical (QM) methods by several groups. Thapper et al. examined the S → OMo and S → Mo mechanisms and concluded that the former was more favourable^20^. Kirk and coworkers showed that only the equatorial oxo group is reactive^21^. On the other hand, Sarkar and coworkers provided computational evidence for the O → Mo mechanism, although the other mechanisms were not explicitly tested^22^. Hernandez-Marin and Ziegler tested only the S → OMo mechanism and showed that it is feasible^23^. Recently, we published a thorough QM-cluster study of the reaction mechanism of SO, in which all three mechanisms were compared on equal footing^24^. We studied both protonated and deprotonated substrate (the pK_a_ of ${{\rm{HSO}}}_{3}^{-}$ is 7.2^25^) and compared two models of the MPT ligand. The calculations showed that the S → OMo mechanism gave rise to lower activation barriers than the other two mechanisms. The results strongly depended on the details of the calculations, in particular the QM method, the size of the basis set and the dielectric constant of the continuum solvent.

However, even with a water-like solvent, the barriers were too high to support a biological reaction (139 kJ/mol or higher). Such large barriers were also observed in the other QM studies^20,22,23^ and they are caused by the Coulombic repulsion between the active-site model and the substrate, which both have net negative charges. Hernandez-Marin and Ziegler solved this problem by adding a model of an arginine (Arg) group to the active site, which led to a much smaller barrier of 55 kJ/mol^23^ (a similar model was also used in another study^26^). However, they had to introduce constraints between the Arg model, the substrate and the Mo ion, and our test calculations show that the results very strongly depend on these constraints. Moreover, there are four additional Arg residues, as well as one lysine and one aspartate group in the active site, so it is questionable that only one of these should be included in the calculations.

Therefore, we here perform a combined QM and molecular mechanics (QM/MM) study of the reaction mechanism of SO, in which the surrounding enzyme is included in the calculations. Geometries are obtained with a QM system containing 164 atoms and energies are calculated for a QM region with 805 atoms. Moreover, free energies are obtained by QM/MM thermodynamic cycle perturbations^27,28^, involving molecular dynamics (MD) simulations of the surroundings. The results show that the enzyme is set up to oxidise sulfite via the S → OMo mechanism with a deprotonated substrate.

## Methods

### The protein

The QM/MM calculations were based on the 1.9-Å crystal structure of chicken sulfite oxidase (PDB code 1SOX)^7^. The structure is a homodimer with one Mo active site and a heme group in each subunit. The Fe and Mo ions are 32 Å apart, which has led to the suggestion that the conformation of the protein changes during electron transfer. To avoid speculations of the actual structure of the heme domain, we included in our calculations only all residues with at least one atom within 25 Å of the Mo ion, but filling out to two consecutive sequences, viz. residues 95–460 from the first subunit and residues B344–B441 from the second subunit (residues from the second subunit are marked with a prefix “B”). Six residues had two conformations in the crystal structure and we included the one with the largest occupancy (or the first conformation if both had the same occupancy). Crystal water molecules within 5 Å of the considered residues were included in the calculations, but one glycerol and one sulfate molecule far from the active site were deleted.

The protonation states of all the residues were determined from a study of the hydrogen-bond pattern and the solvent accessibility. It was checked by the PROPKA software^29^. All Arg, Lys, Asp and Glu residues were assumed to be charged. The Cys residue coordinating to Mo was assumed to be deprotonated, whereas the other three Cys residues were protonated. Among the His residues, His140, 177 and 225 were assumed to be protonated on the ND1 atom, His104, 316 and 458 were protonated on NE2 atom, whereas His237 (solvent exposed on the surface of the enzyme) and 283 (forming an ionic pair with the phosphate group of the MPT ligand) were assumed to be doubly protonated.

The protein was protonated and solvated with water molecules forming a sphere with a radius of 30 Å around the Mo ion using the leap module of the Amber software package^30^ (~11 600 atoms in total). The added protons and water molecules were optimised by a 120 ps simulated annealing calculation, followed by a minimisation, keeping the other atoms fixed at the crystal-structure positions. The simulation employed the Amber ff14SB force field^31^ and the TIP3P model for water^32^.

### QM/MM calculations

The QM/MM calculations were performed with the ComQum software^33,34^. In this approach, the protein and solvent are split into three subsystems: System 1 (the QM region) was relaxed by QM methods. System 2 consisted of all residues or water molecules within 6 Å of any atom in system 1 and was relaxed by a full MM minimisation in each step of the QM/MM geometry optimisation in some of the calculations. Finally, system 3 contained the remaining part of the protein and the solvent. It was kept fixed at the original coordinates (equilibrated crystal structure).

In the QM calculations, system 1 was represented by a wavefunction, whereas all the other atoms were represented by an array of partial point charges, one for each atom, taken from MM libraries. Thereby, the polarisation of the QM system by the surroundings is included in a self-consistent manner (electrostatic embedding). When there is a bond between systems 1 and 2 (a junction), the hydrogen link-atom approach was employed: The QM system was capped with hydrogen atoms (hydrogen link atoms, HL), the positions of which are linearly related to the corresponding carbon atoms (carbon link atoms, CL) in the full system^33,35^. All atoms were included in the point-charge model, except the CL atoms^36^. The point charges do not necessarily sum up to an integer, because the Amber force field does not employ charge groups^30^.

The total QM/MM energy in ComQum was calculated as^33,34^

2 $${E}_{{\rm{QM}}/{\rm{MM}}}={E}_{{\rm{QM1}}+{\rm{ptch23}}}^{{\rm{HL}}}+{E}_{{\rm{MM123}},{{\rm{q}}}_{{\rm{1}}}={\rm{0}}}^{{\rm{CL}}}-{E}_{{\rm{MM1}},{{\rm{q}}}_{{\rm{1}}}={\rm{0}}}^{{\rm{HL}}}$$

where ${E}_{{\rm{QM1}}+{\rm{ptch23}}}^{{\rm{HL}}}$ is the QM energy of the QM system truncated by HL atoms and embedded in the set of point charges modelling systems 2 and 3 (but excluding the self-energy of the point charges). ${E}_{{\rm{MM1}},{{\rm{q}}}_{{\rm{1}}}={\rm{0}}}^{{\rm{HL}}}$ is the MM energy of the QM system, still truncated by HL atoms, but without any electrostatic interactions. Finally, ${E}_{{\rm{MM123}},{{\rm{q}}}_{{\rm{1}}}={\rm{0}}}^{{\rm{CL}}}$ is the classical energy of all atoms in the system with CL atoms and with the charges of the QM system set to zero (to avoid double counting of the electrostatic interactions). Thus, ComQum employs a subtractive scheme with electrostatic embedding and van der Waals link-atom corrections (Cao, L. & Ryde, U., Submitted, January, 2018). By this approach, some errors caused by the truncation of the QM system should cancel.

The geometry optimisations were continued until the energy change between two iterations was less than 2.6 J/mol (10^−6^ a.u.) and the maximum norm of the Cartesian gradients was below 10^−3^ a.u. The QM calculations were carried out using Turbomole software^37^. Geometry optimisation were performed using the TPSS^38^ functional in combination with def2-SV(P)^39^ basis set, including empirical dispersion corrections with the DFT-D3 approach^40^, as implemented in Turbomole. The MM calculations were performed with the Amber software^30^, using the Amber ff14SB force field^31^. Transition states are estimated from potential-energy scans.

### QM calculations

All QM calculations were performed with the Turbomole software (versions 6.5 to 7.2)^37^. We employed two density-functional theory (DFT) methods, TPSS^38^ and B3LYP^41–43^, and two different basis sets of different size, def2-SV(P)^39^ and def2-TZVPD^44,45^. The calculations were sped up by expanding the Coulomb interactions in an auxiliary basis set, the resolution-of-identity (RI) approximation^46,47^. Empirical dispersion corrections were included with the DFT-D3 approach^40^, as implemented in Turbomole.

**Figure 1:**
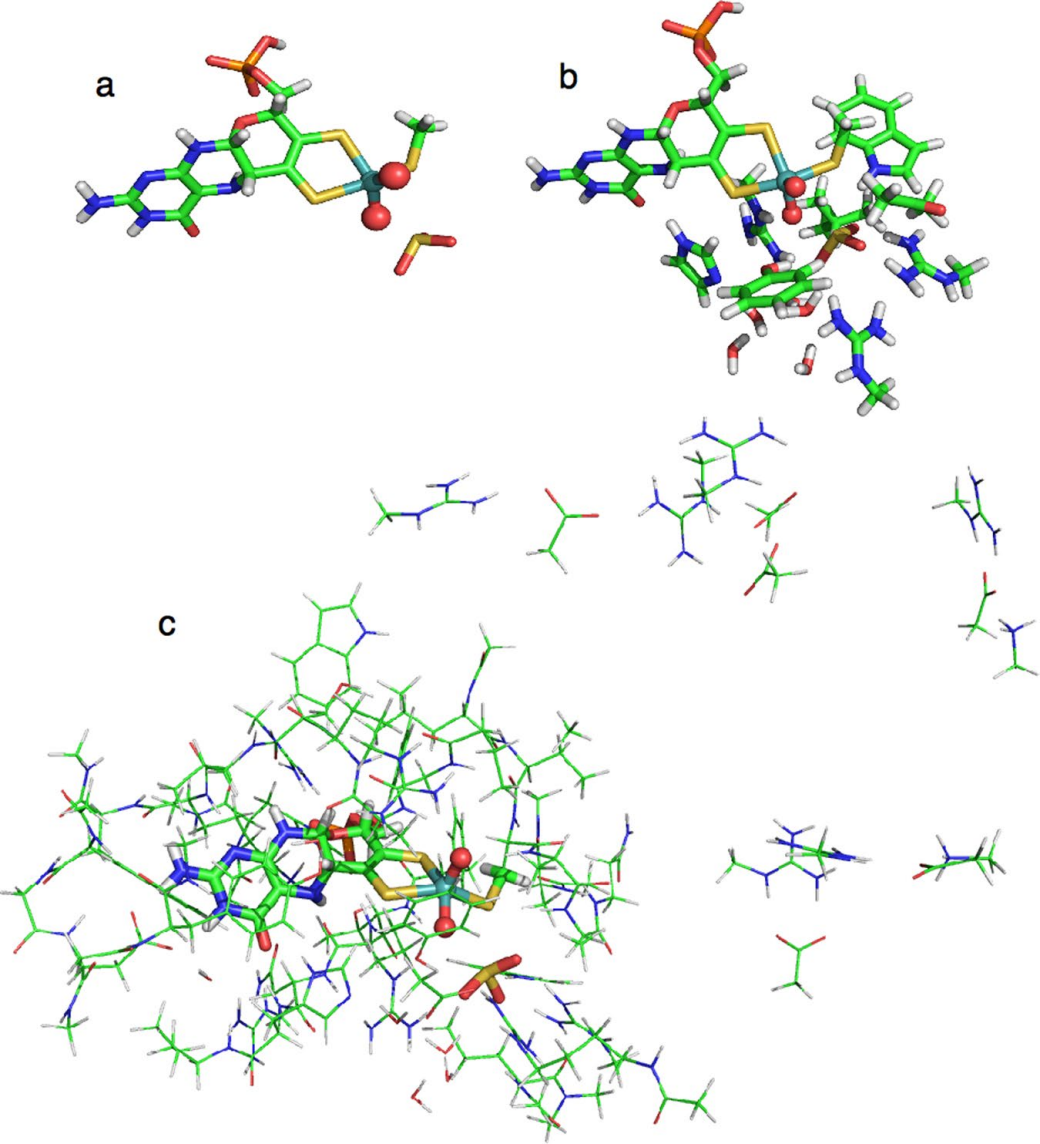
Atoms included in the three QM systems (**a**) small, (**b**) intermediate and (**c**) big-QM system.

QM regions of three different sizes were employed. The small QM region consisted of the Mo ion, the full MPT ligand, CH_3_S^−^ as a model of the Cys91 ligand, two oxo groups and the sulfite substrate. This model consisted of 46 atoms with a net charge of −5 and is shown in Fig. 1a. In the intermediate system, models of nine additional residues were added (CL atom in brackets, following PDB naming conventions): Arg138 (CG), His140 (CB), Ala186 (backbone; CB), Gly187 (C), Arg190 (CG), Leu202 (CA), Trp204 (CB), Tyr322 (CB) and Arg450 (CG), as well as five water molecules. This model consisted of 163 atoms with a net charge of −1 and is shown in Fig. 1b. The third model is the big-QM system, described in the next section.

In the reactant state, Mo is in the +VI oxidation state, but it is converted to the Mo(IV) state during the reaction, concomitantly with the conversion of sulfite to sulfate by the transfer of the equatorial oxo group from Mo to the substrate. Previous calculations have shown that the Mo ion always remains in the closed-shell singlet state^24^ and this was confirmed in the present calculations. For example, the triplet state is ~90 kJ/mol higher than the closed-shell singlet for TS1 and any attempt to find an open-shell singlet always converged to the closed-shell singlet.

### Big-QM calculation

Previous studies have shown that both QM-cluster and QM/MM energies strongly depend on the size of the studied QM system^36,48^. Therefore, we have developed the big-QM approach to obtain converged QM/MM energies^49^: Single-point energy calculation are calculated with a very big QM system, consisting of all chemically reasonable groups (i.e. keeping functional groups, as well as conjugated and aromatic systems intact) with at least one atom within 5.0 Å of any atom in the small QM system defined in the previous section. Moreover, all junctions were moved at least two residues away from the small QM system. In addition, all charged groups buried inside the protein were included, viz. Arg138, Arg189, Arg190, Asp244, Arg260, Asp282, His283, Arg288, Arg298, Lys301, Asp321, Glu344, Arg377, Arg381, Asp383, Lys430 and Asp433 (the latter five residues were included in two copies, one from each protein subunit). This gave a QM system of 803 atoms, shown in Fig. 1c. The big-QM calculations were performed on coordinates from the QM/MM optimisations and with a point-charge model of the surroundings, because this gave the fastest calculations in our previous tests^49^. They also employed the multipole-accelerated resolution-of-identity J approach^50^.

To the big-QM energy, we added a DFT-D3 dispersion correction^41^, calculated for the same big QM system with Becke–Johnson damping^51^, third-order terms and default parameters for the TPSS functional using dftd3 program^52^. We also included the QM/MM MM correction for this large QM system (cf. Eq. 2):

3 $${E}_{{\rm{bigQM}},{\rm{tot}}}^{{\rm{TPSS}}/{\rm{SVP}}}={E}_{{\rm{bigQM}}}^{{\rm{TPSS}}/{\rm{SVP}}}+{E}_{{\rm{disp}}}+{E}_{{\rm{MM12}},{{\rm{q}}}_{{\rm{1}}}={\rm{0}}}^{{\rm{bigQM}}}-{E}_{{\rm{MM1}},{{\rm{q}}}_{{\rm{1}}}={\rm{0}}}^{{\rm{bigQM}}}$$

### QTCP calculations

**Figure 2:**
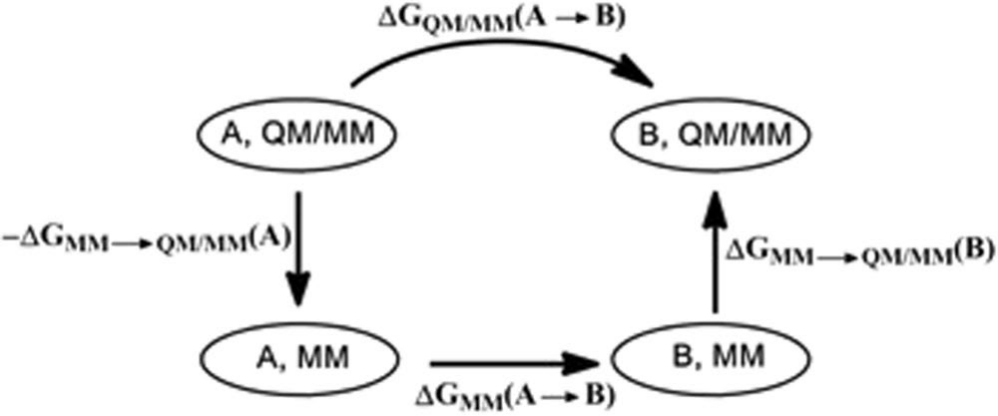
The thermodynamic cycle employed in the QTCP calculations.

The QTCP approach (QM/MM thermodynamic cycle perturbation) is a method to calculate free energies between two states, A and B, with a high-level QM/MM method, using free-energy perturbation (FEP) with sampling at only the MM level^27,28,53^. It employs the thermodynamic cycle shown in Fig. 2, showing that the QM/MM free-energy difference between A and B can be obtained from three calculations: a FEP from A to B at the MM level, a FEP in method space from MM to QM/MM for the A state and a similar calculation for the B state:

4 $${\rm{\Delta }}{G}_{{\rm{QM}}/{\rm{MM}}}({\rm{A}}\to {\rm{B}})={\rm{\Delta }}{G}_{{\rm{MM}}}({\rm{A}}\to {\rm{B}})-{\rm{\Delta }}{G}_{{\rm{MM}}\to {\rm{QM}}/{\rm{MM}}}({\rm{A}})+{\rm{\Delta }}{G}_{{\rm{MM}}\to {\rm{QM}}/{\rm{MM}}}({\rm{B}})$$

The QTCP calculations were performed as has been described before^28,54^: First, each state of interest was optimised by QM/MM, keeping system 2 fixed at the crystal structure. Then, the protein was further solvated in an octahedral box of TIP3P water molecules [75], extending at least 9 Å from the QM/MM system. For the A state, the system was subjected to a 1000-step minimisation, keeping the atoms in the QM part fixed and restraining all heavy atoms from the crystal structure with a force constant of 418 kJ/mol/Å^2^. Then, two 20 ps MD simulations were run with the heavy atoms still restrained. The first was run with a constant volume, the second with a constant pressure. Next, the size of the periodic box was equilibrated by a 100-ps MD simulation with a constant pressure and only the heavy atoms in the QM system restrained to the QM/MM structure. The final structure of this simulation was used as the starting structure also for the other state. Finally, an equilibration of 200 ps and a production of 400 ps were run with a constant volume for each state. During the production run, 200 snapshots were collected every 2 ps.

Based on these snapshots, three sets of FEPs were performed as is shown in Fig. 2. First, FEPs were performed at the MM level in the forward and reverse direction along the reaction coordinate by changing the charges and coordinates of the QM system to those of the QM/MM calculations^54^. Charges were first modified in nine steps, keeping the coordinates at those of the A state. Then, the coordinates were modified in five steps to those of the B state (with the charges in the B state). Finally, MM → QM/MM FEPs were performed for both the A and B states, keeping the QM systems fixed, as has been described before^27^. All FEP calculations were performed with the local software calcqtcp. Further details of the QTCP calculations can be found in http://signe.teokem.lu.se/~ulf/Methods/qtcp.html.

Reported energies are the big-QM energies obtained with TPSS/def2-SV(P) on the QM/MM structures obtained with system 2 relaxed, including dispersion and MM corrections (${E}_{{\rm{bigQM}},{\rm{tot}}}^{{\rm{TPSS}}/{\rm{SVP}}}$ from Eqn. 3). This energy was extrapolated to the B3LYP/def2-TZVPD level of theory using QM calculations of the normal QM system with a point-charge model (${E}_{{\rm{QM1}}+{\rm{ptch23}}}^{{\rm{B3LYP}}/{\rm{TZ}}}-{E}_{{\rm{QM1}}+{\rm{ptch23}}}^{{\rm{TPSS}}/{\rm{SVP}}}$). Finally, the QTCP energy correction (i.e. the difference between the QTCP and QM/MM energies, ${E}_{{\rm{QTCP}}}^{{\rm{TPSS}}/{\rm{SVP}}}-{E}_{{\rm{QM}}/{\rm{MM}}}^{{\rm{TPSS}}/{\rm{SVP}}}$) was also added:

5 $${E}_{{\rm{tot}}}={E}_{{\rm{bigQM}},{\rm{tot}}}^{{\rm{TPSS}}/{\rm{SVP}}}+{E}_{{\rm{QM1}}+{\rm{ptch23}}}^{{\rm{B3LYP}}/{\rm{TZ}}}-{E}_{{\rm{QM1}}+{\rm{ptch23}}}^{{\rm{TPSS}}/{\rm{SVP}}}+{E}_{{\rm{QTCP}}}^{{\rm{TPSS}}/{\rm{SVP}}}-{E}_{{\rm{QM}}/{\rm{MM}}}^{{\rm{TPSS}}/{\rm{SVP}}}$$

### Quantum refinement

Quantum refinement is standard crystallographic refinement supplemented by QM calculations for a small, but interesting part of the protein^55,56^. Crystallographic refinement programs change the protein model (coordinates, occupancies, B factors, etc.) to improve the fit of the observed and calculated structure-factor amplitudes (usually estimated as the residual disagreement, the *R* factor). Owing to the limited resolution normally obtained for biomolecules, the experimental data are supplemented by some chemical information, usually in the form of a MM (or statistics-based) force field^57^. Then, the refinement takes the form of a minimisation or simulated annealing calculation by molecular dynamics using an energy function of the form

6 $${E}_{{\rm{cryst}}}={w}_{{\rm{A}}}{E}_{{\rm{Xray}}}+{E}_{{\rm{MM}}}$$

where *E*_Xray_ is a penalty function that describes how well the model agrees with the experimental data (we have used a maximum-likelihood refinement target using amplitudes, MLF^58,59^). *E*_MM_ is an MM energy function with bond, angle, dihedral and non-bonded terms, and *w*_A_ is a weight factor, which is necessary because *E*_MM_ and *E*_Xray_ do not have the same units. It determines the relative importance of the crystallographic raw data and the MM force field for the final structure.

Quantum chemistry can be introduced in this function by replacing the MM potential for a small region of the protein (system 1) by a quantum mechanics (QM) calculation (in analogy to the QM/MM calculations), yielding a QM energy for system 1, *E*_QM1_^55^. To avoid double counting we must then subtract the MM energy of system 1, *E*_MM1_,

7 $${E}_{{\rm{cqx}}}={w}_{{\rm{A}}}{E}_{{\rm{Xray}}}+{E}_{{\rm{MM12}}}+{w}_{{\rm{QM}}}{E}_{{\rm{QM1}}}-{E}_{{\rm{MM1}}}$$

Thereby, we introduce an accurate energy function for the system of interest. Such an energy function is implemented in the software ComQum-X^55^, which is a combination of the software Turbomole^38^ and the crystallography and NMR system (CNS)^60,61^, version 1.3. The factor *w*_QM_ in Eq. 7 is another weight, which is needed because the CNS MM force field is based on a statistical analysis of crystal structures^62^. Therefore, the force constants are not energy-derived, as is the QM term, but they are in arbitrary statistical units. Experience has shown that the CNS force constants are typically three times larger than energy-based force constants^62^, and *w*_QM_ = 3 has therefore been used throughout this work^55^. Crystallographic refinement is traditionally performed without any electrostatic interactions, because hydrogen atoms are not discerned in the structure. We followed this custom and excluded electrostatics and hydrogen atoms from all crystallography and MM calculations (but they are of course included in the QM calculations). In analogy with the QM/MM calculations, the QM system was truncated by H atoms.

The quantum-refinement calculations were based on another 2.0-Å crystal structure of chicken sulfite oxidase^10^ (because structure factors were not available in the protein databank for the 1SOX structure). It involves the same product-inhibited complex as the 1SOX structure, with a water molecule coordinating to the Mo ion and a second-sphere sulfate molecule, but it was based on a synthesised gene. Coordinates, occupancies, B factors, and structure factors were obtained from the Protein Data Bank files 2A9A. From these files, we also obtained the space group, unit-cell parameters, resolution limits, *R* factors and the test set used for the evaluation of the *R*_free_ factor.

The full protein was used in all calculations, including all crystal water molecules. In each cycle of the geometry optimisation, the surrounding protein was allowed to relax by one cycle of crystallographic minimisation and one cycle of individual *B*-factor refinement. However, the new coordinates and *B* factors were accepted only if the *R* factor was reduced. For the protein, we used the standard CNS force field (protein_rep.param, water_rep.param, and ion.param). The MM force field for non-standard residues was downloaded from the hetero-compound information centre in Uppsala^63^. The *w*_A_ factor was determined by CNS to 1.06. Electron density maps were generated using *phenix*.*maps*^64^.

The quality of the models was compared using the real-space difference density Z-score^65^ (RSZD), calculated by EDSTATS, which measures the local accuracy of the model. The maximum of the absolute values of the positive and negative RSZD (combined RSZD) for MPT, Mo and ${{\rm{SO}}}_{4}^{2-}$was taken as the quality metric.

## Result and Discussion

**Figure 3:**
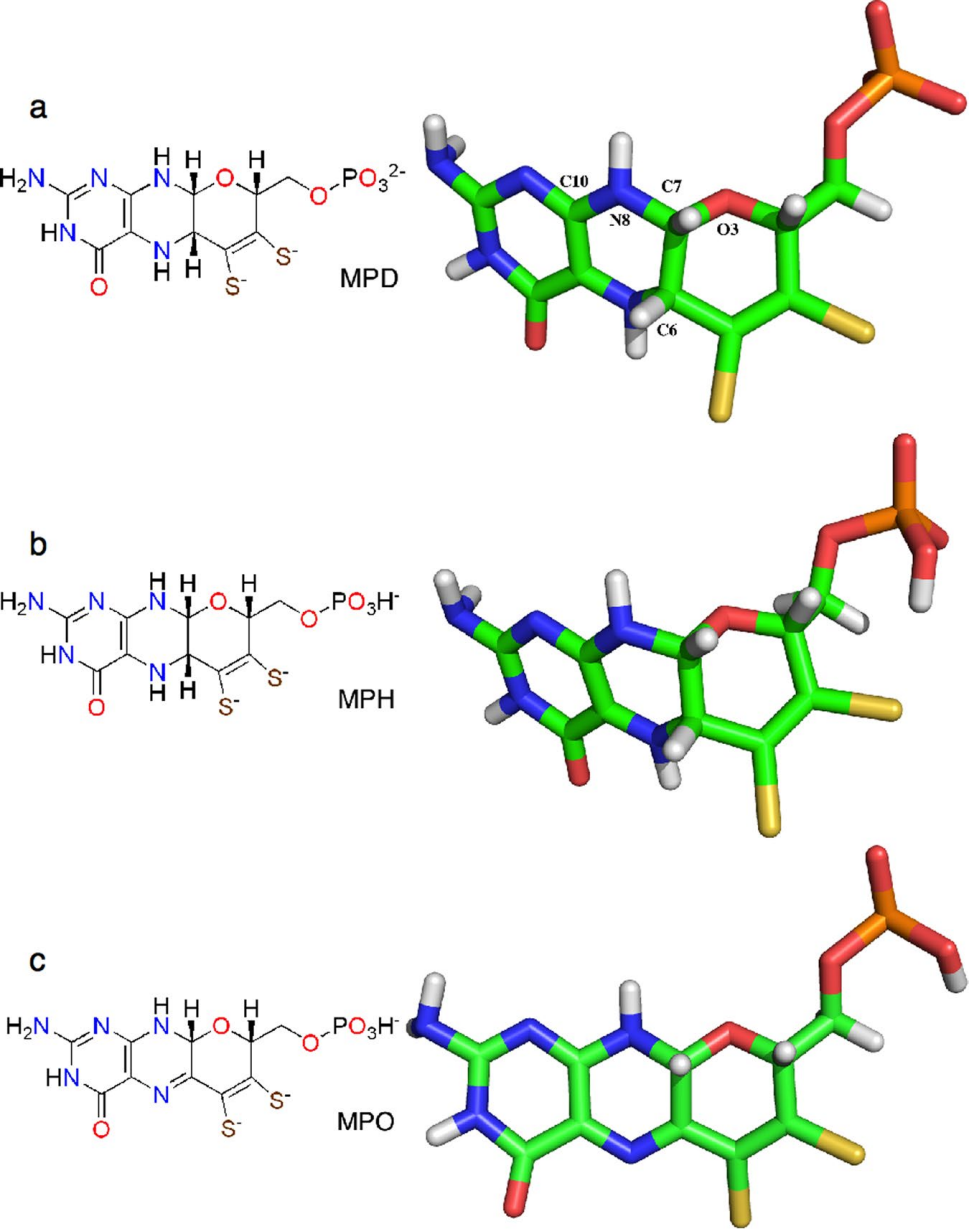
The three different MPT models employed in this study: (**a**) the reduced state with a deprotonated phosphate group (MPD), (**b**) the reduced state with a protonated phosphate group (MPH) and (**c**) the oxidised dihydro state with a protonated phosphate group (MPO).

We have studied the reaction mechanism of sulfite oxidase with QM/MM methods, based on the crystal structure of the enzyme from chicken^7^. We employed QM regions of three different sizes, as is shown in Fig. 1a–c. All involved the entire MPT ligand. A problem with this ligand is that it can assume several states. For example, the phosphate group can be either fully deprotonated (Fig. 3a) or singly protonated on the phosphate group (Fig. 3b; the second p*K*_a_ value of phosphate is 6.6^25^). Examination of the crystal structure shows that two of the phosphate oxygen atoms form strong hydrogen bonds with the positively charged sidechains of Lys301, Arg288 and His283 (1.69–1.78 Å), and also a weaker hydrogen bond to the backbone N atom of Arg138 (2.14 Å). However, the third oxygen atom (O3P) does not receive any hydrogen bond. Instead, it is close to the backbone O atom of Phe136 (2.55 Å O–O distance). Therefore, it is likely that this oxygen atom is protonated, although we performed calculations with both protonation states.

Moreover, it is normally assumed that the MPT group is in the reduced tetrahydro state, shown in Fig. 3a,b (with a deprotonated, MPD, or protonated phosphate group, MPH). However, it has been suggested (based on the conformation of this group in crystal structures) that in sulfite oxidase, this group is instead in the oxidised 10,10a-dihydro state, shown in Fig. 3c^66^. This changes the N5 atom from a hydrogen-bond donor to an acceptor, but it neither receives nor accepts any hydrogen bonds in the crystal structure, making the decision ambiguous. Again, we tried both states in our calculations (with a protonated phosphate group in the dihydro state, MPO).

We will start by describing the most likely mechanism in detail. Then we discuss our attempts to study the other two reaction mechanisms and different protonation states of the substrate. Finally, we compare results obtained with the three different models of the MPT ligand.

### S → OMo mechanism

We started from a crystal structure of a product-inhibited complex^7^. It contains a water molecule coordinated to the Mo ion (in addition to the MPT ligand and Cys185) and a sulfate molecule bound close to the Mo ion (Mo–S distance 5.2 Å and Mo–O distances of 4.7–6.7 Å). The four sulfate O atoms form strong hydrogen bonds with the surrounding protein, viz. to Arg190 (2.65–2.75 Å; there are two active sites in the homodimeric crystal) and Arg 450 (2.66–3.23 Å), to Arg190 (3.15–3.19 Å) and Trp204 (2.72–2.81 Å), to Arg190 (2.95–3.20 Å), Leu202 (backbone, 3.26–3.37 Å), Arg450 (3.09–3.18 Å) and a water molecule (2.01–2.48 Å) and to Arg138 (2.52–2.63 Å), Tyr322 (2.92–2.93 Å) and the Mo-bound water molecule (2.42–2.44 Å).

**Figure 4:**
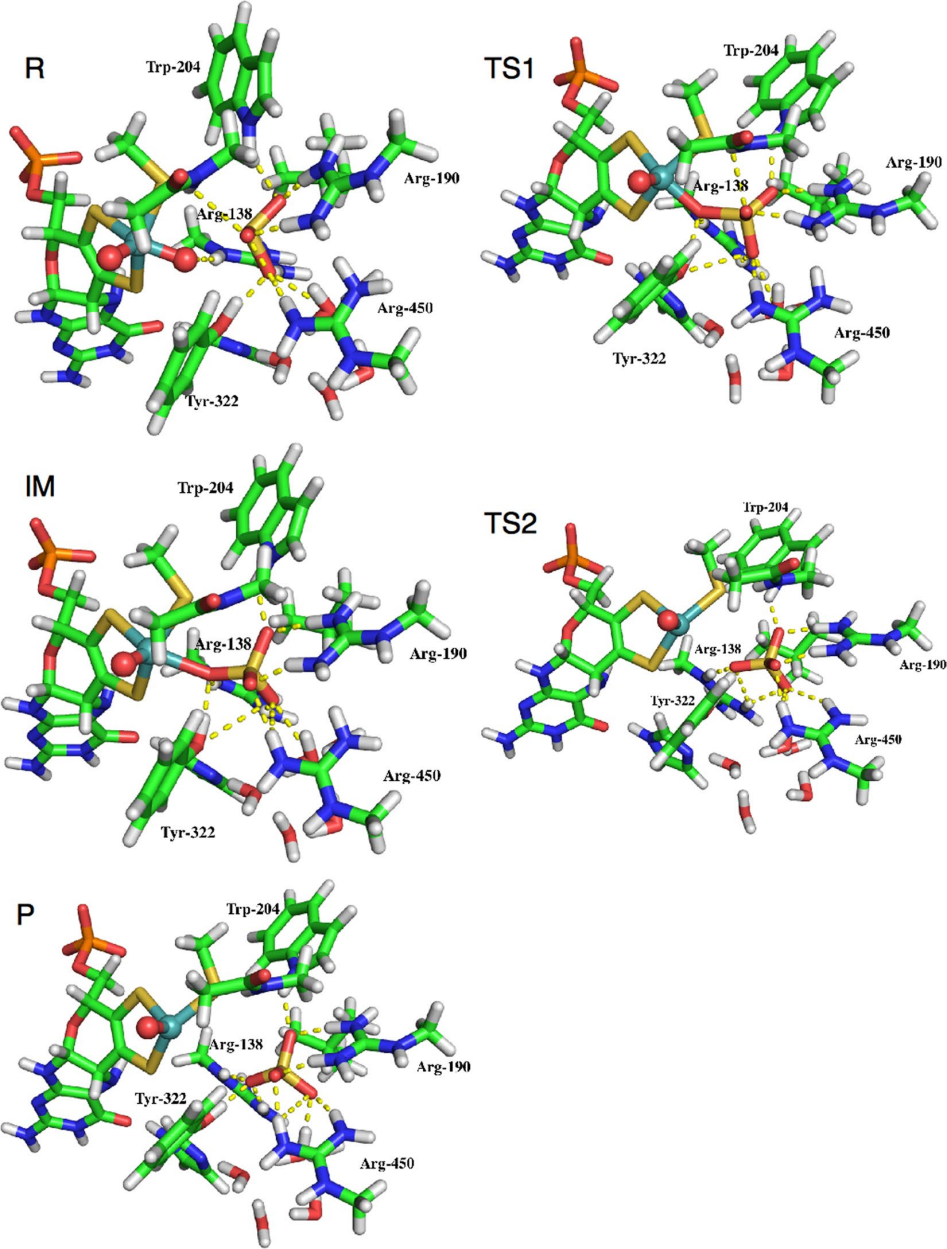
Structures of the five states in the reaction. Hydrogen bonds to the O atoms are indicated in yellow broken lines. Mo and oxo groups are spheres in cyan and red, respectively.

We could obtain a proper reaction path for the S → OMo mechanism by removing the Mo-bound water molecule in the crystal structure and then driving the O atom of the sulfate product that is closest to Mo (the one that forms hydrogen bonds to Arg138, Tyr322 and the Mo-bound water molecule) towards the Mo ion. A product state (P) was obtained with a Mo–O distance of 4.08 Å (with the MPD model), i.e. somewhat closer than in the crystal structure, owing to the deletion of the water molecule. It still has an approximately square pyramidal geometry with one empty equatorial coordination site, as can be seen in Fig. 4. An intermediate (IM) could be obtained with the sulfate product coordinating to Mo with a Mo–O distance of 2.34 Å. A transition state (TS2) was obtained at a Mo–O distance of 3.30 Å, i.e. approximately midway between the two states.

From the intermediate, we tried to cleave the O–S bond, which is 1.58 Å in IM (and 1.53 Å in P). This could quite easily be obtained via a transition state (TS1) with an O–S bond length of 1.90 Å. When fully cleaved, the O–S distance is 2.58 Å, representing the reactant (R) state with a sulfite substrate. The latter molecule has moved back to a position close to that of the sulfate product, with a Mo–S distance of 4.00 Å and Mo–O distances of 4.42–5.22 Å.

The Mo–O_ax_ and Mo–S_Cys_ bond lengths vary only minimally during the reaction, 1.74–1.76 Å and 2.42–2.44 Å, as can be seen in Table 1. For the two Mo–S_MPT_ bonds, the variation is larger, 2.33–2.49 Å (S1 and S2 in Table 1). The shortest bonds are trans to the empty coordination site in the P and TS2 states and the longest bond is trans to O_eq_ in the R state. Besides these, the bonds are 2.42–2.45 Å for R and TS1 and 2.37–2.40 Å for the other states, reflecting the reduction of Mo.

**Table 1:** Mo–ligand and S_Sub_–O distances in the various structures obtained in this study.

MPT	State	Distance to Mo (Å)					Distance to S_Sub_ (Å)			
		S_Cys_	S1	S2	O_ax_	O_eq_	O1	O2	O3	O_eq_
MPD	R	2.44	2.49	2.45	1.76	1.76	1.56	1.56	1.64	2.58
	TS1	2.43	2.43	2.42	1.75	2.05	1.52	1.52	1.54	1.90
	IM	2.43	2.39	2.40	1.74	2.34	1.53	1.51	1.52	1.58
	TS2	2.42	2.34	2.37	1.74	3.30	1.54	1.51	1.53	1.54
	P	2.42	2.33	2.37	1.75	4.08	1.54	1.50	1.54	1.53
MPH	R	2.42	2.50	2.45	1.76	1.76	1.55	1.56	1.63	2.54
	TS1	2.41	2.45	2.45	1.74	1.96	1.53	1.54	1.55	2.00
	IM	2.42	2.39	2.42	1.74	2.29	1.51	1.51	1.53	1.60
	TS2	2.40	2.33	2.38	1.74	3.70	1.51	1.51	1.55	1.54
	P	2.40	2.33	2.38	1.74	3.85	1.54	1.51	1.54	1.54
MPO	R	2.42	2.51	2.46	1.76	1.76	1.55	1.56	1.63	2.52
	TS1	2.41	2.46	2.45	1.74	1.96	1.53	1.53	1.55	2.00
	IM	2.41	2.39	2.42	1.74	2.28	1.51	1.51	1.53	1.60
	P	2.40	2.33	2.38	1.74	3.85	1.54	1.51	1.54	1.54

S_Sub_, O1, O2 and O3 are the four atoms of the substrate. S_Cys_ is the S atom of Cys185. S1 and S2 are the two S atoms of MPT. O_ax_ and O_eq_ are the two oxo ligands of Mo.

The full reaction can be performed with only small movements of all atoms, as can be seen in Fig. 4; the largest movement for any atom during the reaction is 2.2 Å, viz. for the equatorial oxo group which becomes the fourth O atom of sulfate. One of the sulfite O atoms also moves by 1.8 Å (the one that takes the position most distant from Mo in the sulfate state). The sulfite S atom moves by only 0.8 Å during the reaction and the other two O atoms of sulfite move by 0.6–0.8 Å, whereas the Mo ion is essentially fixed (movement less than 0.2 Å).

The limited movement permits the substrate molecule to retain favourable hydrogen bonds to the surrounding protein throughout the reaction, as can be seen in Table 2. Six hydrogen bonds are observed in all five reaction states, viz. to Arg190 (1.59–1.78 Å to O1 and 1.57–1.83 Å to O2), Trp204 (1.94–2.12 Å to O2), Arg138 (1.97–2.47 Å to O_eq_), Tyr322 (1.59–2.40 Å to O_eq_) and a water molecule (1.68–1.90 Å to O3). Arg450 forms a hydrogen bond to O3 for the R and TS1 states with one of the H atoms (1.86–1.87 Å) and with another atom to the TS2 and P states (1.89–2.33 Å). Tyr322 forms an additional hydrogen bond to O3 for the R and TS1 states (1.66–2.50 Å), whereas Arg138 forms an additional hydrogen bond to O3 in the all states except P (1.82–2.20 Å) and to O_eq_ for the TS2 and P states (1.91–2.45 Å). Arg450 forms a hydrogen bond also to O1 for the last three states (1.78–1.98 Å). Consequently, in all five states, the substrate molecule (including O_eq_) forms 8–10 hydrogen bonds (with H–O distances <2.5 Å) to the protein and one water molecule throughout the reaction, an ingenious setup to keep all states close to each other in energy terms.

**Table 2:** Hydrogen bonds in the various structures (Å).

Acc	Donor	MPD					MPH					MPO			
		R	TS1	IM	TS2	P	R	TS1	IM	TS2	P	R	TS1	IM	P
O1	HH22 Arg190	1.59	1.76	1.75	1.78	1.78	1.63	1.77	1.81	1.85	1.81	1.63	1.77	1.81	1.81
	HH22 Arg450			1.98	1.81	1.78					1.81				1.81
O2	HH12 Arg190	1.57	1.83	1.81	1.74	1.76	1.62	1.78	1.94	1.77	1.77	1.62	1.78	1.93	1.77
	HE1 Trp204	2.12	1.98	1.95	1.94	1.98	2.02	1.93	1.97	1.91	1.92	2.01	1.93	1.97	1.92
O3	HH21 Arg138	1.89	2.10	1.82	2.20		1.91	2.02	2.07			1.92	2.05	2.11	
	HH Tyr322	1.66	2.50				1.68	2.46	2.45			1.68	2.47	2.45	
	HH12 Arg450				2.33	1.89				1.97	2.01				2.00
	HH22 Arg450	1.87	1.86				1.95	1.92	1.88	1.91		1.93	1.91	1.87	
	H1 Wat	1.80	1.90	1.83	1.69	1.68	1.84	1.92	1.92	1.68	1.68	1.85	1.93	1.91	1.69
O_eq_	HE Arg138	2.47	2.17	2.31	1.97	2.04	2.38	2.17	2.16	1.89	1.95	2.41	2.18	2.19	1.98
	HH21 Arg138				2.45	1.91				2.18	2.05				2.04
	HH Tyr322	2.40	1.59	1.69	1.63	1.65	2.39	1.63	1.71	1.63	1.64	2.38	1.62	1.71	1.64

Acc is the acceptor atom, viz. the three oxygen atoms of ${{\rm{SO}}}_{3}^{2-}$ and the equatorial oxo group. Only hydrogen bonds shorter than 2.5 Å are shown.

### Reaction energies

**Figure 5:**
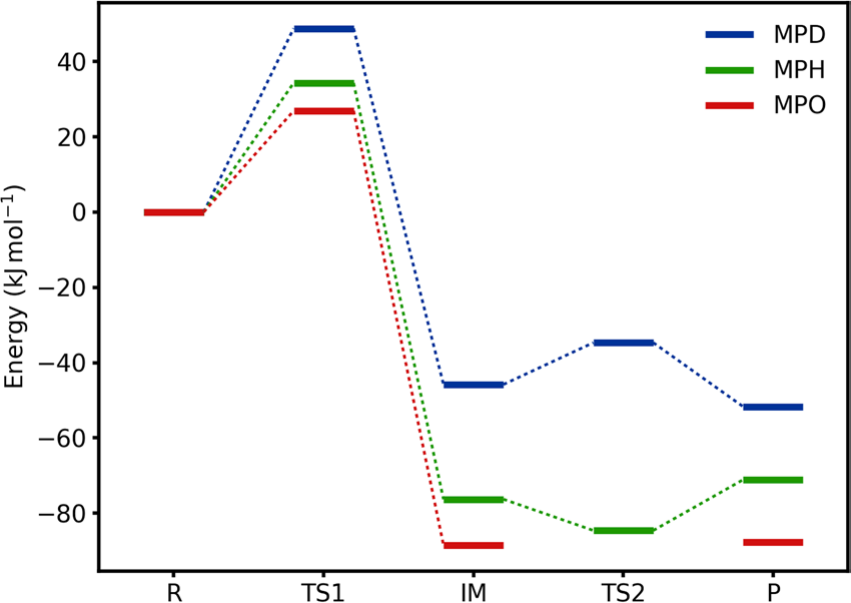
Reaction energies for the three MPT models.

Next, we consider the energies of the S → OMo reaction mechanism in the enzyme. The energies are shown in Fig. 5 (blue line for MPD). It can be seen that the highest energy barrier is observed for the first transition state (TS1), 49 kJ/mol. This transition state represents the chemical step when the O_eq_–S_Sub_ bond is formed and Mo is reduced to Mo^IV^. The intermediate (IM) is appreciably more stable than the reactant state, 46 kJ/mol. The second transition state, which represents the dissociation of the sulfate product from Mo, is only slightly uphill compared to the intermediate, 11 kJ/mol, which is appreciably less than the first barrier. Therefore, the first step is rate limiting.

The product is also slightly more stable than the intermediate, 6 kJ/mol. This may seem somewhat small, considering that the intermediate is not observed and that the crystal structure involves the sulfate product, dissociated from Mo, but still binding to the protein. However, in this structure, there is a water molecule coordinating to Mo, which is not present in our model of the product state. Thus, the dissociation of the product may be facilitated by this water molecule. We have not attempted to study such a reaction because the dissociation barrier is small and binding reactions are problematic to model in QM calculations, because the binding path of the water molecule would be speculative (where does it bind before coordinating to Mo?) and binding energies are less accurate than reaction energies, with large entropy contributions.

The total energies in Fig. 5 involve several components (cf. Eq. 5). They are based on the big-QM energies, calculated at the TPSS/def2-SV(P) level. To these, a dispersion energy estimate has been added, calculated by the DFT-D3 approach^40^ with Becke–Johnson damping^51^ and including third-order terms (Eq. 3). From the energy components in Table 3, it can be seen that this correction (*E*_disp_) is small for the relative energies, up to 4 kJ/mol, reflecting that the substrate, intermediate and product are all bound to the enzyme in a similar way. Likewise, the MM energy correction for the relative big-QM energies (*E*_MM_) is also small, up to 5 kJ/mol, reflecting the use of a very large QM system.

**Table 3:** Energy components (kJ/mol).

MPT		${{\boldsymbol{E}}}_{{\bf{QM}}/{\bf{MM}}}^{{\bf{TPSS}}/{\bf{SVP}}}$	*E*_DFT_	${{\boldsymbol{E}}}_{{\bf{bigQM}}}^{{\bf{TPSS}}/{\bf{SVP}}}$	*E*_disp_	*E*_MM_	${{\boldsymbol{E}}}_{{\bf{bigQM}},{\bf{tot}}}^{{\bf{TPSS}}/{\bf{SVP}}}$	${{\boldsymbol{E}}}_{{\bf{QTCP}}}^{{\bf{TPSS}}/{\bf{SVP}}}$	*E*_tot_
MPD	R	0.0	0.0	0.0	0.0	0.0	0.0	0.0	0.0
	TS1	56.8	−26.1	60.9	−1.8	−4.5	54.6	77.0	48.8
	IM	25.0	−82.8	21.2	−2.4	−3.0	15.8	46.3	−45.8
	TS2	49.3	−97.7	50.3	2.8	2.3	55.4	56.9	−34.7
	P	45.4	−96.6	41.9	3.9	0.8	46.6	43.7	−51.7
MPH	R	0.0	0.0	0.0	0.0	0.0	0.0	0.0	0.0
	TS1	39.3	−8.9	43.6	−2.0	−5.2	36.4	46.1	34.3
	IM	−3.6	−79.6	0.8	0.3	−4.2	−3.1	2.8	−76.3
	TS2	52.5	−98.8	51.4	2.0	−2.2	51.3	15.4	−84.6
	P	47.1	−97.0	41.0	4.2	0.4	45.6	27.5	−71.1
MPO	R	0.0	0.0	0.0	0.0	0.0	0.0	0.0	0.0
	TS1	35.6	−10.0	36.6	−3.0	−4.0	29.6	42.9	26.9
	IM	−9.6	−79.0	−7.4	−2.2	−3.1	−12.7	−6.4	−85.5
	P	42.1	−96.0	35.3	−2.2	0.5	33.5	16.9	−87.7

The energy components are defined in Eqns 3 and 5, except that ${{\boldsymbol{E}}}_{{\rm{M}}{\rm{M}}}={{\boldsymbol{E}}}_{{\rm{M}}{\rm{M}}12,{{\rm{q}}}_{1}=0}^{{\rm{b}}{\rm{i}}{\rm{g}}{\rm{Q}}{\rm{M}}}-{{\boldsymbol{E}}}_{{\rm{M}}{\rm{M}}1,{{\rm{q}}}_{1}=0}^{{\rm{b}}{\rm{i}}{\rm{g}}{\rm{Q}}{\rm{M}}}$ and ${{\boldsymbol{E}}}_{{\rm{D}}{\rm{F}}{\rm{T}}}={{\boldsymbol{E}}}_{{\rm{Q}}{\rm{M}}1+{\rm{p}}{\rm{t}}{\rm{c}}{\rm{h}}23}^{{\rm{B}}3{\rm{L}}{\rm{Y}}{\rm{P}}/{\rm{T}}{\rm{Z}}}-{{\boldsymbol{E}}}_{{\rm{Q}}{\rm{M}}1+{\rm{p}}{\rm{t}}{\rm{c}}{\rm{h}}23}^{{\rm{T}}{\rm{P}}{\rm{S}}{\rm{S}}/{\rm{S}}{\rm{V}}{\rm{P}}}$ (the latter two terms are not explicitly shown, because ${{\boldsymbol{E}}}_{{\rm{QM1}}+{\rm{ptch23}}}^{{\rm{TPSS}}/{\rm{SVP}}}$ agrees with ${{\boldsymbol{E}}}_{{\rm{QM}}/{\rm{MM}}}^{{\rm{TPSS}}/{\rm{SVP}}}$ within 9 kJ/mol (showing that the MM correction is small for the intermediate QM system). Thus, ${{\boldsymbol{E}}}_{{\rm{tot}}}$ = ${{\boldsymbol{E}}}_{{\rm{bigQM}},{\rm{tot}}}^{{\rm{TPSS}}/{\rm{SVP}}}+{{\boldsymbol{E}}}_{{\rm{DFT}}}+{{\boldsymbol{E}}}_{{\rm{QTCP}}}^{{\rm{TPSS}}/{\rm{SVP}}}-{{\boldsymbol{E}}}_{{\rm{QM}}/{\rm{MM}}}^{{\rm{TPSS}}/{\rm{SVP}}}$  = ${{\boldsymbol{E}}}_{{\rm{bigQM}}}^{{\rm{TPSS}}/{\rm{SVP}}}+{{\boldsymbol{E}}}_{{\rm{disp}}}+$ ${{\boldsymbol{E}}}_{{\rm{MM12}},{{\rm{q}}}_{{\rm{1}}}={\rm{0}}}^{{\rm{bigQM}}}-{{\boldsymbol{E}}}_{{\rm{MM1}},{{\rm{q}}}_{{\rm{1}}}={\rm{0}}}^{{\rm{bigQM}}}$ $+{{\boldsymbol{E}}}_{{\rm{DFT}}}+{{\boldsymbol{E}}}_{{\rm{QTCP}}}^{{\rm{TPSS}}/{\rm{SVP}}}-{{\boldsymbol{E}}}_{{\rm{QM}}/{\rm{MM}}}^{{\rm{TPSS}}/{\rm{SVP}}}$.

Interestingly, the QM/MM and (total) big-QM energies are very similar with a maximum difference of only 9 kJ/mol. This shows that the intermediate QM system, employed for the QM calculations is properly chosen, i.e. that it is large enough to give energies that are reliable. Such a QM system of intermediate size is not always easy to select^36,48^. It also indicates that the QTCP energy correction is reliable (because it is calculated for the intermediate QM system).

To this corrected big-QM energy, we have added a correction for the change of the QM method and basis set to the more accurate B3LYP functional with the larger def2-TZVPD basis set (*E*_DFT_), which has been shown to give reliable results for the sulfite-oxidase reaction^24^. From Table 3, it can be seen that this correction is very important, changing the energies by up to 98 kJ/mol, strongly stabilising the IM, TS2 and P states.

Finally, a QTCP correction is added, which includes the entropy of the protein, as well as the effect of relaxing the surroundings. For the PS and TS2 states, it is rather small (up to 8 kJ/mol), but for the TS1 and IM states, it is larger, 20–21 kJ/mol, increasing the energy of both states relative to R.

### Attempts to obtain other reaction mechanisms and protonation states

We have spent much effort to obtain also the other two reaction mechanisms (O → Mo and S → Mo) in the enzyme. However, these attempts were unsuccessful. The crucial step of both mechanisms is to find an intermediate with sulfite coordinated to Mo, either with the S atom or with one of the three O atoms. This requires an increase in the coordination number of Mo from five to six and thereby a significant change in the geometry of the metal site. Moreover, at least in the O → Mo mechanism, the binding of one of the sulfite O atoms to Mo will partly hide this atom and make it less accessible for hydrogen bonding, which may require major changes in the hydrogen-bond pattern. We have systematically deleted one of the four O atoms of the sulfate product in the starting crystal structure and then decreased one of the Mo–O distances or the Mo–S distance. However, all attempts have led to uphill reactions without any stable intermediates. Some examples are given in the supplementary material, Figs S1 and S2. Calculations were performed with the surrounding protein fixed at the crystal structure or relaxed by MM. In addition, we tried to run MD simulations for states with constrained Mo–O or Mo–S distances to further relax the surroundings for the structures of the putative intermediates, but with no success.

Likewise, we have also systematically studied the reaction mechanisms with a protonated substrate (${{\rm{H}}{\rm{S}}{\rm{O}}}_{3}^{-}$). In this case, the problem is to find a protonation site that does not interfere with the hydrogen-bonding network around the substrate. As described above, this network involves mainly hydrogen-bond donors; only Tyr322 and the water molecule may be hydrogen-bond acceptors. However, Arg138 and 450 form hydrogen bonds to the same O atom as the water molecule and in several states, the H atoms are quite close, so it is hard to imagine that the water molecule could be an acceptor.

On the other hand, the axial oxo group may accept a hydrogen bond from the substrate, as was observed in our previous QM-cluster calculations. This group is hydrogen-bonded to the backbone NH groups of Ala186 and Ala297 (1.9–2.2 Å), but there is room for a third hydrogen bond in the direction of the substrate. However, the shortest O_ax_–O_Sub_ distance is too long for a favourable H bond even in the IM state (4.5 Å). Consequently, in the R state with ${{\rm{H}}{\rm{S}}{\rm{O}}}_{3}^{-}$, the proton on the substrate instead interacts with O_eq_, thereby placing the substrate in a poor geometry for the reaction, with a S–O_eq_ distance of 3.16 Å. Still, an IM state could be found, but it was 108 kJ/mol less stable than for the deprotonated substrate, thereby making such a mechanism unlikely.

Another possibility is that the proton is directed towards Leu202, where there is some empty space. Again, a stable R state could be found with a more reasonable S–O_eq_ distance (2.60 Å). However, the proton did not form any favourable interaction with the protein. Instead, it formed a short H–H interaction with HG of Leu202 (1.68 Å). The IM state was also found, still giving a short H–H distance, this time to one of the HD1 atoms of Leu202 (1.60 Å). Consequently, it was 97 kJ/mol higher than for the deprotonated substrate.

However, if the substrate was protonated on the atom directed towards Tyr322, a stable R state could be found, with the proton forming a very short hydrogen bond to OH of Tyr322, 1.48 Å. Interestingly, in this state, the HH atom (the hydroxyl proton) of Tyr322 has moved to His140, so that Tyr322 formally became negatively charged. The IM state could also be found, but now the proton had moved from sulfate to Tyr322, so that the latter is neutral again (and His140 is still positively charged). It was 4 kJ/mol more stable than the corresponding state with a deprotonated substrate. However, no product state could be found with the protonated substrate: With the Mo–O_eq_ bond constrained to 3.85 Å, this state was 10 kJ/mol less stable than the corresponding state with a deprotonated substrate, but when the constraint was removed, the structure returned to the IM state. This shows that a protonated substrate is unlikely to be the true substrate of the enzyme. Moreover, the calculations also show that the energies are rather insensitive to the protonation state of His140.

### Differences between different MPT models

The results discussed so far are based on calculations with the deprotonated and reduced MPD model. We also performed calculations with the protonated MPH model and with the oxidised (and protonated) MPO model (results in Tables 1, 2 and 3 and Fig. 5). The MPH and MPO models gave very similar structures, with differences in the key distances to Mo or S_Sub_ in Table 1 of less than 0.02 Å and less than 0.04 Å for the hydrogen-bond distances in Table 2. The only significant difference was that the TS2 state could not be found with the MPO model; instead, the energy decreased monotonically when decreasing the Mo–O_eq_ distance starting from P, although with an extremely flat potential (within 2 kJ/mol) between Mo–O_eq_ = 3.9–3.3 Å. Still, the energies in Fig. 5 agree within 17 kJ/mol.

However, the difference in structures obtained with these two models were somewhat larger when compared to the deprotonated MPD. In particular, the Mo–O_eq_ distance was significantly longer in TS2 (3.70 Å) and shorter in P (3.85 Å). The S_Sub_–O_eq_ bond was also slightly longer in TS1 (2.00 Å). This is also reflected in some variation of the hydrogen bonds to Arg138, Tyr322 and Arg450 in the IM and TS2 states, and in the hydrogen-bond distances for the other states of up to 0.15 Å. In fact, for the MPH structures, all five states have nine hydrogen bonds throughout the reaction (Table 2). These differences in the structures and hydrogen-bond pattern lead to rather large differences also in the energies, up to 50 kJ/mol. They come mainly from the QTCP energy, although the QM/MM and big-QM energies may differ by up to 29 and 19 kJ/mol, respectively as can be seen in Table 3.

From Fig. 5, it can be seen that MPO gives the lowest activation barrier (27 kJ/mol) and the most exothermic reaction (−88 kJ/mol). However, MPH gives a similar barrier (34 kJ/mol) and also that of MPD (49 kJ/mol, corresponding to a rate of ~2 · 10^4^ s^−1^) is so low that this step should not be rate limiting in the enzyme. Therefore, it is not possible to determine which state of MPT is used in the enzyme from the reaction energies, especially considering that the Mo oxygen-atom transfer enzymes do not seem to have evolved to give exothermic reaction energies^4^.

A conspicuous feature of the reduced cofactors (MPD and MPH) is that the H5 atom in the middle ring is bent distinctly out of the ring plane (the C10–C9–N5–H5 torsion is around −100°), whereas it is within the plane in QM-cluster structures (~0°)^24^. This may be caused by the formation of a weak hydrogen bond to the backbone O atom of Arg138 and steric repulsion to the HD atoms of the same residue. In fact, we could enforce H5 to remain in the ring plane, but such a structure was 19 kJ/mol less stable than the non-planar one. However, even for the isolated QM system in vacuum, the non-planar conformation is 1 kJ/mol more stable than the planar conformation.

We have studied the α (C10–N8–C7–C6) and β (C10–N8–C7–O3′) torsion angles of the MPT molecule, discussed in ref.^66^, in the QM/MM structures, employing the three different models. The atoms in the torsions are shown in Fig. 3a and the calculated angles are listed in Table 4. The angles with the MPH model (−22 and 98°, average over the five states) are closer to those in the starting crystal structure (−18 and 95°, averages over the two subunits) than the other two models. On the other hand, those in the MPD model (−32 and 87°) are closer to the ideal value of a free MPD molecule (−48 and 76°), than the other two models. However, it should be noted that these two dihedrals are quite floppy modes; in fact, they can be varied by over 80° at an expense of less than 10 kJ/mol. Second, there are several local minima in the puckering of the ring systems that strongly affect the energies of these two dihedrals. Third, the results also depend on whether the phosphate group is included in the calculations or not (especially for MPD). Still, in our hands, the MPO model has a much more planar structure than in the previous study (ideal values for the α and β angles of 47 and 168°, compared to −8 and 112°; however, we can find a local minimum at −3 and 119°, that is 2 kJ/mol higher in energy than the more planar structure) and therefore more distant to the values found in the crystal structure than for the reduced structures.

**Table 4:** The α (C10–N8–C7–C6) and β (C10–N8–C7–O3′) dihedral angles of MPT in the various structures.

State	α				β			
	MPD	MPH	MPO	Cryst^a^	MPD	MPH	MPO	Cryst^a^
R	−29.1	−17.8	−11.3		90.0	102.0	106.9	
TS1	−32.6	−22.3	−13.1		86.7	97.4	105.5	
IM	−33.5	−24.6	−14.0		85.9	95.2	104.8	
TS2	−33.4	−22.0			86.0	98.0		
P	−33.0	−21.9	−13.7	−14.4, −19.5	86.5	98.0	104.9	95.7, 94.8
**Av**	−**32**.**3**	−**21**.**7**	−**13**.**0**	−**18**.**5**	**87**.**0**	**98**.**1**	**105**.**5**	**95**.**2**
Opt^b^	−23.1	−43.4	46.0		102.3	79.9	169.0	
Opt^c^	−47.6	−45.1	47.1		75.8	78.5	168.5	

^a^The two entries for the crystal structure^7^ represent the two subunits, both in the product-inhibited state with a Mo-bound water molecule.

^b^Optimised for the isolated full MPT model.

^c^Optimised for the isolated MPT, with the –CH_2_OPO_3_(H) group truncated to a H atom (so that the MPD and MPH models become identical).

The protonated phosphate groups of the MPH and MPO molecules form a favourable hydrogen bond to the backbone carbonyl group of Phe136 (~1.85 Å), as expected. However, there is no large change in the structure around the phosphate group in the MPD structure, although the O–O distance of 3.3 Å seems clearly to be unfavourable. The reason for this is probably the firm hydrogen bonds of the other two phosphate O atoms.

**Figure 6:**
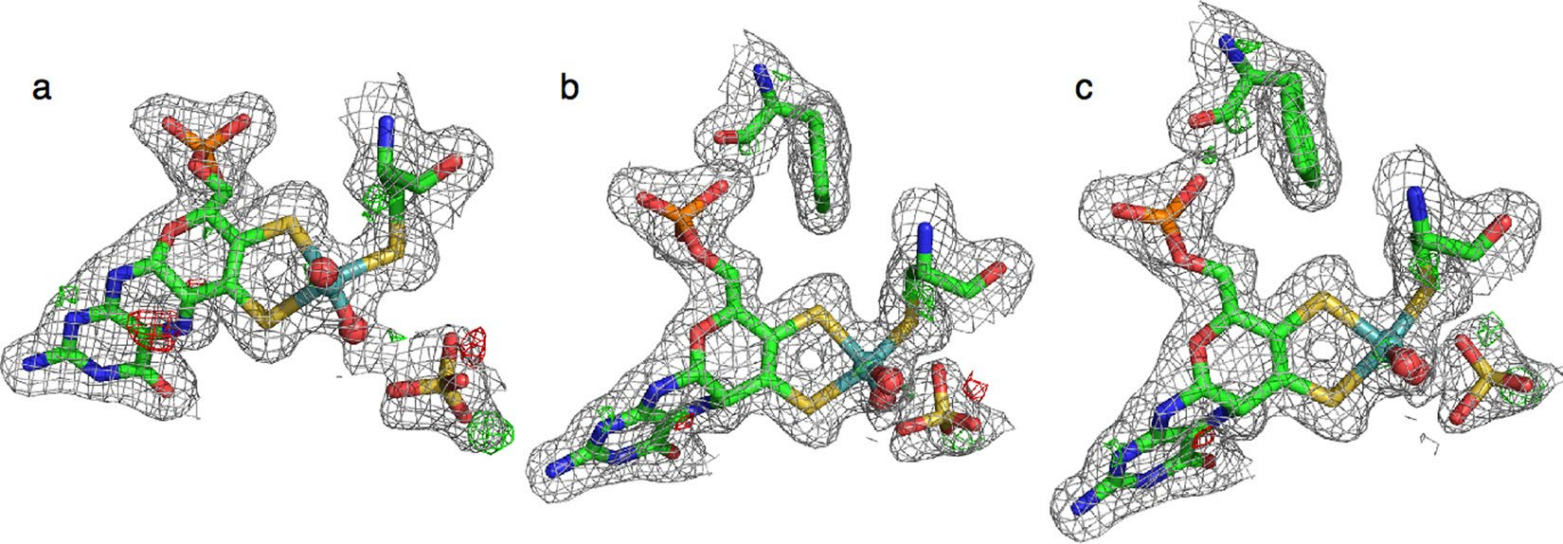
Results of the quantum refinements of sulfite oxidase with (**a**) MPD, (**b**) MPH and (**c**) MPO. The 2*mF*_o_ – *DF*_c_ maps are contoured at 1.0 σ (gray) and the *mF*_o_ – *DF*_c_ maps are contoured at +3.0 σ (green) and −3.0 σ (red).

To get some further evidence on which of the three MPT groups is most reasonable for sulfite oxidase, we performed quantum-refinement calculations with the three models in the QM system. Quantum refinement is standard crystallographic refinement, in which the MM potential (employed to ensure that bond lengths and angles are chemically reasonable) is replaced by more accurate QM calculations for the most interesting part of the structure. The calculations were performed on the product-inhibited state in the crystal structure, i.e. the P state with an extra Mo-bound water molecule. From Table 5, it can be seen that with the standard QM region, the MPD and MPH models fit the crystal structure better than the MPO model, especially around the MPT group. This is also clear from the electron-difference maps in Fig. 6, showing that the more planar structure of MPO gives rise to negative difference density (red) around the group. Thus, we can conclude that there is no evidence that the MPT group is oxidised in SO. However, we can of course not exclude the possibility that the MPT group is reduced by photoelectrons during the crystallographic data collection.

**Table 5:** Results of the quantum-refinement calculations, shown as the RSZD scores for the MPT molecule, Phe136, as well as the Mo and sulfate ions.

RSZD	Phe136 in MM			Phe136 in QM	
	MPD	MPH	MPO	MPD	MPH
MPT	1.1	1.1	2.5	0.9	1.0
Mo	4.2	4.0	4.1	4.5	4.1
${{\rm{SO}}}_{4}^{2-}$	2.9	3.1	3.5	3.7	3.5
Phe136	0.5	0.6	0.5	1.4	0.8
**Sum**	**8**.**7**	**8**.**8**	**10**.**6**	**10**.**5**	**9**.**4**

The calculations were performed with or without Phe136 in the QM system.

On the other hand, with this QM region, MPD and MPH give results of a similar quality. However, if the QM region is extended with the backbone of Phe136 (which receives the hydrogen bond from the phosphate group with the MPH model), the results become more conclusive: Then the RSZD scores of the MPD state is slightly higher than those of the MPH state, 10.5 compared to 9.4 for the sum over the four groups in Table 5. The largest differences come from Mo and Phe136. This is also seen in Fig. 6a, as a small extra volume of positive difference density (green) between the backbone O group of Phe136 and the MPD phosphate O atom, indicating that the two atoms should be closer than in the MPD structure. This indicates that MPH is the best model of MPT in sulfite oxidase, i.e. that the phosphate group is protonated, as was also indicated by the hydrogen-bond analysis.

## Conclusions

In this article, we have studied the reaction mechanism of sulfite oxidase with QM/MM methods. Three different reaction mechanisms have been suggested for this enzyme (the S → OMo, O → Mo and S → Mo mechanisms)^24^ and different experimental and computational studies have given conflicting results^7,12–24^. Recently, we studied all three mechanisms with the same QM-cluster method and both deprotonated and protonated substrate, and the results showed that the S → OMo mechanism gave the lowest barriers^24^. However, the results strongly depended on the dielectric constant assumed in the continuum solvation model and even a value of 80 (water) gave a too high activation barrier (139 kJ/mol). In this paper, we instead employ the QM/MM method, which includes an atomistic description of the surroundings, thereby avoiding the use of an ill-defined dielectric constant. For accurate energies, we have employed the big-QM^49^ and QTCP approaches^27,28^, with extrapolations to the def2-TZVPD basis set.

The results clearly show that the enzyme is set up for the S → OMo mechanism. An intricate network of hydrogen bonds involving Arg138, Arg190, Trp204, Tyr322, Arg450 and a water molecule provide proper solvation of the doubly negatively charged substrate and product. The hydrogen bonds vary somewhat during the reaction, but in each state, there are 8–10 hydrogen bonds shorter than 2.5 Å to the substrate or product, giving all states comparable energies. For the other reaction mechanisms, this hydrogen-bond network cannot be maintained, leading to unfavourable energies. In fact, we have not been able to find the key Mo–sulfite intermediate with either a Mo–O_Sub_ or Mo–S_Sub_ bond, in spite of extensive attempts including constrained structures and MD relaxation. Likewise, a protonated substrate also interferes with the hydrogen-bond network, in most cases leading to increased barriers. However, with the proton directed towards OH in Tyr322, a reasonable intermediate could be found, but then the proton had moved to Tyr322 and the HH proton on Tyr322 had moved to His40, i.e. with a deprotonated product.

The S → OMo mechanism is similar to that obtained in the QM-cluster calculations (Fig. 4): In the first step, the S_Sub_ atom of the substrate approaches the equatorial oxo group of Mo, forming a S_Sub_–O_eq_ bond and therefore directly a Mo-bound sulfate product. Concomitantly, the Mo ion is reduced from +VI to +IV. In the second step, the product dissociates. The first step is rate limiting with a barrier of 34–49 kJ/mol, which is much lower than in the previous QM-cluster calculations, owing to the favourable hydrogen-bond network involving three positively charged Arg residues, which compensates the net negative charge of the first coordination sphere of the Mo ion (−1). The intermediate and the product are appreciably more stable than the reactant state and of a similar energy, 41–76 kJ/mol. The second barrier is small. In the calculations, we have started and ended with states where the substrate or product binds in the second sphere of the Mo ion, as is shown in the product-inhibited crystal structure^7^. This avoids the problem of calculating the binding energy of the doubly charged substrate or product, which are hard to estimate accurately with QM/MM or continuum methods.

Finally, we have also examined the recent suggestion that the MPT group should be oxidised in SO to the 10,10a-dihydro state (MPO). We show that such a state gives a similar reaction mechanism (although the second transition state is not found). However, quantum-refinement calculations show that MPO fits the crystal structure appreciably worse than the normal reduced state. We also show that the α and β torsion angles employed to identify the oxidation state of the MPT group in crystal structures are problematic, showing multiple minima and involving rather small energies. Finally, we have investigated whether the phosphate group of MPT is protonated or not. We find a slight preference of the singly protonated structure (MPH), reflecting a hydrogen-bond interaction with the backbone O group of Phe136. In summary, our calculations strongly support the S → OMo mechanism and illustrate the importance of the protein surrounding in facilitating the oxygen atom transfer.

## Electronic supplementary material

Supplementary information
