# Supplementary material for: QM/MM study of the reaction mechanism of sulfite oxidase
Source: Sci Rep. 2018 Mar 16;8:4684. doi: 10.1038/s41598-018-22751-6 (PMC5856855; doi:10.1038/s41598-018-22751-6)
Supplement: Supplementary file 1 — Supplementary information [file 41598_2018_22751_MOESM1_ESM.pdf]

Supplementary Material

QM/MM study of the reaction mechanism of sulfite oxidase

Octav Caldararu,[1] Milica Feldt,[2] Daniella Cioloboc,[1]

Marie-Céline van Severen,[1] Kerstin Starke,[3] Ricardo A. Mata,[2]

Ebbe Nordlander,[3] Ulf Ryde [1,*]

[1] Department of Theoretical Chemistry, Lund University, Chemical Centre, P. O. Box 124,

SE-221 00 Lund, Sweden

[2] Institut für Physikalische Chemie, Universität Göttingen, Tammannstrasse 6, D-37077, Göttingen, Germany; nee Andrejić

[4] Department of Chemical Physics, Lund University, Chemical Centre, P. O. Box 124,

SE-221 00 Lund, Sweden

Correspondence to Ulf Ryde, E-mail: Ulf.Ryde@teokem.lu.se,

Tel: +46 – 46 2224502, Fax: +46 – 46 2228648

2018-02-13

Table S1. Examples of failures to find a Mo–OSO_2 intermediate, necessary for the O→Mo mechanism of sulfite oxidase, starting from the R (a) or IM (b) states. Neither curve shows any indication of a stable intermediate.

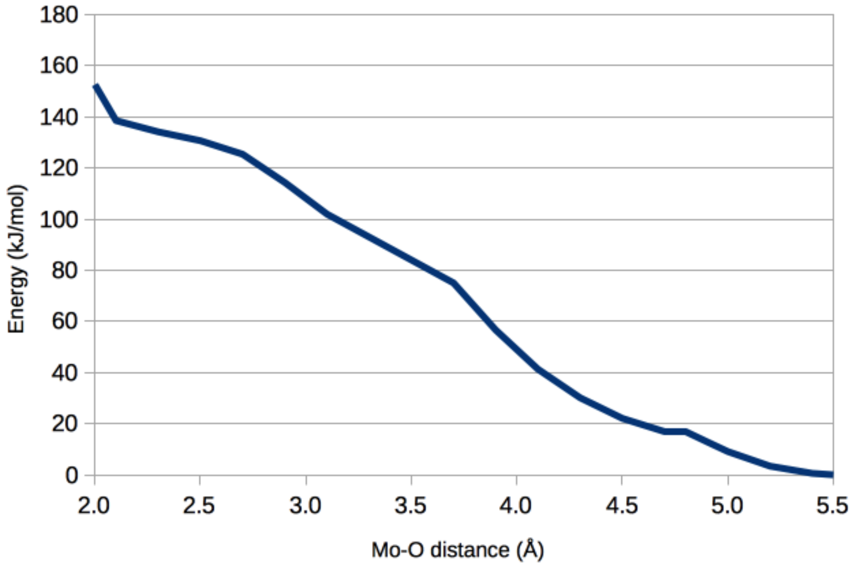

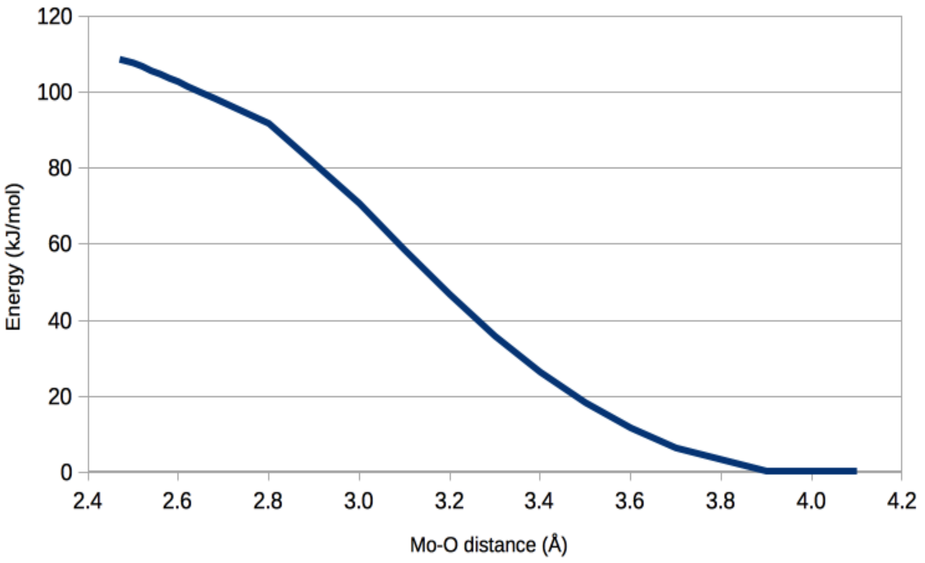

Table S2. Examples of failures to find a Mo–SO_3 intermediate, necessary for the S→Mo mechanism of sulfite oxidase, starting from the R state. The curve shows any indication of a stable intermediate.

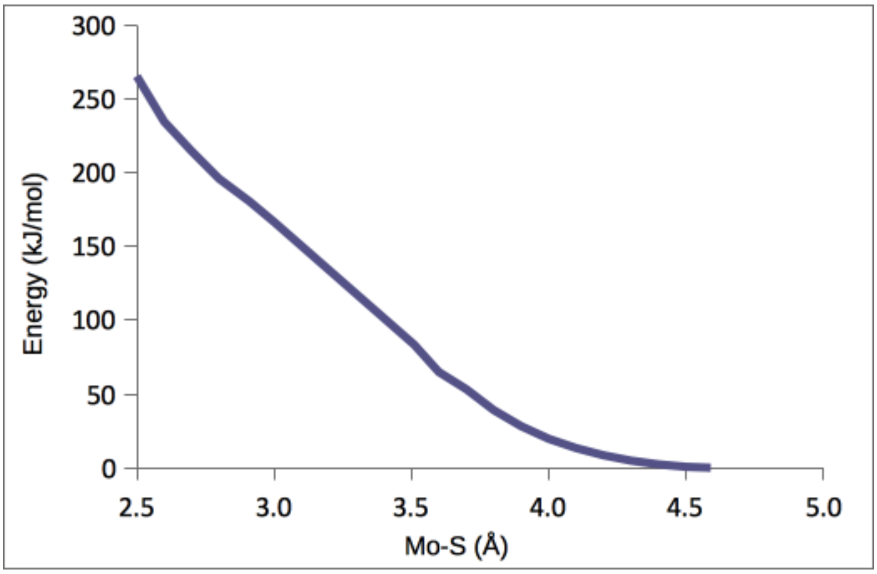

Table S1. Coordinates of the QM system (PDB format) for all intermediates discussed in the article.

```
MPD, state R
ATOM      1  H   ???     1       2.381   3.203   1.489
ATOM      2  C   ???     1       3.303   2.909   0.942
ATOM      3  H   ???     1       3.056   2.687  -0.111
ATOM      4  H   ???     1       4.045   3.719   0.979
ATOM      5  N   ???     1       3.857   1.709   1.569
ATOM      6  H   ???     1       3.307   0.848   1.462
ATOM      7  C   ???     1       4.950   1.652   2.352
ATOM      8  N   ???     1       5.759   2.730   2.497
ATOM      9  H   ???     1       5.372   3.677   2.422
ATOM     10  H   ???     1       6.568   2.641   3.130
ATOM     11  N   ???     1       5.268   0.501   2.954
ATOM     12  H   ???     1       4.605  -0.301   2.956
ATOM     13  H   ???     1       6.069   0.459   3.601
ATOM     14  H   ???     1       8.172   2.425  -1.063
ATOM     15  C   ???     1       7.434   1.637  -0.868
ATOM     16  N   ???     1       6.147   1.627  -1.366
ATOM     17  H   ???     1       5.751   2.363  -1.986
ATOM     18  C   ???     1       5.547   0.488  -0.919
ATOM     19  H   ???     1       4.510   0.229  -1.148
ATOM     20  N   ???     1       6.379  -0.226  -0.160
ATOM     21  C   ???     1       7.562   0.483  -0.117
ATOM     22  H   ???     1       8.427   0.102   0.433
ATOM     23  H   ???     1      -3.280   0.198   1.461
ATOM     24  C   ???     1      -2.680   0.814   2.167
ATOM     25  H   ???     1      -3.146   0.807   3.168
ATOM     26  H   ???     1      -2.678   1.842   1.769
ATOM     27  S   ???     1      -0.951   0.215   2.359
ATOM     28  H   ???     1      -2.837  -2.703   1.493
ATOM     29  C   ???     1      -2.835  -3.751   1.907
ATOM     30  H   ???     1      -3.770  -4.234   1.581
ATOM     31  H   ???     1      -1.965  -4.286   1.481
ATOM     32  C   ???     1      -2.752  -3.795   3.447
ATOM     33  O   ???     1      -3.346  -4.706   4.047
ATOM     34  N   ???     1      -1.987  -2.853   4.015
ATOM     35  H   ???     1      -1.489  -2.180   3.417
ATOM     36  C   ???     1      -1.797  -2.699   5.455
ATOM     37  H   ???     1      -1.805  -3.703   5.918
ATOM     38  H   ???     1      -0.798  -2.252   5.585
ATOM     39  H   ???     1      -2.546  -2.081   5.976
ATOM     40  H   ???     1       0.493  -3.546  10.227
ATOM     41  C   ???     1       1.464  -3.406   9.720
ATOM     42  H   ???     1       1.724  -2.340   9.734
ATOM     43  H   ???     1       2.237  -3.936  10.309
ATOM     44  N   ???     1       1.456  -3.928   8.369
ATOM     45  H   ???     1       1.501  -4.945   8.244
ATOM     46  C   ???     1       1.425  -3.213   7.227
ATOM     47  N   ???     1       1.370  -1.887   7.204
ATOM     48  H   ???     1       1.243  -1.326   8.054
ATOM     49  H   ???     1       1.592  -1.377   6.293
ATOM     50  N   ???     1       1.548  -3.891   6.058
ATOM     51  H   ???     1       1.425  -4.906   6.119
ATOM     52  H   ???     1       1.313  -3.403   5.128
ATOM     53  H   ???     1       3.322   1.103   8.351
ATOM     54  C   ???     1       2.982   1.685   7.460
ATOM     55  H   ???     1       2.133   1.134   7.017
ATOM     56  H   ???     1       2.596   2.667   7.799
ATOM     57  C   ???     1       4.065   1.903   6.379
ATOM     58  H   ???     1       4.464   0.925   6.033
ATOM     59  C   ???     1       3.380   2.536   5.161
ATOM     60  H   ???     1       2.772   3.409   5.461
ATOM     61  H   ???     1       4.127   2.868   4.423
ATOM     62  H   ???     1       2.712   1.801   4.677
ATOM     63  C   ???     1       5.260   2.753   6.853
ATOM     64  H   ???     1       5.817   2.264   7.679
ATOM     65  H   ???     1       5.974   2.904   6.016
ATOM     66  H   ???     1       4.922   3.747   7.206
ATOM     67  H   ???     1      -2.058   1.502   8.440
ATOM     68  C   ???     1      -1.369   1.700   7.614
ATOM     69  C   ???     1      -0.720   0.728   6.876
ATOM     70  H   ???     1      -0.689  -0.349   7.019
ATOM     71  N   ???     1      -0.077   1.303   5.800
ATOM     72  H   ???     1       0.561   0.786   5.182
ATOM     73  C   ???     1      -0.301   2.666   5.819
```

ATOM	74	C	???	1	0.061	3.662	4.900
ATOM	75	H	???	1	0.604	3.395	3.994
ATOM	76	C	???	1	-0.337	4.977	5.158
ATOM	77	H	???	1	-0.077	5.771	4.451
ATOM	78	C	???	1	-1.110	5.284	6.305
ATOM	79	H	???	1	-1.438	6.313	6.467
ATOM	80	C	???	1	-1.508	4.291	7.203
ATOM	81	H	???	1	-2.153	4.535	8.055
ATOM	82	C	???	1	-1.103	2.957	6.972
ATOM	83	H	???	1	1.851	-6.249	-1.675
ATOM	84	C	???	1	2.194	-5.265	-1.290
ATOM	85	C	???	1	1.699	-4.801	-0.055
ATOM	86	H	???	1	0.876	-5.329	0.451
ATOM	87	C	???	1	2.239	-3.683	0.584
ATOM	88	H	???	1	1.835	-3.341	1.545
ATOM	89	C	???	1	3.313	-2.990	-0.020
ATOM	90	O	???	1	3.927	-1.950	0.567
ATOM	91	H	???	1	3.615	-1.840	1.533
ATOM	92	C	???	1	3.772	-3.407	-1.290
ATOM	93	H	???	1	4.569	-2.825	-1.768
ATOM	94	C	???	1	3.231	-4.541	-1.906
ATOM	95	H	???	1	3.633	-4.859	-2.874
ATOM	96	H	???	1	5.092	-8.252	5.427
ATOM	97	C	???	1	5.599	-7.251	5.336
ATOM	98	H	???	1	5.523	-6.738	6.306
ATOM	99	H	???	1	6.672	-7.413	5.121
ATOM	100	N	???	1	5.022	-6.441	4.270
ATOM	101	H	???	1	5.213	-6.717	3.292
ATOM	102	C	???	1	4.428	-5.233	4.389
ATOM	103	N	???	1	4.281	-4.619	5.582
ATOM	104	H	???	1	4.492	-5.123	6.453
ATOM	105	H	???	1	3.458	-3.998	5.664
ATOM	106	N	???	1	3.950	-4.639	3.286
ATOM	107	H	???	1	4.146	-5.094	2.382
ATOM	108	H	???	1	3.653	-3.635	3.288
ATOM	109	S	???	1	1.943	-1.297	3.587
ATOM	110	O	???	1	1.161	-2.634	3.748
ATOM	111	O	???	1	2.114	-0.648	4.999
ATOM	112	O	???	1	3.462	-1.783	3.183
ATOM	113	S	???	1	-0.889	1.891	-0.701
ATOM	114	C	???	1	0.116	2.394	-2.079
ATOM	115	C	???	1	1.298	1.770	-2.330
ATOM	116	S	???	1	1.894	0.472	-1.311
ATOM	117	C	???	1	2.169	2.158	-3.501
ATOM	118	H	???	1	2.642	1.257	-3.939
ATOM	119	N	???	1	3.268	3.053	-3.103
ATOM	120	H	???	1	2.917	3.803	-2.487
ATOM	121	C	???	1	3.966	3.563	-4.239
ATOM	122	C	???	1	5.347	3.928	-4.129
ATOM	123	O	???	1	6.042	3.891	-3.094
ATOM	124	N	???	1	5.922	4.391	-5.340
ATOM	125	H	???	1	6.911	4.710	-5.313
ATOM	126	C	???	1	5.249	4.429	-6.533
ATOM	127	N	???	1	5.918	4.791	-7.653
ATOM	128	H	???	1	6.878	5.182	-7.608
ATOM	129	H	???	1	5.371	5.031	-8.485
ATOM	130	N	???	1	3.971	4.070	-6.629
ATOM	131	C	???	1	3.347	3.660	-5.491
ATOM	132	N	???	1	2.039	3.259	-5.660
ATOM	133	H	???	1	1.561	3.569	-6.509
ATOM	134	C	???	1	1.249	2.800	-4.559
ATOM	135	H	???	1	0.512	2.063	-4.935
ATOM	136	O	???	1	0.571	3.899	-3.965
ATOM	137	C	???	1	-0.408	3.503	-2.983
ATOM	138	H	???	1	-1.309	3.134	-3.528
ATOM	139	C	???	1	-0.775	4.773	-2.228
ATOM	140	H	???	1	0.119	5.144	-1.690
ATOM	141	H	???	1	-1.550	4.537	-1.468
ATOM	142	O	???	1	-1.221	5.728	-3.159
ATOM	143	P	???	1	-1.466	7.310	-2.475
ATOM	144	O	???	1	-0.888	8.206	-3.631
ATOM	145	O	???	1	-0.496	7.346	-1.285
ATOM	146	O	???	1	-2.961	7.469	-2.290
ATOM	147	MO	???	1	0.097	-0.215	0.200
ATOM	148	O	???	1	-0.857	-1.511	-0.524
ATOM	149	O	???	1	1.502	-0.790	1.095
ATOM	150	O	???	1	6.609	-2.373	1.373
ATOM	151	H	???	1	6.308	-1.623	0.698
ATOM	152	H	???	1	6.342	-3.284	1.085

```
ATOM    153  O   ???     1       6.039  -2.226   3.887
ATOM    154  H   ???     1       5.047  -2.090   3.970
ATOM    155  H   ???     1       6.185  -2.234   2.869
ATOM    156  O   ???     1       7.662  -0.270   4.661
ATOM    157  H   ???     1       7.082  -1.046   4.359
ATOM    158  H   ???     1       7.569  -0.305   5.666
ATOM    159  O   ???     1       9.240  -2.032   1.587
ATOM    160  H   ???     1       9.573  -2.951   1.449
ATOM    161  H   ???     1       8.244  -2.167   1.629
ATOM    162  O   ???     1       7.540  -4.498   3.782
ATOM    163  H   ???     1       6.973  -3.714   4.042
ATOM    164  H   ???     1       7.063  -4.815   2.970
END
MPD, state Ts1
ATOM      1  H   ???     1       2.381   3.211   1.492
ATOM      2  C   ???     1       3.308   2.933   0.950
ATOM      3  H   ???     1       3.071   2.709  -0.104
ATOM      4  H   ???     1       4.048   3.744   1.000
ATOM      5  N   ???     1       3.854   1.733   1.584
ATOM      6  H   ???     1       3.255   0.896   1.525
ATOM      7  C   ???     1       4.952   1.671   2.348
ATOM      8  N   ???     1       5.754   2.747   2.517
ATOM      9  H   ???     1       5.360   3.695   2.463
ATOM     10  H   ???     1       6.586   2.648   3.117
ATOM     11  N   ???     1       5.289   0.507   2.928
ATOM     12  H   ???     1       4.687  -0.319   2.825
ATOM     13  H   ???     1       6.087   0.449   3.575
ATOM     14  H   ???     1       8.171   2.427  -1.063
ATOM     15  C   ???     1       7.432   1.643  -0.868
ATOM     16  N   ???     1       6.144   1.644  -1.364
ATOM     17  H   ???     1       5.749   2.389  -1.979
ATOM     18  C   ???     1       5.530   0.516  -0.919
ATOM     19  H   ???     1       4.486   0.271  -1.137
ATOM     20  N   ???     1       6.358  -0.204  -0.157
ATOM     21  C   ???     1       7.550   0.490  -0.115
ATOM     22  H   ???     1       8.410   0.102   0.437
ATOM     23  H   ???     1      -3.287   0.196   1.473
ATOM     24  C   ???     1      -2.703   0.807   2.203
ATOM     25  H   ???     1      -3.250   0.858   3.162
ATOM     26  H   ???     1      -2.620   1.816   1.767
ATOM     27  S   ???     1      -1.023   0.121   2.520
ATOM     28  H   ???     1      -2.843  -2.704   1.487
ATOM     29  C   ???     1      -2.842  -3.751   1.903
ATOM     30  H   ???     1      -3.775  -4.239   1.576
ATOM     31  H   ???     1      -1.968  -4.286   1.480
ATOM     32  C   ???     1      -2.755  -3.796   3.442
ATOM     33  O   ???     1      -3.316  -4.728   4.044
ATOM     34  N   ???     1      -2.008  -2.838   4.005
ATOM     35  H   ???     1      -1.600  -2.093   3.414
ATOM     36  C   ???     1      -1.805  -2.686   5.441
ATOM     37  H   ???     1      -1.786  -3.689   5.905
ATOM     38  H   ???     1      -0.819  -2.205   5.557
ATOM     39  H   ???     1      -2.550  -2.077   5.972
ATOM     40  H   ???     1       0.488  -3.538  10.197
ATOM     41  C   ???     1       1.442  -3.382   9.642
ATOM     42  H   ???     1       1.706  -2.319   9.688
ATOM     43  H   ???     1       2.238  -3.941  10.168
ATOM     44  N   ???     1       1.382  -3.850   8.260
ATOM     45  H   ???     1       1.413  -4.866   8.114
ATOM     46  C   ???     1       1.359  -3.122   7.128
ATOM     47  N   ???     1       1.381  -1.789   7.101
ATOM     48  H   ???     1       1.223  -1.216   7.942
ATOM     49  H   ???     1       1.405  -1.288   6.184
ATOM     50  N   ???     1       1.436  -3.804   5.948
ATOM     51  H   ???     1       1.229  -4.806   6.003
ATOM     52  H   ???     1       1.135  -3.338   5.053
ATOM     53  H   ???     1       3.313   1.102   8.350
ATOM     54  C   ???     1       2.958   1.681   7.459
ATOM     55  H   ???     1       2.084   1.148   7.039
ATOM     56  H   ???     1       2.592   2.669   7.803
ATOM     57  C   ???     1       4.014   1.886   6.349
ATOM     58  H   ???     1       4.396   0.903   5.992
ATOM     59  C   ???     1       3.303   2.530   5.151
ATOM     60  H   ???     1       2.719   3.412   5.471
ATOM     61  H   ???     1       4.035   2.853   4.394
ATOM     62  H   ???     1       2.605   1.814   4.682
ATOM     63  C   ???     1       5.229   2.720   6.796
ATOM     64  H   ???     1       5.801   2.222   7.606
ATOM     65  H   ???     1       5.922   2.870   5.943
```

ATOM	66	H	???	1	4.906	3.714	7.163
ATOM	67	H	???	1	-2.061	1.512	8.434
ATOM	68	C	???	1	-1.384	1.732	7.598
ATOM	69	C	???	1	-0.800	0.789	6.773
ATOM	70	H	???	1	-0.805	-0.295	6.845
ATOM	71	N	???	1	-0.186	1.407	5.706
ATOM	72	H	???	1	0.263	0.903	4.934
ATOM	73	C	???	1	-0.379	2.767	5.799
ATOM	74	C	???	1	-0.025	3.795	4.910
ATOM	75	H	???	1	0.487	3.566	3.974
ATOM	76	C	???	1	-0.393	5.102	5.242
ATOM	77	H	???	1	-0.150	5.925	4.563
ATOM	78	C	???	1	-1.124	5.364	6.427
ATOM	79	H	???	1	-1.432	6.386	6.651
ATOM	80	C	???	1	-1.501	4.340	7.298
ATOM	81	H	???	1	-2.114	4.558	8.176
ATOM	82	C	???	1	-1.121	3.014	7.001
ATOM	83	H	???	1	1.855	-6.255	-1.687
ATOM	84	C	???	1	2.207	-5.276	-1.321
ATOM	85	C	???	1	1.639	-4.740	-0.153
ATOM	86	H	???	1	0.801	-5.252	0.341
ATOM	87	C	???	1	2.122	-3.563	0.417
ATOM	88	H	???	1	1.657	-3.163	1.321
ATOM	89	C	???	1	3.207	-2.887	-0.185
ATOM	90	O	???	1	3.718	-1.753	0.326
ATOM	91	H	???	1	3.029	-1.346	0.953
ATOM	92	C	???	1	3.781	-3.414	-1.360
ATOM	93	H	???	1	4.593	-2.851	-1.835
ATOM	94	C	???	1	3.286	-4.598	-1.917
ATOM	95	H	???	1	3.740	-4.978	-2.833
ATOM	96	H	???	1	5.090	-8.252	5.428
ATOM	97	C	???	1	5.595	-7.250	5.339
ATOM	98	H	???	1	5.517	-6.733	6.306
ATOM	99	H	???	1	6.668	-7.408	5.122
ATOM	100	N	???	1	5.019	-6.447	4.266
ATOM	101	H	???	1	5.231	-6.719	3.290
ATOM	102	C	???	1	4.384	-5.263	4.373
ATOM	103	N	???	1	4.197	-4.651	5.564
ATOM	104	H	???	1	4.419	-5.137	6.443
ATOM	105	H	???	1	3.369	-4.037	5.619
ATOM	106	N	???	1	3.897	-4.689	3.262
ATOM	107	H	???	1	4.130	-5.139	2.363
ATOM	108	H	???	1	3.611	-3.689	3.250
ATOM	109	S	???	1	1.892	-1.398	3.399
ATOM	110	O	???	1	0.980	-2.612	3.463
ATOM	111	O	???	1	1.661	-0.456	4.571
ATOM	112	O	???	1	3.362	-1.855	3.375
ATOM	113	S	???	1	-0.825	1.774	-0.562
ATOM	114	C	???	1	0.209	2.307	-1.939
ATOM	115	C	???	1	1.407	1.698	-2.141
ATOM	116	S	???	1	1.985	0.414	-1.054
ATOM	117	C	???	1	2.281	2.042	-3.334
ATOM	118	H	???	1	2.780	1.127	-3.709
ATOM	119	N	???	1	3.354	2.999	-3.004
ATOM	120	H	???	1	2.975	3.766	-2.426
ATOM	121	C	???	1	4.008	3.502	-4.175
ATOM	122	C	???	1	5.374	3.919	-4.092
ATOM	123	O	???	1	6.088	3.907	-3.069
ATOM	124	N	???	1	5.918	4.389	-5.312
ATOM	125	H	???	1	6.902	4.723	-5.302
ATOM	126	C	???	1	5.222	4.414	-6.492
ATOM	127	N	???	1	5.876	4.810	-7.613
ATOM	128	H	???	1	6.835	5.206	-7.574
ATOM	129	H	???	1	5.328	5.025	-8.451
ATOM	130	N	???	1	3.954	4.012	-6.572
ATOM	131	C	???	1	3.374	3.547	-5.424
ATOM	132	N	???	1	2.107	3.024	-5.575
ATOM	133	H	???	1	1.580	3.345	-6.391
ATOM	134	C	???	1	1.344	2.596	-4.432
ATOM	135	H	???	1	0.634	1.813	-4.764
ATOM	136	O	???	1	0.643	3.711	-3.915
ATOM	137	C	???	1	-0.316	3.393	-2.880
ATOM	138	H	???	1	-1.246	3.031	-3.377
ATOM	139	C	???	1	-0.614	4.733	-2.204
ATOM	140	H	???	1	0.306	5.086	-1.699
ATOM	141	H	???	1	-1.390	4.579	-1.422
ATOM	142	O	???	1	-1.016	5.663	-3.179
ATOM	143	P	???	1	-1.445	7.233	-2.488
ATOM	144	O	???	1	-0.906	8.189	-3.621

```
ATOM    145  O   ???     1      -0.503   7.335  -1.278
ATOM    146  O   ???     1      -2.952   7.206  -2.357
ATOM    147 MO   ???     1       0.177  -0.199   0.436
ATOM    148  O   ???     1      -0.613  -1.572  -0.296
ATOM    149  O   ???     1       1.764  -0.681   1.648
ATOM    150  O   ???     1       6.468  -2.307   1.363
ATOM    151  H   ???     1       6.238  -1.509   0.671
ATOM    152  H   ???     1       6.215  -3.201   1.026
ATOM    153  O   ???     1       6.068  -2.244   3.876
ATOM    154  H   ???     1       5.106  -2.131   4.075
ATOM    155  H   ???     1       6.127  -2.232   2.836
ATOM    156  O   ???     1       7.667  -0.270   4.659
ATOM    157  H   ???     1       7.113  -1.058   4.348
ATOM    158  H   ???     1       7.571  -0.307   5.663
ATOM    159  O   ???     1       9.141  -2.073   1.611
ATOM    160  H   ???     1       9.427  -3.013   1.490
ATOM    161  H   ???     1       8.139  -2.154   1.625
ATOM    162  O   ???     1       7.526  -4.534   3.746
ATOM    163  H   ???     1       6.987  -3.729   3.999
ATOM    164  H   ???     1       7.058  -4.821   2.914
END
MPD, state Im
ATOM      1  H   ???     1       2.388   3.215   1.495
ATOM      2  C   ???     1       3.324   2.947   0.959
ATOM      3  H   ???     1       3.095   2.706  -0.093
ATOM      4  H   ???     1       4.041   3.778   0.999
ATOM      5  N   ???     1       3.915   1.776   1.605
ATOM      6  H   ???     1       3.365   0.913   1.537
ATOM      7  C   ???     1       5.040   1.743   2.336
ATOM      8  N   ???     1       5.817   2.839   2.480
ATOM      9  H   ???     1       5.404   3.777   2.414
ATOM     10  H   ???     1       6.643   2.761   3.089
ATOM     11  N   ???     1       5.431   0.588   2.892
ATOM     12  H   ???     1       4.765  -0.201   2.938
ATOM     13  H   ???     1       6.220   0.567   3.556
ATOM     14  H   ???     1       8.171   2.427  -1.064
ATOM     15  C   ???     1       7.430   1.641  -0.871
ATOM     16  N   ???     1       6.142   1.648  -1.365
ATOM     17  H   ???     1       5.745   2.397  -1.972
ATOM     18  C   ???     1       5.529   0.514  -0.931
ATOM     19  H   ???     1       4.485   0.273  -1.150
ATOM     20  N   ???     1       6.358  -0.215  -0.180
ATOM     21  C   ???     1       7.549   0.480  -0.131
ATOM     22  H   ???     1       8.410   0.088   0.417
ATOM     23  H   ???     1      -3.291   0.195   1.473
ATOM     24  C   ???     1      -2.711   0.804   2.203
ATOM     25  H   ???     1      -3.266   0.865   3.157
ATOM     26  H   ???     1      -2.612   1.811   1.763
ATOM     27  S   ???     1      -1.044   0.089   2.526
ATOM     28  H   ???     1      -2.844  -2.705   1.486
ATOM     29  C   ???     1      -2.841  -3.750   1.904
ATOM     30  H   ???     1      -3.775  -4.239   1.581
ATOM     31  H   ???     1      -1.969  -4.286   1.480
ATOM     32  C   ???     1      -2.747  -3.792   3.442
ATOM     33  O   ???     1      -3.298  -4.726   4.049
ATOM     34  N   ???     1      -1.994  -2.832   4.001
ATOM     35  H   ???     1      -1.638  -2.052   3.413
ATOM     36  C   ???     1      -1.796  -2.674   5.438
ATOM     37  H   ???     1      -1.768  -3.677   5.903
ATOM     38  H   ???     1      -0.817  -2.181   5.554
ATOM     39  H   ???     1      -2.547  -2.072   5.970
ATOM     40  H   ???     1       0.487  -3.536  10.199
ATOM     41  C   ???     1       1.440  -3.376   9.647
ATOM     42  H   ???     1       1.701  -2.313   9.697
ATOM     43  H   ???     1       2.238  -3.935  10.171
ATOM     44  N   ???     1       1.381  -3.840   8.265
ATOM     45  H   ???     1       1.429  -4.856   8.115
ATOM     46  C   ???     1       1.327  -3.113   7.133
ATOM     47  N   ???     1       1.376  -1.782   7.088
ATOM     48  H   ???     1       1.266  -1.198   7.929
ATOM     49  H   ???     1       1.329  -1.302   6.159
ATOM     50  N   ???     1       1.346  -3.810   5.961
ATOM     51  H   ???     1       1.044  -4.787   6.012
ATOM     52  H   ???     1       1.182  -3.331   5.040
ATOM     53  H   ???     1       3.312   1.100   8.347
ATOM     54  C   ???     1       2.955   1.674   7.451
ATOM     55  H   ???     1       2.077   1.139   7.039
ATOM     56  H   ???     1       2.591   2.665   7.788
ATOM     57  C   ???     1       4.005   1.869   6.333
```

ATOM	58	H	???	1	4.394	0.885	5.987
ATOM	59	C	???	1	3.286	2.490	5.126
ATOM	60	H	???	1	2.698	3.376	5.431
ATOM	61	H	???	1	4.012	2.805	4.360
ATOM	62	H	???	1	2.587	1.764	4.671
ATOM	63	C	???	1	5.214	2.719	6.765
ATOM	64	H	???	1	5.791	2.235	7.580
ATOM	65	H	???	1	5.904	2.863	5.908
ATOM	66	H	???	1	4.885	3.715	7.120
ATOM	67	H	???	1	-2.062	1.514	8.433
ATOM	68	C	???	1	-1.387	1.736	7.595
ATOM	69	C	???	1	-0.817	0.795	6.759
ATOM	70	H	???	1	-0.823	-0.288	6.828
ATOM	71	N	???	1	-0.212	1.413	5.688
ATOM	72	H	???	1	0.192	0.907	4.891
ATOM	73	C	???	1	-0.397	2.774	5.787
ATOM	74	C	???	1	-0.047	3.803	4.899
ATOM	75	H	???	1	0.456	3.574	3.957
ATOM	76	C	???	1	-0.405	5.110	5.239
ATOM	77	H	???	1	-0.165	5.935	4.561
ATOM	78	C	???	1	-1.123	5.371	6.432
ATOM	79	H	???	1	-1.423	6.394	6.664
ATOM	80	C	???	1	-1.495	4.347	7.304
ATOM	81	H	???	1	-2.097	4.564	8.189
ATOM	82	C	???	1	-1.125	3.020	6.999
ATOM	83	H	???	1	1.855	-6.255	-1.689
ATOM	84	C	???	1	2.206	-5.273	-1.327
ATOM	85	C	???	1	1.587	-4.711	-0.201
ATOM	86	H	???	1	0.724	-5.212	0.259
ATOM	87	C	???	1	2.056	-3.528	0.373
ATOM	88	H	???	1	1.569	-3.117	1.259
ATOM	89	C	???	1	3.186	-2.886	-0.177
ATOM	90	O	???	1	3.713	-1.767	0.360
ATOM	91	H	???	1	3.059	-1.373	1.011
ATOM	92	C	???	1	3.804	-3.432	-1.319
ATOM	93	H	???	1	4.651	-2.891	-1.757
ATOM	94	C	???	1	3.318	-4.616	-1.883
ATOM	95	H	???	1	3.814	-5.019	-2.766
ATOM	96	H	???	1	5.096	-8.252	5.425
ATOM	97	C	???	1	5.610	-7.253	5.329
ATOM	98	H	???	1	5.555	-6.729	6.295
ATOM	99	H	???	1	6.677	-7.420	5.096
ATOM	100	N	???	1	5.028	-6.449	4.261
ATOM	101	H	???	1	5.243	-6.705	3.283
ATOM	102	C	???	1	4.273	-5.339	4.389
ATOM	103	N	???	1	4.040	-4.772	5.589
ATOM	104	H	???	1	4.306	-5.245	6.462
ATOM	105	H	???	1	3.197	-4.180	5.664
ATOM	106	N	???	1	3.743	-4.795	3.282
ATOM	107	H	???	1	3.999	-5.211	2.374
ATOM	108	H	???	1	3.136	-3.957	3.305
ATOM	109	S	???	1	2.050	-1.375	3.399
ATOM	110	O	???	1	1.524	-2.813	3.400
ATOM	111	O	???	1	1.382	-0.586	4.495
ATOM	112	O	???	1	3.554	-1.404	3.582
ATOM	113	S	???	1	-0.821	1.742	-0.541
ATOM	114	C	???	1	0.228	2.292	-1.912
ATOM	115	C	???	1	1.433	1.691	-2.092
ATOM	116	S	???	1	1.977	0.383	-0.991
ATOM	117	C	???	1	2.309	2.021	-3.288
ATOM	118	H	???	1	2.813	1.102	-3.647
ATOM	119	N	???	1	3.376	2.992	-2.978
ATOM	120	H	???	1	2.993	3.762	-2.407
ATOM	121	C	???	1	4.019	3.491	-4.158
ATOM	122	C	???	1	5.380	3.923	-4.081
ATOM	123	O	???	1	6.097	3.923	-3.060
ATOM	124	N	???	1	5.917	4.390	-5.306
ATOM	125	H	???	1	6.900	4.728	-5.300
ATOM	126	C	???	1	5.216	4.408	-6.482
ATOM	127	N	???	1	5.866	4.811	-7.603
ATOM	128	H	???	1	6.825	5.208	-7.566
ATOM	129	H	???	1	5.319	5.017	-8.444
ATOM	130	N	???	1	3.951	3.994	-6.558
ATOM	131	C	???	1	3.382	3.521	-5.406
ATOM	132	N	???	1	2.126	2.969	-5.551
ATOM	133	H	???	1	1.589	3.285	-6.362
ATOM	134	C	???	1	1.369	2.553	-4.397
ATOM	135	H	???	1	0.663	1.761	-4.718
ATOM	136	O	???	1	0.662	3.673	-3.902

```
ATOM    137  C   ???     1      -0.295   3.371  -2.860
ATOM    138  H   ???     1      -1.228   3.007  -3.350
ATOM    139  C   ???     1      -0.586   4.722  -2.199
ATOM    140  H   ???     1       0.338   5.075  -1.701
ATOM    141  H   ???     1      -1.360   4.579  -1.413
ATOM    142  O   ???     1      -0.987   5.648  -3.180
ATOM    143  P   ???     1      -1.444   7.213  -2.490
ATOM    144  O   ???     1      -0.910   8.181  -3.617
ATOM    145  O   ???     1      -0.510   7.327  -1.274
ATOM    146  O   ???     1      -2.952   7.161  -2.372
ATOM    147 MO   ???     1       0.136  -0.218   0.426
ATOM    148  O   ???     1      -0.682  -1.557  -0.332
ATOM    149  O   ???     1       1.818  -0.739   1.972
ATOM    150  O   ???     1       6.489  -2.324   1.351
ATOM    151  H   ???     1       6.243  -1.539   0.665
ATOM    152  H   ???     1       6.239  -3.225   1.029
ATOM    153  O   ???     1       6.113  -2.282   3.880
ATOM    154  H   ???     1       5.149  -2.161   4.072
ATOM    155  H   ???     1       6.168  -2.255   2.845
ATOM    156  O   ???     1       7.678  -0.274   4.660
ATOM    157  H   ???     1       7.119  -1.060   4.348
ATOM    158  H   ???     1       7.580  -0.311   5.663
ATOM    159  O   ???     1       9.157  -2.073   1.607
ATOM    160  H   ???     1       9.446  -3.012   1.490
ATOM    161  H   ???     1       8.155  -2.158   1.618
ATOM    162  O   ???     1       7.550  -4.564   3.759
ATOM    163  H   ???     1       7.014  -3.754   4.007
ATOM    164  H   ???     1       7.073  -4.857   2.936
END
MPD, state Ts2
ATOM      1  H   ???     1       2.388   3.205   1.501
ATOM      2  C   ???     1       3.324   2.916   0.978
ATOM      3  H   ???     1       3.098   2.640  -0.066
ATOM      4  H   ???     1       4.044   3.746   0.993
ATOM      5  N   ???     1       3.896   1.764   1.678
ATOM      6  H   ???     1       3.299   0.928   1.763
ATOM      7  C   ???     1       5.029   1.743   2.394
ATOM      8  N   ???     1       5.839   2.821   2.468
ATOM      9  H   ???     1       5.437   3.764   2.394
ATOM     10  H   ???     1       6.671   2.748   3.071
ATOM     11  N   ???     1       5.393   0.606   3.010
ATOM     12  H   ???     1       4.734  -0.174   3.054
ATOM     13  H   ???     1       6.192   0.575   3.665
ATOM     14  H   ???     1       8.169   2.428  -1.065
ATOM     15  C   ???     1       7.425   1.646  -0.875
ATOM     16  N   ???     1       6.135   1.665  -1.362
ATOM     17  H   ???     1       5.745   2.414  -1.974
ATOM     18  C   ???     1       5.514   0.536  -0.924
ATOM     19  H   ???     1       4.468   0.300  -1.140
ATOM     20  N   ???     1       6.340  -0.200  -0.177
ATOM     21  C   ???     1       7.537   0.484  -0.135
ATOM     22  H   ???     1       8.397   0.085   0.410
ATOM     23  H   ???     1      -3.277   0.191   1.469
ATOM     24  C   ???     1      -2.671   0.790   2.188
ATOM     25  H   ???     1      -3.167   0.823   3.175
ATOM     26  H   ???     1      -2.586   1.806   1.770
ATOM     27  S   ???     1      -0.981   0.049   2.389
ATOM     28  H   ???     1      -2.843  -2.705   1.486
ATOM     29  C   ???     1      -2.841  -3.751   1.902
ATOM     30  H   ???     1      -3.774  -4.239   1.574
ATOM     31  H   ???     1      -1.968  -4.286   1.479
ATOM     32  C   ???     1      -2.753  -3.796   3.441
ATOM     33  O   ???     1      -3.315  -4.727   4.041
ATOM     34  N   ???     1      -1.997  -2.841   4.005
ATOM     35  H   ???     1      -1.621  -2.079   3.414
ATOM     36  C   ???     1      -1.800  -2.690   5.445
ATOM     37  H   ???     1      -1.793  -3.695   5.903
ATOM     38  H   ???     1      -0.809  -2.227   5.568
ATOM     39  H   ???     1      -2.548  -2.078   5.972
ATOM     40  H   ???     1       0.496  -3.539  10.224
ATOM     41  C   ???     1       1.470  -3.386   9.718
ATOM     42  H   ???     1       1.720  -2.318   9.738
ATOM     43  H   ???     1       2.246  -3.908  10.308
ATOM     44  N   ???     1       1.484  -3.906   8.365
ATOM     45  H   ???     1       1.509  -4.925   8.251
ATOM     46  C   ???     1       1.361  -3.216   7.211
ATOM     47  N   ???     1       1.341  -1.881   7.170
ATOM     48  H   ???     1       1.203  -1.307   8.012
ATOM     49  H   ???     1       1.308  -1.392   6.247
```

ATOM	50	N	???	1	1.323	-3.932	6.074
ATOM	51	H	???	1	1.461	-4.943	6.147
ATOM	52	H	???	1	1.333	-3.497	5.126
ATOM	53	H	???	1	3.312	1.099	8.348
ATOM	54	C	???	1	2.953	1.672	7.454
ATOM	55	H	???	1	2.087	1.122	7.039
ATOM	56	H	???	1	2.577	2.658	7.791
ATOM	57	C	???	1	4.006	1.875	6.341
ATOM	58	H	???	1	4.393	0.893	5.993
ATOM	59	C	???	1	3.286	2.501	5.137
ATOM	60	H	???	1	2.687	3.376	5.446
ATOM	61	H	???	1	4.012	2.831	4.377
ATOM	62	H	???	1	2.599	1.770	4.671
ATOM	63	C	???	1	5.213	2.727	6.776
ATOM	64	H	???	1	5.790	2.242	7.590
ATOM	65	H	???	1	5.904	2.874	5.920
ATOM	66	H	???	1	4.883	3.722	7.134
ATOM	67	H	???	1	-2.062	1.512	8.433
ATOM	68	C	???	1	-1.385	1.730	7.596
ATOM	69	C	???	1	-0.802	0.782	6.776
ATOM	70	H	???	1	-0.793	-0.301	6.863
ATOM	71	N	???	1	-0.201	1.392	5.698
ATOM	72	H	???	1	0.269	0.878	4.945
ATOM	73	C	???	1	-0.390	2.754	5.785
ATOM	74	C	???	1	-0.037	3.779	4.893
ATOM	75	H	???	1	0.474	3.545	3.956
ATOM	76	C	???	1	-0.399	5.088	5.221
ATOM	77	H	???	1	-0.152	5.908	4.541
ATOM	78	C	???	1	-1.124	5.356	6.409
ATOM	79	H	???	1	-1.426	6.381	6.631
ATOM	80	C	???	1	-1.501	4.337	7.284
ATOM	81	H	???	1	-2.108	4.559	8.166
ATOM	82	C	???	1	-1.126	3.008	6.990
ATOM	83	H	???	1	1.851	-6.247	-1.683
ATOM	84	C	???	1	2.193	-5.259	-1.315
ATOM	85	C	???	1	1.627	-4.723	-0.145
ATOM	86	H	???	1	0.799	-5.245	0.356
ATOM	87	C	???	1	2.108	-3.540	0.428
ATOM	88	H	???	1	1.678	-3.154	1.359
ATOM	89	C	???	1	3.200	-2.874	-0.170
ATOM	90	O	???	1	3.756	-1.767	0.354
ATOM	91	H	???	1	3.244	-1.442	1.162
ATOM	92	C	???	1	3.753	-3.385	-1.364
ATOM	93	H	???	1	4.566	-2.823	-1.837
ATOM	94	C	???	1	3.256	-4.566	-1.923
ATOM	95	H	???	1	3.708	-4.942	-2.844
ATOM	96	H	???	1	5.086	-8.246	5.427
ATOM	97	C	???	1	5.580	-7.236	5.334
ATOM	98	H	???	1	5.506	-6.717	6.301
ATOM	99	H	???	1	6.651	-7.373	5.094
ATOM	100	N	???	1	4.945	-6.455	4.276
ATOM	101	H	???	1	5.088	-6.766	3.300
ATOM	102	C	???	1	4.402	-5.221	4.359
ATOM	103	N	???	1	4.228	-4.580	5.530
ATOM	104	H	???	1	4.355	-5.023	6.447
ATOM	105	H	???	1	3.868	-3.621	5.484
ATOM	106	N	???	1	4.008	-4.617	3.229
ATOM	107	H	???	1	4.214	-5.097	2.341
ATOM	108	H	???	1	3.272	-3.879	3.258
ATOM	109	S	???	1	2.321	-1.444	3.757
ATOM	110	O	???	1	1.798	-2.871	3.524
ATOM	111	O	???	1	1.385	-0.689	4.662
ATOM	112	O	???	1	3.697	-1.558	4.421
ATOM	113	S	???	1	-0.879	1.745	-0.664
ATOM	114	C	???	1	0.197	2.315	-2.004
ATOM	115	C	???	1	1.403	1.720	-2.167
ATOM	116	S	???	1	1.914	0.377	-1.084
ATOM	117	C	???	1	2.289	2.053	-3.351
ATOM	118	H	???	1	2.786	1.133	-3.718
ATOM	119	N	???	1	3.361	3.008	-3.020
ATOM	120	H	???	1	2.986	3.774	-2.438
ATOM	121	C	???	1	4.015	3.509	-4.191
ATOM	122	C	???	1	5.380	3.931	-4.105
ATOM	123	O	???	1	6.088	3.927	-3.078
ATOM	124	N	???	1	5.926	4.394	-5.326
ATOM	125	H	???	1	6.910	4.730	-5.314
ATOM	126	C	???	1	5.233	4.415	-6.508
ATOM	127	N	???	1	5.885	4.810	-7.629
ATOM	128	H	???	1	6.844	5.207	-7.591

```
ATOM    129  H   ???     1       5.337   5.021  -8.468
ATOM    130  N   ???     1       3.967   4.009  -6.589
ATOM    131  C   ???     1       3.387   3.546  -5.443
ATOM    132  N   ???     1       2.124   3.013  -5.602
ATOM    133  H   ???     1       1.601   3.333  -6.420
ATOM    134  C   ???     1       1.356   2.601  -4.460
ATOM    135  H   ???     1       0.643   1.817  -4.785
ATOM    136  O   ???     1       0.655   3.725  -3.958
ATOM    137  C   ???     1      -0.313   3.406  -2.935
ATOM    138  H   ???     1      -1.243   3.054  -3.440
ATOM    139  C   ???     1      -0.610   4.731  -2.234
ATOM    140  H   ???     1       0.313   5.077  -1.727
ATOM    141  H   ???     1      -1.381   4.560  -1.449
ATOM    142  O   ???     1      -1.024   5.672  -3.190
ATOM    143  P   ???     1      -1.447   7.232  -2.489
ATOM    144  O   ???     1      -0.908   8.191  -3.617
ATOM    145  O   ???     1      -0.508   7.323  -1.277
ATOM    146  O   ???     1      -2.954   7.212  -2.354
ATOM    147 MO   ???     1       0.021  -0.235   0.208
ATOM    148  O   ???     1      -0.853  -1.530  -0.568
ATOM    149  O   ???     1       2.443  -0.729   2.395
ATOM    150  O   ???     1       6.490  -2.331   1.325
ATOM    151  H   ???     1       6.232  -1.550   0.645
ATOM    152  H   ???     1       6.240  -3.237   1.017
ATOM    153  O   ???     1       6.223  -2.268   3.867
ATOM    154  H   ???     1       5.266  -2.119   4.118
ATOM    155  H   ???     1       6.236  -2.249   2.836
ATOM    156  O   ???     1       7.691  -0.246   4.668
ATOM    157  H   ???     1       7.169  -1.056   4.337
ATOM    158  H   ???     1       7.585  -0.297   5.670
ATOM    159  O   ???     1       9.157  -2.073   1.602
ATOM    160  H   ???     1       9.446  -3.013   1.491
ATOM    161  H   ???     1       8.156  -2.156   1.600
ATOM    162  O   ???     1       7.556  -4.598   3.761
ATOM    163  H   ???     1       7.049  -3.761   3.987
ATOM    164  H   ???     1       7.069  -4.895   2.945
END
MPD, state P
REMARK Enlarged QM system for sulfite oxidase (Arg44, Hid46, Cym81, Gly93, Arg96, Leu10
REMARK Energy =   -5999.258419
REMARK /home/octav/Abisko/Sulfox/Mpt/Ps
REMARK Thu Jan 25 11:11:37 CET 2018
ATOM      1  H   ???     1       2.391   3.207   1.499
ATOM      2  C   ???     1       3.331   2.923   0.975
ATOM      3  H   ???     1       3.102   2.645  -0.069
ATOM      4  H   ???     1       4.042   3.760   0.983
ATOM      5  N   ???     1       3.930   1.782   1.667
ATOM      6  H   ???     1       3.363   0.929   1.740
ATOM      7  C   ???     1       5.068   1.773   2.382
ATOM      8  N   ???     1       5.847   2.871   2.482
ATOM      9  H   ???     1       5.419   3.804   2.438
ATOM     10  H   ???     1       6.685   2.799   3.077
ATOM     11  N   ???     1       5.460   0.630   2.960
ATOM     12  H   ???     1       4.804  -0.162   2.923
ATOM     13  H   ???     1       6.248   0.593   3.626
ATOM     14  H   ???     1       8.168   2.427  -1.065
ATOM     15  C   ???     1       7.423   1.643  -0.874
ATOM     16  N   ???     1       6.133   1.662  -1.364
ATOM     17  H   ???     1       5.744   2.411  -1.975
ATOM     18  C   ???     1       5.512   0.533  -0.926
ATOM     19  H   ???     1       4.467   0.297  -1.144
ATOM     20  N   ???     1       6.335  -0.204  -0.178
ATOM     21  C   ???     1       7.532   0.481  -0.134
ATOM     22  H   ???     1       8.390   0.082   0.414
ATOM     23  H   ???     1      -3.273   0.190   1.469
ATOM     24  C   ???     1      -2.660   0.783   2.187
ATOM     25  H   ???     1      -3.135   0.805   3.184
ATOM     26  H   ???     1      -2.573   1.803   1.779
ATOM     27  S   ???     1      -0.967   0.024   2.340
ATOM     28  H   ???     1      -2.844  -2.706   1.485
ATOM     29  C   ???     1      -2.843  -3.752   1.900
ATOM     30  H   ???     1      -3.774  -4.241   1.569
ATOM     31  H   ???     1      -1.968  -4.287   1.478
ATOM     32  C   ???     1      -2.758  -3.799   3.439
ATOM     33  O   ???     1      -3.322  -4.729   4.037
ATOM     34  N   ???     1      -2.002  -2.845   4.004
ATOM     35  H   ???     1      -1.623  -2.088   3.411
ATOM     36  C   ???     1      -1.804  -2.694   5.445
ATOM     37  H   ???     1      -1.798  -3.698   5.903
```

ATOM	38	H	???	1	-0.810	-2.236	5.569
ATOM	39	H	???	1	-2.550	-2.080	5.973
ATOM	40	H	???	1	0.498	-3.540	10.232
ATOM	41	C	???	1	1.479	-3.387	9.739
ATOM	42	H	???	1	1.729	-2.319	9.759
ATOM	43	H	???	1	2.247	-3.905	10.344
ATOM	44	N	???	1	1.517	-3.916	8.392
ATOM	45	H	???	1	1.554	-4.935	8.283
ATOM	46	C	???	1	1.339	-3.235	7.239
ATOM	47	N	???	1	1.256	-1.904	7.204
ATOM	48	H	???	1	1.174	-1.331	8.052
ATOM	49	H	???	1	1.281	-1.401	6.291
ATOM	50	N	???	1	1.278	-3.962	6.110
ATOM	51	H	???	1	1.517	-4.954	6.187
ATOM	52	H	???	1	1.342	-3.525	5.166
ATOM	53	H	???	1	3.314	1.098	8.348
ATOM	54	C	???	1	2.960	1.671	7.452
ATOM	55	H	???	1	2.101	1.115	7.030
ATOM	56	H	???	1	2.578	2.656	7.785
ATOM	57	C	???	1	4.022	1.875	6.347
ATOM	58	H	???	1	4.410	0.892	6.003
ATOM	59	C	???	1	3.316	2.505	5.138
ATOM	60	H	???	1	2.715	3.381	5.444
ATOM	61	H	???	1	4.050	2.835	4.386
ATOM	62	H	???	1	2.634	1.776	4.662
ATOM	63	C	???	1	5.227	2.725	6.794
ATOM	64	H	???	1	5.798	2.237	7.611
ATOM	65	H	???	1	5.925	2.870	5.943
ATOM	66	H	???	1	4.897	3.720	7.150
ATOM	67	H	???	1	-2.061	1.510	8.433
ATOM	68	C	???	1	-1.384	1.726	7.597
ATOM	69	C	???	1	-0.791	0.775	6.788
ATOM	70	H	???	1	-0.780	-0.307	6.884
ATOM	71	N	???	1	-0.184	1.382	5.711
ATOM	72	H	???	1	0.320	0.871	4.978
ATOM	73	C	???	1	-0.376	2.744	5.790
ATOM	74	C	???	1	-0.019	3.766	4.897
ATOM	75	H	???	1	0.501	3.529	3.967
ATOM	76	C	???	1	-0.387	5.076	5.217
ATOM	77	H	???	1	-0.136	5.894	4.535
ATOM	78	C	???	1	-1.122	5.348	6.396
ATOM	79	H	???	1	-1.430	6.373	6.611
ATOM	80	C	???	1	-1.504	4.331	7.273
ATOM	81	H	???	1	-2.119	4.555	8.150
ATOM	82	C	???	1	-1.124	3.002	6.987
ATOM	83	H	???	1	1.849	-6.245	-1.680
ATOM	84	C	???	1	2.185	-5.253	-1.306
ATOM	85	C	???	1	1.658	-4.744	-0.104
ATOM	86	H	???	1	0.859	-5.284	0.425
ATOM	87	C	???	1	2.141	-3.561	0.468
ATOM	88	H	???	1	1.742	-3.193	1.422
ATOM	89	C	???	1	3.190	-2.862	-0.168
ATOM	90	O	???	1	3.739	-1.745	0.341
ATOM	91	H	???	1	3.391	-1.525	1.262
ATOM	92	C	???	1	3.699	-3.342	-1.395
ATOM	93	H	???	1	4.481	-2.758	-1.893
ATOM	94	C	???	1	3.207	-4.528	-1.949
ATOM	95	H	???	1	3.630	-4.883	-2.893
ATOM	96	H	???	1	5.090	-8.252	5.430
ATOM	97	C	???	1	5.592	-7.249	5.342
ATOM	98	H	???	1	5.517	-6.729	6.309
ATOM	99	H	???	1	6.664	-7.397	5.110
ATOM	100	N	???	1	4.973	-6.463	4.282
ATOM	101	H	???	1	5.121	-6.768	3.305
ATOM	102	C	???	1	4.412	-5.238	4.377
ATOM	103	N	???	1	4.186	-4.621	5.547
ATOM	104	H	???	1	4.368	-5.056	6.459
ATOM	105	H	???	1	3.904	-3.623	5.503
ATOM	106	N	???	1	4.042	-4.621	3.242
ATOM	107	H	???	1	4.254	-5.103	2.356
ATOM	108	H	???	1	3.285	-3.906	3.273
ATOM	109	S	???	1	2.541	-1.518	3.995
ATOM	110	O	???	1	1.889	-2.856	3.609
ATOM	111	O	???	1	1.557	-0.648	4.728
ATOM	112	O	???	1	3.732	-1.830	4.921
ATOM	113	S	???	1	-0.920	1.760	-0.709
ATOM	114	C	???	1	0.174	2.337	-2.032
ATOM	115	C	???	1	1.380	1.743	-2.187
ATOM	116	S	???	1	1.868	0.378	-1.116

```
ATOM    117  C   ???     1       2.270   2.077  -3.366
ATOM    118  H   ???     1       2.762   1.155  -3.737
ATOM    119  N   ???     1       3.348   3.022  -3.030
ATOM    120  H   ???     1       2.982   3.788  -2.443
ATOM    121  C   ???     1       4.009   3.521  -4.198
ATOM    122  C   ???     1       5.376   3.936  -4.109
ATOM    123  O   ???     1       6.082   3.931  -3.081
ATOM    124  N   ???     1       5.927   4.396  -5.330
ATOM    125  H   ???     1       6.911   4.729  -5.316
ATOM    126  C   ???     1       5.237   4.417  -6.513
ATOM    127  N   ???     1       5.891   4.807  -7.634
ATOM    128  H   ???     1       6.850   5.204  -7.596
ATOM    129  H   ???     1       5.343   5.021  -8.473
ATOM    130  N   ???     1       3.969   4.017  -6.597
ATOM    131  C   ???     1       3.384   3.562  -5.451
ATOM    132  N   ???     1       2.115   3.044  -5.613
ATOM    133  H   ???     1       1.599   3.361  -6.437
ATOM    134  C   ???     1       1.343   2.634  -4.476
ATOM    135  H   ???     1       0.625   1.855  -4.803
ATOM    136  O   ???     1       0.643   3.760  -3.970
ATOM    137  C   ???     1      -0.331   3.430  -2.958
ATOM    138  H   ???     1      -1.256   3.078  -3.472
ATOM    139  C   ???     1      -0.639   4.740  -2.240
ATOM    140  H   ???     1       0.282   5.092  -1.733
ATOM    141  H   ???     1      -1.404   4.553  -1.454
ATOM    142  O   ???     1      -1.070   5.687  -3.185
ATOM    143  P   ???     1      -1.451   7.247  -2.485
ATOM    144  O   ???     1      -0.905   8.194  -3.618
ATOM    145  O   ???     1      -0.506   7.327  -1.278
ATOM    146  O   ???     1      -2.957   7.272  -2.335
ATOM    147 MO   ???     1      -0.052  -0.243   0.119
ATOM    148  O   ???     1      -0.938  -1.531  -0.658
ATOM    149  O   ???     1       3.035  -0.837   2.713
ATOM    150  O   ???     1       6.428  -2.325   1.383
ATOM    151  H   ???     1       6.202  -1.565   0.682
ATOM    152  H   ???     1       6.219  -3.238   1.062
ATOM    153  O   ???     1       6.200  -2.245   3.940
ATOM    154  H   ???     1       5.271  -2.118   4.303
ATOM    155  H   ???     1       6.141  -2.237   2.916
ATOM    156  O   ???     1       7.707  -0.240   4.673
ATOM    157  H   ???     1       7.172  -1.047   4.353
ATOM    158  H   ???     1       7.598  -0.284   5.676
ATOM    159  O   ???     1       9.118  -2.076   1.609
ATOM    160  H   ???     1       9.389  -3.023   1.506
ATOM    161  H   ???     1       8.117  -2.143   1.644
ATOM    162  O   ???     1       7.576  -4.553   3.774
ATOM    163  H   ???     1       7.063  -3.722   4.012
ATOM    164  H   ???     1       7.075  -4.861   2.973
END
MPH, state R
ATOM      1  H   ???     1       2.385   3.205   1.494
ATOM      2  C   ???     1       3.316   2.916   0.957
ATOM      3  H   ???     1       3.088   2.701  -0.101
ATOM      4  H   ???     1       4.054   3.729   1.013
ATOM      5  N   ???     1       3.865   1.718   1.588
ATOM      6  H   ???     1       3.309   0.860   1.492
ATOM      7  C   ???     1       4.961   1.660   2.370
ATOM      8  N   ???     1       5.775   2.733   2.507
ATOM      9  H   ???     1       5.393   3.683   2.429
ATOM     10  H   ???     1       6.586   2.644   3.137
ATOM     11  N   ???     1       5.270   0.510   2.977
ATOM     12  H   ???     1       4.602  -0.286   2.970
ATOM     13  H   ???     1       6.077   0.456   3.618
ATOM     14  H   ???     1       8.172   2.422  -1.060
ATOM     15  C   ???     1       7.438   1.630  -0.859
ATOM     16  N   ???     1       6.148   1.617  -1.350
ATOM     17  H   ???     1       5.749   2.356  -1.962
ATOM     18  C   ???     1       5.552   0.476  -0.903
ATOM     19  H   ???     1       4.518   0.212  -1.131
ATOM     20  N   ???     1       6.389  -0.238  -0.150
ATOM     21  C   ???     1       7.571   0.474  -0.111
ATOM     22  H   ???     1       8.439   0.093   0.434
ATOM     23  H   ???     1      -3.283   0.199   1.466
ATOM     24  C   ???     1      -2.691   0.815   2.181
ATOM     25  H   ???     1      -3.148   0.771   3.186
ATOM     26  H   ???     1      -2.725   1.852   1.810
ATOM     27  S   ???     1      -0.941   0.279   2.356
ATOM     28  H   ???     1      -2.836  -2.702   1.493
ATOM     29  C   ???     1      -2.833  -3.751   1.908
```

ATOM	30	H	???	1	-3.770	-4.233	1.583
ATOM	31	H	???	1	-1.964	-4.286	1.481
ATOM	32	C	???	1	-2.750	-3.792	3.448
ATOM	33	O	???	1	-3.351	-4.695	4.050
ATOM	34	N	???	1	-1.981	-2.852	4.016
ATOM	35	H	???	1	-1.446	-2.211	3.418
ATOM	36	C	???	1	-1.795	-2.696	5.458
ATOM	37	H	???	1	-1.801	-3.699	5.923
ATOM	38	H	???	1	-0.799	-2.244	5.589
ATOM	39	H	???	1	-2.546	-2.080	5.977
ATOM	40	H	???	1	0.492	-3.545	10.223
ATOM	41	C	???	1	1.462	-3.403	9.713
ATOM	42	H	???	1	1.724	-2.338	9.731
ATOM	43	H	???	1	2.238	-3.939	10.291
ATOM	44	N	???	1	1.444	-3.920	8.356
ATOM	45	H	???	1	1.485	-4.937	8.230
ATOM	46	C	???	1	1.416	-3.206	7.217
ATOM	47	N	???	1	1.388	-1.878	7.190
ATOM	48	H	???	1	1.262	-1.312	8.039
ATOM	49	H	???	1	1.556	-1.372	6.274
ATOM	50	N	???	1	1.522	-3.887	6.044
ATOM	51	H	???	1	1.361	-4.898	6.105
ATOM	52	H	???	1	1.265	-3.405	5.126
ATOM	53	H	???	1	3.321	1.102	8.351
ATOM	54	C	???	1	2.979	1.684	7.460
ATOM	55	H	???	1	2.128	1.135	7.020
ATOM	56	H	???	1	2.595	2.666	7.801
ATOM	57	C	???	1	4.060	1.903	6.377
ATOM	58	H	???	1	4.460	0.925	6.030
ATOM	59	C	???	1	3.371	2.537	5.161
ATOM	60	H	???	1	2.765	3.409	5.466
ATOM	61	H	???	1	4.117	2.871	4.423
ATOM	62	H	???	1	2.701	1.805	4.677
ATOM	63	C	???	1	5.256	2.752	6.849
ATOM	64	H	???	1	5.816	2.261	7.671
ATOM	65	H	???	1	5.966	2.907	6.010
ATOM	66	H	???	1	4.918	3.744	7.208
ATOM	67	H	???	1	-2.060	1.503	8.439
ATOM	68	C	???	1	-1.374	1.702	7.611
ATOM	69	C	???	1	-0.728	0.732	6.871
ATOM	70	H	???	1	-0.695	-0.345	7.016
ATOM	71	N	???	1	-0.090	1.305	5.791
ATOM	72	H	???	1	0.551	0.784	5.176
ATOM	73	C	???	1	-0.312	2.668	5.812
ATOM	74	C	???	1	0.053	3.666	4.897
ATOM	75	H	???	1	0.602	3.401	3.993
ATOM	76	C	???	1	-0.342	4.981	5.159
ATOM	77	H	???	1	-0.071	5.780	4.461
ATOM	78	C	???	1	-1.115	5.287	6.307
ATOM	79	H	???	1	-1.436	6.318	6.478
ATOM	80	C	???	1	-1.514	4.293	7.203
ATOM	81	H	???	1	-2.154	4.536	8.058
ATOM	82	C	???	1	-1.111	2.959	6.968
ATOM	83	H	???	1	1.851	-6.248	-1.677
ATOM	84	C	???	1	2.193	-5.263	-1.294
ATOM	85	C	???	1	1.693	-4.791	-0.066
ATOM	86	H	???	1	0.876	-5.322	0.445
ATOM	87	C	???	1	2.222	-3.660	0.562
ATOM	88	H	???	1	1.816	-3.318	1.520
ATOM	89	C	???	1	3.292	-2.966	-0.048
ATOM	90	O	???	1	3.892	-1.909	0.530
ATOM	91	H	???	1	3.585	-1.806	1.495
ATOM	92	C	???	1	3.759	-3.392	-1.310
ATOM	93	H	???	1	4.557	-2.814	-1.791
ATOM	94	C	???	1	3.226	-4.537	-1.916
ATOM	95	H	???	1	3.634	-4.863	-2.878
ATOM	96	H	???	1	5.095	-8.255	5.429
ATOM	97	C	???	1	5.608	-7.259	5.340
ATOM	98	H	???	1	5.536	-6.745	6.309
ATOM	99	H	???	1	6.679	-7.428	5.123
ATOM	100	N	???	1	5.039	-6.445	4.271
ATOM	101	H	???	1	5.231	-6.721	3.293
ATOM	102	C	???	1	4.412	-5.257	4.393
ATOM	103	N	???	1	4.251	-4.647	5.586
ATOM	104	H	???	1	4.489	-5.140	6.457
ATOM	105	H	???	1	3.406	-4.054	5.673
ATOM	106	N	???	1	3.913	-4.675	3.290
ATOM	107	H	???	1	4.122	-5.125	2.386
ATOM	108	H	???	1	3.612	-3.679	3.290

```
ATOM    109  S   ???     1       1.875  -1.304   3.530
ATOM    110  O   ???     1       1.102  -2.641   3.696
ATOM    111  O   ???     1       1.987  -0.607   4.919
ATOM    112  O   ???     1       3.396  -1.749   3.158
ATOM    113  S   ???     1      -1.008   1.908  -0.694
ATOM    114  C   ???     1      -0.046   2.436  -2.090
ATOM    115  C   ???     1       1.169   1.882  -2.350
ATOM    116  S   ???     1       1.839   0.615  -1.339
ATOM    117  C   ???     1       2.028   2.342  -3.505
ATOM    118  H   ???     1       2.481   1.464  -4.008
ATOM    119  N   ???     1       3.150   3.187  -3.068
ATOM    120  H   ???     1       2.841   3.923  -2.415
ATOM    121  C   ???     1       3.891   3.685  -4.183
ATOM    122  C   ???     1       5.297   3.962  -4.077
ATOM    123  O   ???     1       5.987   3.887  -3.044
ATOM    124  N   ???     1       5.895   4.396  -5.289
ATOM    125  H   ???     1       6.894   4.682  -5.263
ATOM    126  C   ???     1       5.230   4.449  -6.487
ATOM    127  N   ???     1       5.915   4.778  -7.605
ATOM    128  H   ???     1       6.888   5.142  -7.564
ATOM    129  H   ???     1       5.381   5.022  -8.446
ATOM    130  N   ???     1       3.932   4.150  -6.578
ATOM    131  C   ???     1       3.280   3.831  -5.431
ATOM    132  N   ???     1       1.918   3.620  -5.568
ATOM    133  H   ???     1       1.520   3.733  -6.501
ATOM    134  C   ???     1       1.120   3.055  -4.529
ATOM    135  H   ???     1       0.394   2.342  -4.972
ATOM    136  O   ???     1       0.376   4.089  -3.849
ATOM    137  C   ???     1      -0.626   3.512  -3.002
ATOM    138  H   ???     1      -1.406   3.060  -3.661
ATOM    139  C   ???     1      -1.263   4.626  -2.185
ATOM    140  H   ???     1      -0.561   4.987  -1.416
ATOM    141  H   ???     1      -2.174   4.244  -1.694
ATOM    142  O   ???     1      -1.617   5.726  -3.053
ATOM    143  P   ???     1      -1.428   7.220  -2.386
ATOM    144  O   ???     1      -1.029   8.122  -3.558
ATOM    145  O   ???     1      -0.447   7.212  -1.250
ATOM    146  O   ???     1      -2.916   7.627  -1.866
ATOM    147  H   ???     1      -2.946   7.785  -0.884
ATOM    148 MO   ???     1       0.097  -0.146   0.212
ATOM    149  O   ???     1      -0.810  -1.478  -0.493
ATOM    150  O   ???     1       1.529  -0.663   1.101
ATOM    151  O   ???     1       6.580  -2.401   1.395
ATOM    152  H   ???     1       6.299  -1.663   0.709
ATOM    153  H   ???     1       6.351  -3.323   1.101
ATOM    154  O   ???     1       5.992  -2.234   3.897
ATOM    155  H   ???     1       5.005  -2.101   3.988
ATOM    156  H   ???     1       6.137  -2.254   2.879
ATOM    157  O   ???     1       7.639  -0.277   4.660
ATOM    158  H   ???     1       7.064  -1.057   4.368
ATOM    159  H   ???     1       7.560  -0.310   5.667
ATOM    160  O   ???     1       9.224  -2.025   1.585
ATOM    161  H   ???     1       9.575  -2.937   1.441
ATOM    162  H   ???     1       8.234  -2.179   1.649
ATOM    163  O   ???     1       7.536  -4.489   3.789
ATOM    164  H   ???     1       6.957  -3.720   4.057
ATOM    165  H   ???     1       7.063  -4.817   2.979
END
MPH, state Ts1
ATOM      1  H   ???     1       2.385   3.211   1.496
ATOM      2  C   ???     1       3.318   2.934   0.962
ATOM      3  H   ???     1       3.096   2.719  -0.098
ATOM      4  H   ???     1       4.056   3.746   1.029
ATOM      5  N   ???     1       3.856   1.732   1.595
ATOM      6  H   ???     1       3.251   0.899   1.546
ATOM      7  C   ???     1       4.958   1.664   2.359
ATOM      8  N   ???     1       5.768   2.736   2.515
ATOM      9  H   ???     1       5.380   3.685   2.463
ATOM     10  H   ???     1       6.599   2.636   3.118
ATOM     11  N   ???     1       5.282   0.501   2.942
ATOM     12  H   ???     1       4.660  -0.316   2.860
ATOM     13  H   ???     1       6.083   0.439   3.589
ATOM     14  H   ???     1       8.172   2.425  -1.061
ATOM     15  C   ???     1       7.435   1.637  -0.863
ATOM     16  N   ???     1       6.148   1.637  -1.360
ATOM     17  H   ???     1       5.757   2.383  -1.970
ATOM     18  C   ???     1       5.535   0.505  -0.919
ATOM     19  H   ???     1       4.497   0.254  -1.147
ATOM     20  N   ???     1       6.362  -0.216  -0.160
```

ATOM	21	C	???	1	7.552	0.481	-0.113
ATOM	22	H	???	1	8.413	0.091	0.438
ATOM	23	H	???	1	-3.288	0.197	1.475
ATOM	24	C	???	1	-2.708	0.809	2.207
ATOM	25	H	???	1	-3.237	0.822	3.177
ATOM	26	H	???	1	-2.668	1.830	1.795
ATOM	27	S	???	1	-1.000	0.187	2.499
ATOM	28	H	???	1	-2.841	-2.703	1.488
ATOM	29	C	???	1	-2.839	-3.751	1.905
ATOM	30	H	???	1	-3.774	-4.236	1.578
ATOM	31	H	???	1	-1.967	-4.286	1.480
ATOM	32	C	???	1	-2.753	-3.794	3.444
ATOM	33	O	???	1	-3.324	-4.715	4.047
ATOM	34	N	???	1	-1.999	-2.839	4.008
ATOM	35	H	???	1	-1.551	-2.128	3.412
ATOM	36	C	???	1	-1.800	-2.683	5.445
ATOM	37	H	???	1	-1.784	-3.686	5.912
ATOM	38	H	???	1	-0.814	-2.204	5.564
ATOM	39	H	???	1	-2.548	-2.075	5.973
ATOM	40	H	???	1	0.489	-3.540	10.199
ATOM	41	C	???	1	1.444	-3.388	9.648
ATOM	42	H	???	1	1.712	-2.326	9.692
ATOM	43	H	???	1	2.237	-3.949	10.177
ATOM	44	N	???	1	1.384	-3.861	8.267
ATOM	45	H	???	1	1.414	-4.878	8.123
ATOM	46	C	???	1	1.366	-3.133	7.138
ATOM	47	N	???	1	1.392	-1.801	7.113
ATOM	48	H	???	1	1.245	-1.231	7.958
ATOM	49	H	???	1	1.436	-1.299	6.196
ATOM	50	N	???	1	1.442	-3.811	5.953
ATOM	51	H	???	1	1.222	-4.811	6.003
ATOM	52	H	???	1	1.144	-3.336	5.062
ATOM	53	H	???	1	3.316	1.102	8.350
ATOM	54	C	???	1	2.965	1.681	7.459
ATOM	55	H	???	1	2.097	1.144	7.033
ATOM	56	H	???	1	2.595	2.667	7.802
ATOM	57	C	???	1	4.030	1.891	6.358
ATOM	58	H	???	1	4.420	0.910	6.004
ATOM	59	C	???	1	3.327	2.531	5.153
ATOM	60	H	???	1	2.736	3.410	5.469
ATOM	61	H	???	1	4.064	2.858	4.404
ATOM	62	H	???	1	2.638	1.810	4.678
ATOM	63	C	???	1	5.238	2.731	6.815
ATOM	64	H	???	1	5.806	2.234	7.628
ATOM	65	H	???	1	5.937	2.883	5.966
ATOM	66	H	???	1	4.910	3.723	7.181
ATOM	67	H	???	1	-2.063	1.511	8.436
ATOM	68	C	???	1	-1.386	1.728	7.602
ATOM	69	C	???	1	-0.780	0.780	6.801
ATOM	70	H	???	1	-0.773	-0.302	6.893
ATOM	71	N	???	1	-0.160	1.389	5.732
ATOM	72	H	???	1	0.358	0.878	5.007
ATOM	73	C	???	1	-0.363	2.749	5.807
ATOM	74	C	???	1	0.000	3.773	4.918
ATOM	75	H	???	1	0.535	3.538	3.996
ATOM	76	C	???	1	-0.378	5.081	5.234
ATOM	77	H	???	1	-0.116	5.902	4.560
ATOM	78	C	???	1	-1.129	5.350	6.405
ATOM	79	H	???	1	-1.438	6.375	6.619
ATOM	80	C	???	1	-1.516	4.331	7.277
ATOM	81	H	???	1	-2.140	4.553	8.147
ATOM	82	C	???	1	-1.126	3.005	6.994
ATOM	83	H	???	1	1.855	-6.253	-1.688
ATOM	84	C	???	1	2.205	-5.270	-1.326
ATOM	85	C	???	1	1.661	-4.737	-0.145
ATOM	86	H	???	1	0.848	-5.259	0.379
ATOM	87	C	???	1	2.140	-3.546	0.403
ATOM	88	H	???	1	1.697	-3.149	1.320
ATOM	89	C	???	1	3.190	-2.855	-0.240
ATOM	90	O	???	1	3.689	-1.698	0.237
ATOM	91	H	???	1	3.026	-1.315	0.897
ATOM	92	C	???	1	3.743	-3.382	-1.424
ATOM	93	H	???	1	4.534	-2.810	-1.924
ATOM	94	C	???	1	3.254	-4.579	-1.957
ATOM	95	H	???	1	3.690	-4.958	-2.882
ATOM	96	H	???	1	5.092	-8.253	5.429
ATOM	97	C	???	1	5.600	-7.253	5.340
ATOM	98	H	???	1	5.524	-6.737	6.308
ATOM	99	H	???	1	6.671	-7.414	5.122

```
ATOM    100  N   ???     1       5.026  -6.445   4.267
ATOM    101  H   ???     1       5.236  -6.718   3.292
ATOM    102  C   ???     1       4.382  -5.268   4.379
ATOM    103  N   ???     1       4.194  -4.658   5.570
ATOM    104  H   ???     1       4.432  -5.141   6.447
ATOM    105  H   ???     1       3.350  -4.066   5.635
ATOM    106  N   ???     1       3.884  -4.696   3.268
ATOM    107  H   ???     1       4.118  -5.145   2.369
ATOM    108  H   ???     1       3.601  -3.698   3.254
ATOM    109  S   ???     1       1.865  -1.356   3.390
ATOM    110  O   ???     1       0.979  -2.597   3.464
ATOM    111  O   ???     1       1.686  -0.477   4.637
ATOM    112  O   ???     1       3.346  -1.793   3.286
ATOM    113  S   ???     1      -0.938   1.799  -0.579
ATOM    114  C   ???     1       0.059   2.358  -1.958
ATOM    115  C   ???     1       1.281   1.806  -2.189
ATOM    116  S   ???     1       1.929   0.537  -1.136
ATOM    117  C   ???     1       2.133   2.230  -3.373
ATOM    118  H   ???     1       2.601   1.335  -3.830
ATOM    119  N   ???     1       3.240   3.126  -3.000
ATOM    120  H   ???     1       2.917   3.876  -2.370
ATOM    121  C   ???     1       3.936   3.624  -4.146
ATOM    122  C   ???     1       5.330   3.955  -4.064
ATOM    123  O   ???     1       6.037   3.907  -3.041
ATOM    124  N   ???     1       5.899   4.397  -5.285
ATOM    125  H   ???     1       6.895   4.698  -5.272
ATOM    126  C   ???     1       5.214   4.440  -6.471
ATOM    127  N   ???     1       5.885   4.795  -7.592
ATOM    128  H   ???     1       6.857   5.164  -7.556
ATOM    129  H   ???     1       5.348   5.020  -8.435
ATOM    130  N   ???     1       3.923   4.104  -6.547
ATOM    131  C   ???     1       3.306   3.730  -5.392
ATOM    132  N   ???     1       1.970   3.400  -5.513
ATOM    133  H   ???     1       1.526   3.552  -6.419
ATOM    134  C   ???     1       1.203   2.875  -4.428
ATOM    135  H   ???     1       0.484   2.130  -4.823
ATOM    136  O   ???     1       0.462   3.938  -3.802
ATOM    137  C   ???     1      -0.517   3.432  -2.881
ATOM    138  H   ???     1      -1.360   3.005  -3.475
ATOM    139  C   ???     1      -1.030   4.624  -2.089
ATOM    140  H   ???     1      -0.218   5.035  -1.471
ATOM    141  H   ???     1      -1.861   4.312  -1.436
ATOM    142  O   ???     1      -1.484   5.666  -2.994
ATOM    143  P   ???     1      -1.413   7.188  -2.365
ATOM    144  O   ???     1      -1.073   8.098  -3.550
ATOM    145  O   ???     1      -0.422   7.297  -1.244
ATOM    146  O   ???     1      -2.924   7.481  -1.834
ATOM    147  H   ???     1      -2.945   7.697  -0.863
ATOM    148 MO   ???     1       0.163  -0.154   0.415
ATOM    149  O   ???     1      -0.609  -1.543  -0.302
ATOM    150  O   ???     1       1.700  -0.625   1.534
ATOM    151  O   ???     1       6.445  -2.342   1.378
ATOM    152  H   ???     1       6.225  -1.568   0.682
ATOM    153  H   ???     1       6.231  -3.250   1.045
ATOM    154  O   ???     1       6.027  -2.247   3.895
ATOM    155  H   ???     1       5.063  -2.130   4.087
ATOM    156  H   ???     1       6.088  -2.248   2.860
ATOM    157  O   ???     1       7.649  -0.273   4.659
ATOM    158  H   ???     1       7.096  -1.065   4.360
ATOM    159  H   ???     1       7.566  -0.308   5.665
ATOM    160  O   ???     1       9.133  -2.067   1.606
ATOM    161  H   ???     1       9.437  -3.001   1.483
ATOM    162  H   ???     1       8.136  -2.166   1.644
ATOM    163  O   ???     1       7.524  -4.522   3.753
ATOM    164  H   ???     1       6.970  -3.734   4.017
ATOM    165  H   ???     1       7.057  -4.820   2.925
END
MPH, state Im
ATOM      1  H   ???     1       2.387   3.211   1.499
ATOM      2  C   ???     1       3.324   2.932   0.971
ATOM      3  H   ???     1       3.106   2.698  -0.085
ATOM      4  H   ???     1       4.057   3.749   1.028
ATOM      5  N   ???     1       3.869   1.744   1.626
ATOM      6  H   ???     1       3.262   0.913   1.609
ATOM      7  C   ???     1       4.977   1.685   2.381
ATOM      8  N   ???     1       5.782   2.761   2.525
ATOM      9  H   ???     1       5.385   3.708   2.475
ATOM     10  H   ???     1       6.619   2.667   3.119
ATOM     11  N   ???     1       5.312   0.527   2.970
```

ATOM	12	H	???	1	4.711	-0.302	2.876
ATOM	13	H	???	1	6.118	0.469	3.613
ATOM	14	H	???	1	8.171	2.425	-1.060
ATOM	15	C	???	1	7.434	1.637	-0.861
ATOM	16	N	???	1	6.144	1.641	-1.351
ATOM	17	H	???	1	5.752	2.389	-1.960
ATOM	18	C	???	1	5.530	0.511	-0.908
ATOM	19	H	???	1	4.487	0.266	-1.127
ATOM	20	N	???	1	6.359	-0.213	-0.153
ATOM	21	C	???	1	7.552	0.480	-0.113
ATOM	22	H	???	1	8.414	0.088	0.434
ATOM	23	H	???	1	-3.291	0.195	1.476
ATOM	24	C	???	1	-2.715	0.804	2.213
ATOM	25	H	???	1	-3.263	0.838	3.172
ATOM	26	H	???	1	-2.643	1.820	1.791
ATOM	27	S	???	1	-1.034	0.117	2.515
ATOM	28	H	???	1	-2.842	-2.704	1.487
ATOM	29	C	???	1	-2.841	-3.751	1.904
ATOM	30	H	???	1	-3.774	-4.239	1.578
ATOM	31	H	???	1	-1.968	-4.286	1.480
ATOM	32	C	???	1	-2.752	-3.795	3.443
ATOM	33	O	???	1	-3.313	-4.724	4.046
ATOM	34	N	???	1	-2.001	-2.837	4.005
ATOM	35	H	???	1	-1.596	-2.092	3.413
ATOM	36	C	???	1	-1.801	-2.682	5.442
ATOM	37	H	???	1	-1.781	-3.684	5.908
ATOM	38	H	???	1	-0.818	-2.198	5.559
ATOM	39	H	???	1	-2.549	-2.075	5.972
ATOM	40	H	???	1	0.487	-3.537	10.188
ATOM	41	C	???	1	1.436	-3.379	9.618
ATOM	42	H	???	1	1.705	-2.318	9.673
ATOM	43	H	???	1	2.236	-3.949	10.126
ATOM	44	N	???	1	1.360	-3.832	8.227
ATOM	45	H	???	1	1.382	-4.849	8.076
ATOM	46	C	???	1	1.357	-3.100	7.099
ATOM	47	N	???	1	1.405	-1.765	7.071
ATOM	48	H	???	1	1.249	-1.191	7.912
ATOM	49	H	???	1	1.428	-1.270	6.157
ATOM	50	N	???	1	1.430	-3.775	5.912
ATOM	51	H	???	1	1.192	-4.771	5.952
ATOM	52	H	???	1	1.146	-3.294	5.022
ATOM	53	H	???	1	3.315	1.102	8.351
ATOM	54	C	???	1	2.962	1.683	7.460
ATOM	55	H	???	1	2.090	1.150	7.036
ATOM	56	H	???	1	2.594	2.669	7.806
ATOM	57	C	???	1	4.023	1.893	6.357
ATOM	58	H	???	1	4.408	0.912	5.998
ATOM	59	C	???	1	3.317	2.541	5.158
ATOM	60	H	???	1	2.728	3.419	5.482
ATOM	61	H	???	1	4.053	2.872	4.409
ATOM	62	H	???	1	2.625	1.826	4.679
ATOM	63	C	???	1	5.235	2.727	6.813
ATOM	64	H	???	1	5.806	2.225	7.621
ATOM	65	H	???	1	5.931	2.882	5.962
ATOM	66	H	???	1	4.911	3.718	7.184
ATOM	67	H	???	1	-2.062	1.511	8.433
ATOM	68	C	???	1	-1.386	1.728	7.596
ATOM	69	C	???	1	-0.798	0.782	6.778
ATOM	70	H	???	1	-0.801	-0.301	6.858
ATOM	71	N	???	1	-0.181	1.395	5.708
ATOM	72	H	???	1	0.274	0.890	4.938
ATOM	73	C	???	1	-0.376	2.755	5.794
ATOM	74	C	???	1	-0.018	3.782	4.907
ATOM	75	H	???	1	0.506	3.549	3.978
ATOM	76	C	???	1	-0.386	5.090	5.235
ATOM	77	H	???	1	-0.129	5.913	4.563
ATOM	78	C	???	1	-1.123	5.355	6.416
ATOM	79	H	???	1	-1.426	6.379	6.640
ATOM	80	C	???	1	-1.504	4.334	7.287
ATOM	81	H	???	1	-2.117	4.553	8.165
ATOM	82	C	???	1	-1.123	3.007	6.993
ATOM	83	H	???	1	1.854	-6.252	-1.688
ATOM	84	C	???	1	2.202	-5.268	-1.326
ATOM	85	C	???	1	1.644	-4.735	-0.153
ATOM	86	H	???	1	0.826	-5.258	0.362
ATOM	87	C	???	1	2.115	-3.543	0.402
ATOM	88	H	???	1	1.660	-3.157	1.317
ATOM	89	C	???	1	3.174	-2.853	-0.225
ATOM	90	O	???	1	3.679	-1.701	0.265

```
ATOM     91  H   ???     1       3.054  -1.337   0.959
ATOM     92  C   ???     1       3.738  -3.375  -1.406
ATOM     93  H   ???     1       4.535  -2.804  -1.895
ATOM     94  C   ???     1       3.256  -4.573  -1.946
ATOM     95  H   ???     1       3.705  -4.951  -2.866
ATOM     96  H   ???     1       5.092  -8.254   5.430
ATOM     97  C   ???     1       5.601  -7.256   5.344
ATOM     98  H   ???     1       5.523  -6.739   6.311
ATOM     99  H   ???     1       6.673  -7.416   5.127
ATOM    100  N   ???     1       5.029  -6.449   4.269
ATOM    101  H   ???     1       5.238  -6.723   3.295
ATOM    102  C   ???     1       4.385  -5.272   4.379
ATOM    103  N   ???     1       4.194  -4.659   5.569
ATOM    104  H   ???     1       4.424  -5.141   6.448
ATOM    105  H   ???     1       3.358  -4.057   5.623
ATOM    106  N   ???     1       3.891  -4.701   3.266
ATOM    107  H   ???     1       4.130  -5.151   2.369
ATOM    108  H   ???     1       3.621  -3.697   3.252
ATOM    109  S   ???     1       1.934  -1.365   3.316
ATOM    110  O   ???     1       1.005  -2.554   3.376
ATOM    111  O   ???     1       1.683  -0.399   4.445
ATOM    112  O   ???     1       3.393  -1.836   3.336
ATOM    113  S   ???     1      -0.922   1.753  -0.542
ATOM    114  C   ???     1       0.095   2.331  -1.917
ATOM    115  C   ???     1       1.321   1.784  -2.125
ATOM    116  S   ???     1       1.932   0.487  -1.053
ATOM    117  C   ???     1       2.176   2.186  -3.317
ATOM    118  H   ???     1       2.651   1.283  -3.750
ATOM    119  N   ???     1       3.276   3.102  -2.970
ATOM    120  H   ???     1       2.946   3.858  -2.351
ATOM    121  C   ???     1       3.955   3.598  -4.129
ATOM    122  C   ???     1       5.343   3.949  -4.055
ATOM    123  O   ???     1       6.056   3.909  -3.035
ATOM    124  N   ???     1       5.902   4.395  -5.279
ATOM    125  H   ???     1       6.896   4.701  -5.271
ATOM    126  C   ???     1       5.208   4.434  -6.460
ATOM    127  N   ???     1       5.873   4.802  -7.581
ATOM    128  H   ???     1       6.844   5.173  -7.549
ATOM    129  H   ???     1       5.336   5.017  -8.427
ATOM    130  N   ???     1       3.921   4.084  -6.531
ATOM    131  C   ???     1       3.319   3.686  -5.373
ATOM    132  N   ???     1       1.998   3.302  -5.492
ATOM    133  H   ???     1       1.534   3.477  -6.383
ATOM    134  C   ???     1       1.240   2.800  -4.388
ATOM    135  H   ???     1       0.526   2.040  -4.763
ATOM    136  O   ???     1       0.496   3.877  -3.792
ATOM    137  C   ???     1      -0.476   3.400  -2.847
ATOM    138  H   ???     1      -1.339   2.979  -3.418
ATOM    139  C   ???     1      -0.950   4.617  -2.069
ATOM    140  H   ???     1      -0.107   5.042  -1.504
ATOM    141  H   ???     1      -1.752   4.329  -1.368
ATOM    142  O   ???     1      -1.437   5.642  -2.981
ATOM    143  P   ???     1      -1.409   7.168  -2.357
ATOM    144  O   ???     1      -1.096   8.087  -3.542
ATOM    145  O   ???     1      -0.413   7.312  -1.243
ATOM    146  O   ???     1      -2.926   7.410  -1.815
ATOM    147  H   ???     1      -2.942   7.652  -0.851
ATOM    148 MO   ???     1       0.124  -0.176   0.412
ATOM    149  O   ???     1      -0.665  -1.531  -0.336
ATOM    150  O   ???     1       1.801  -0.649   1.893
ATOM    151  O   ???     1       6.446  -2.338   1.375
ATOM    152  H   ???     1       6.228  -1.557   0.680
ATOM    153  H   ???     1       6.231  -3.245   1.042
ATOM    154  O   ???     1       6.079  -2.257   3.896
ATOM    155  H   ???     1       5.120  -2.151   4.108
ATOM    156  H   ???     1       6.121  -2.253   2.858
ATOM    157  O   ???     1       7.659  -0.266   4.663
ATOM    158  H   ???     1       7.119  -1.065   4.360
ATOM    159  H   ???     1       7.575  -0.303   5.669
ATOM    160  O   ???     1       9.133  -2.070   1.608
ATOM    161  H   ???     1       9.437  -3.003   1.485
ATOM    162  H   ???     1       8.136  -2.167   1.638
ATOM    163  O   ???     1       7.537  -4.548   3.755
ATOM    164  H   ???     1       6.996  -3.749   4.014
ATOM    165  H   ???     1       7.065  -4.843   2.929
END
MPH, state Ts2
ATOM      1  H   ???     1       2.391   3.206   1.504
ATOM      2  C   ???     1       3.334   2.921   0.989
```

ATOM	3	H	???	1	3.119	2.647	-0.060
ATOM	4	H	???	1	4.050	3.755	1.011
ATOM	5	N	???	1	3.906	1.775	1.692
ATOM	6	H	???	1	3.305	0.947	1.822
ATOM	7	C	???	1	5.044	1.756	2.405
ATOM	8	N	???	1	5.838	2.842	2.501
ATOM	9	H	???	1	5.418	3.780	2.472
ATOM	10	H	???	1	6.686	2.761	3.082
ATOM	11	N	???	1	5.413	0.608	2.991
ATOM	12	H	???	1	4.796	-0.201	2.889
ATOM	13	H	???	1	6.209	0.548	3.649
ATOM	14	H	???	1	8.169	2.425	-1.062
ATOM	15	C	???	1	7.427	1.639	-0.867
ATOM	16	N	???	1	6.136	1.657	-1.354
ATOM	17	H	???	1	5.751	2.409	-1.962
ATOM	18	C	???	1	5.515	0.528	-0.918
ATOM	19	H	???	1	4.473	0.287	-1.138
ATOM	20	N	???	1	6.340	-0.210	-0.173
ATOM	21	C	???	1	7.538	0.476	-0.129
ATOM	22	H	???	1	8.397	0.075	0.415
ATOM	23	H	???	1	-3.276	0.190	1.471
ATOM	24	C	???	1	-2.667	0.785	2.192
ATOM	25	H	???	1	-3.133	0.780	3.194
ATOM	26	H	???	1	-2.608	1.815	1.804
ATOM	27	S	???	1	-0.963	0.057	2.321
ATOM	28	H	???	1	-2.842	-2.705	1.487
ATOM	29	C	???	1	-2.838	-3.751	1.904
ATOM	30	H	???	1	-3.772	-4.238	1.578
ATOM	31	H	???	1	-1.966	-4.286	1.479
ATOM	32	C	???	1	-2.750	-3.793	3.444
ATOM	33	O	???	1	-3.323	-4.713	4.046
ATOM	34	N	???	1	-1.988	-2.842	4.006
ATOM	35	H	???	1	-1.564	-2.118	3.407
ATOM	36	C	???	1	-1.792	-2.682	5.446
ATOM	37	H	???	1	-1.777	-3.684	5.911
ATOM	38	H	???	1	-0.804	-2.211	5.568
ATOM	39	H	???	1	-2.545	-2.074	5.973
ATOM	40	H	???	1	0.495	-3.542	10.218
ATOM	41	C	???	1	1.465	-3.395	9.699
ATOM	42	H	???	1	1.726	-2.330	9.723
ATOM	43	H	???	1	2.243	-3.931	10.276
ATOM	44	N	???	1	1.454	-3.909	8.340
ATOM	45	H	???	1	1.472	-4.927	8.220
ATOM	46	C	???	1	1.371	-3.205	7.193
ATOM	47	N	???	1	1.338	-1.870	7.175
ATOM	48	H	???	1	1.191	-1.313	8.027
ATOM	49	H	???	1	1.412	-1.346	6.277
ATOM	50	N	???	1	1.390	-3.899	6.039
ATOM	51	H	???	1	1.480	-4.916	6.092
ATOM	52	H	???	1	1.251	-3.448	5.112
ATOM	53	H	???	1	3.316	1.100	8.350
ATOM	54	C	???	1	2.964	1.677	7.457
ATOM	55	H	???	1	2.102	1.131	7.031
ATOM	56	H	???	1	2.590	2.663	7.797
ATOM	57	C	???	1	4.029	1.884	6.357
ATOM	58	H	???	1	4.405	0.901	5.997
ATOM	59	C	???	1	3.330	2.539	5.157
ATOM	60	H	???	1	2.739	3.415	5.480
ATOM	61	H	???	1	4.070	2.873	4.414
ATOM	62	H	???	1	2.643	1.823	4.673
ATOM	63	C	???	1	5.245	2.712	6.815
ATOM	64	H	???	1	5.812	2.206	7.622
ATOM	65	H	???	1	5.942	2.865	5.965
ATOM	66	H	???	1	4.925	3.705	7.188
ATOM	67	H	???	1	-2.063	1.510	8.435
ATOM	68	C	???	1	-1.387	1.726	7.599
ATOM	69	C	???	1	-0.783	0.774	6.801
ATOM	70	H	???	1	-0.762	-0.307	6.905
ATOM	71	N	???	1	-0.175	1.378	5.721
ATOM	72	H	???	1	0.379	0.867	5.023
ATOM	73	C	???	1	-0.372	2.739	5.795
ATOM	74	C	???	1	-0.007	3.761	4.904
ATOM	75	H	???	1	0.530	3.522	3.984
ATOM	76	C	???	1	-0.380	5.070	5.219
ATOM	77	H	???	1	-0.113	5.890	4.544
ATOM	78	C	???	1	-1.128	5.344	6.390
ATOM	79	H	???	1	-1.433	6.370	6.604
ATOM	80	C	???	1	-1.516	4.328	7.265
ATOM	81	H	???	1	-2.136	4.553	8.138

ATOM	82	C	???	1	-1.130	2.999	6.985
ATOM	83	H	???	1	1.850	-6.244	-1.681
ATOM	84	C	???	1	2.187	-5.252	-1.312
ATOM	85	C	???	1	1.677	-4.740	-0.104
ATOM	86	H	???	1	0.898	-5.286	0.448
ATOM	87	C	???	1	2.152	-3.544	0.448
ATOM	88	H	???	1	1.761	-3.180	1.408
ATOM	89	C	???	1	3.170	-2.830	-0.221
ATOM	90	O	???	1	3.692	-1.684	0.256
ATOM	91	H	???	1	3.287	-1.427	1.146
ATOM	92	C	???	1	3.674	-3.322	-1.444
ATOM	93	H	???	1	4.440	-2.734	-1.962
ATOM	94	C	???	1	3.191	-4.523	-1.976
ATOM	95	H	???	1	3.606	-4.883	-2.922
ATOM	96	H	???	1	5.091	-8.260	5.434
ATOM	97	C	???	1	5.598	-7.267	5.352
ATOM	98	H	???	1	5.517	-6.749	6.318
ATOM	99	H	???	1	6.672	-7.421	5.131
ATOM	100	N	???	1	4.997	-6.477	4.285
ATOM	101	H	???	1	5.154	-6.780	3.309
ATOM	102	C	???	1	4.477	-5.240	4.387
ATOM	103	N	???	1	4.319	-4.601	5.558
ATOM	104	H	???	1	4.442	-5.057	6.469
ATOM	105	H	???	1	3.851	-3.683	5.509
ATOM	106	N	???	1	4.059	-4.632	3.264
ATOM	107	H	???	1	4.306	-5.058	2.360
ATOM	108	H	???	1	3.758	-3.650	3.337
ATOM	109	S	???	1	2.304	-1.447	3.794
ATOM	110	O	???	1	1.331	-2.565	3.487
ATOM	111	O	???	1	1.731	-0.461	4.781
ATOM	112	O	???	1	3.578	-2.075	4.408
ATOM	113	S	???	1	-1.016	1.780	-0.689
ATOM	114	C	???	1	0.020	2.388	-2.038
ATOM	115	C	???	1	1.250	1.852	-2.226
ATOM	116	S	???	1	1.820	0.508	-1.181
ATOM	117	C	???	1	2.119	2.268	-3.396
ATOM	118	H	???	1	2.581	1.368	-3.848
ATOM	119	N	???	1	3.230	3.153	-3.015
ATOM	120	H	???	1	2.914	3.904	-2.383
ATOM	121	C	???	1	3.935	3.643	-4.160
ATOM	122	C	???	1	5.331	3.965	-4.073
ATOM	123	O	???	1	6.033	3.916	-3.046
ATOM	124	N	???	1	5.907	4.399	-5.293
ATOM	125	H	???	1	6.903	4.699	-5.277
ATOM	126	C	???	1	5.227	4.442	-6.482
ATOM	127	N	???	1	5.898	4.792	-7.603
ATOM	128	H	???	1	6.870	5.161	-7.568
ATOM	129	H	???	1	5.361	5.018	-8.446
ATOM	130	N	???	1	3.935	4.111	-6.562
ATOM	131	C	???	1	3.312	3.748	-5.408
ATOM	132	N	???	1	1.974	3.431	-5.539
ATOM	133	H	???	1	1.539	3.580	-6.450
ATOM	134	C	???	1	1.197	2.915	-4.460
ATOM	135	H	???	1	0.479	2.170	-4.856
ATOM	136	O	???	1	0.449	3.983	-3.845
ATOM	137	C	???	1	-0.546	3.464	-2.953
ATOM	138	H	???	1	-1.373	3.036	-3.568
ATOM	139	C	???	1	-1.088	4.626	-2.139
ATOM	140	H	???	1	-0.299	5.021	-1.480
ATOM	141	H	???	1	-1.936	4.282	-1.522
ATOM	142	O	???	1	-1.529	5.688	-3.020
ATOM	143	P	???	1	-1.419	7.201	-2.375
ATOM	144	O	???	1	-1.055	8.109	-3.554
ATOM	145	O	???	1	-0.433	7.263	-1.245
ATOM	146	O	???	1	-2.923	7.535	-1.851
ATOM	147	H	???	1	-2.949	7.726	-0.875
ATOM	148	MO	???	1	-0.059	-0.192	0.108
ATOM	149	O	???	1	-0.932	-1.478	-0.668
ATOM	150	O	???	1	2.716	-0.722	2.496
ATOM	151	O	???	1	6.407	-2.348	1.359
ATOM	152	H	???	1	6.201	-1.584	0.655
ATOM	153	H	???	1	6.232	-3.267	1.035
ATOM	154	O	???	1	6.196	-2.229	3.910
ATOM	155	H	???	1	5.244	-2.109	4.199
ATOM	156	H	???	1	6.179	-2.238	2.883
ATOM	157	O	???	1	7.694	-0.234	4.675
ATOM	158	H	???	1	7.172	-1.047	4.356
ATOM	159	H	???	1	7.597	-0.281	5.680
ATOM	160	O	???	1	9.110	-2.074	1.603

```
ATOM    161  H   ???     1       9.402  -3.014   1.497
ATOM    162  H   ???     1       8.112  -2.159   1.634
ATOM    163  O   ???     1       7.578  -4.551   3.776
ATOM    164  H   ???     1       7.060  -3.728   4.018
ATOM    165  H   ???     1       7.080  -4.868   2.977
END
MPH, state P
ATOM      1  H   ???     1       2.393   3.206   1.505
ATOM      2  C   ???     1       3.340   2.922   0.992
ATOM      3  H   ???     1       3.127   2.649  -0.057
ATOM      4  H   ???     1       4.051   3.759   1.016
ATOM      5  N   ???     1       3.919   1.780   1.694
ATOM      6  H   ???     1       3.330   0.944   1.814
ATOM      7  C   ???     1       5.061   1.763   2.405
ATOM      8  N   ???     1       5.851   2.853   2.494
ATOM      9  H   ???     1       5.429   3.790   2.458
ATOM     10  H   ???     1       6.696   2.777   3.079
ATOM     11  N   ???     1       5.435   0.619   2.990
ATOM     12  H   ???     1       4.795  -0.178   2.924
ATOM     13  H   ???     1       6.229   0.564   3.652
ATOM     14  H   ???     1       8.169   2.425  -1.062
ATOM     15  C   ???     1       7.427   1.639  -0.868
ATOM     16  N   ???     1       6.136   1.660  -1.356
ATOM     17  H   ???     1       5.753   2.412  -1.961
ATOM     18  C   ???     1       5.515   0.529  -0.922
ATOM     19  H   ???     1       4.472   0.292  -1.143
ATOM     20  N   ???     1       6.338  -0.211  -0.179
ATOM     21  C   ???     1       7.535   0.473  -0.132
ATOM     22  H   ???     1       8.394   0.070   0.412
ATOM     23  H   ???     1      -3.274   0.190   1.471
ATOM     24  C   ???     1      -2.663   0.783   2.192
ATOM     25  H   ???     1      -3.132   0.783   3.192
ATOM     26  H   ???     1      -2.601   1.812   1.801
ATOM     27  S   ???     1      -0.957   0.058   2.334
ATOM     28  H   ???     1      -2.843  -2.704   1.485
ATOM     29  C   ???     1      -2.841  -3.752   1.901
ATOM     30  H   ???     1      -3.773  -4.238   1.571
ATOM     31  H   ???     1      -1.967  -4.286   1.478
ATOM     32  C   ???     1      -2.754  -3.796   3.440
ATOM     33  O   ???     1      -3.321  -4.722   4.038
ATOM     34  N   ???     1      -1.995  -2.844   4.006
ATOM     35  H   ???     1      -1.603  -2.097   3.413
ATOM     36  C   ???     1      -1.801  -2.692   5.448
ATOM     37  H   ???     1      -1.797  -3.696   5.906
ATOM     38  H   ???     1      -0.807  -2.235   5.574
ATOM     39  H   ???     1      -2.548  -2.079   5.974
ATOM     40  H   ???     1       0.498  -3.540  10.231
ATOM     41  C   ???     1       1.478  -3.388   9.737
ATOM     42  H   ???     1       1.730  -2.321   9.757
ATOM     43  H   ???     1       2.247  -3.908  10.339
ATOM     44  N   ???     1       1.511  -3.917   8.388
ATOM     45  H   ???     1       1.549  -4.936   8.280
ATOM     46  C   ???     1       1.347  -3.236   7.236
ATOM     47  N   ???     1       1.281  -1.903   7.198
ATOM     48  H   ???     1       1.187  -1.331   8.047
ATOM     49  H   ???     1       1.281  -1.404   6.285
ATOM     50  N   ???     1       1.291  -3.959   6.103
ATOM     51  H   ???     1       1.499  -4.958   6.178
ATOM     52  H   ???     1       1.332  -3.524   5.160
ATOM     53  H   ???     1       3.314   1.098   8.348
ATOM     54  C   ???     1       2.960   1.671   7.454
ATOM     55  H   ???     1       2.100   1.115   7.033
ATOM     56  H   ???     1       2.578   2.656   7.790
ATOM     57  C   ???     1       4.021   1.878   6.350
ATOM     58  H   ???     1       4.411   0.897   6.004
ATOM     59  C   ???     1       3.311   2.507   5.142
ATOM     60  H   ???     1       2.709   3.381   5.450
ATOM     61  H   ???     1       4.044   2.840   4.390
ATOM     62  H   ???     1       2.631   1.777   4.666
ATOM     63  C   ???     1       5.225   2.730   6.796
ATOM     64  H   ???     1       5.797   2.242   7.611
ATOM     65  H   ???     1       5.921   2.880   5.945
ATOM     66  H   ???     1       4.893   3.723   7.156
ATOM     67  H   ???     1      -2.063   1.511   8.434
ATOM     68  C   ???     1      -1.388   1.727   7.597
ATOM     69  C   ???     1      -0.790   0.776   6.793
ATOM     70  H   ???     1      -0.774  -0.305   6.894
ATOM     71  N   ???     1      -0.183   1.382   5.713
ATOM     72  H   ???     1       0.340   0.868   4.996
```

ATOM	73	C	???	1	-0.379	2.743	5.790
ATOM	74	C	???	1	-0.016	3.767	4.901
ATOM	75	H	???	1	0.517	3.530	3.977
ATOM	76	C	???	1	-0.385	5.076	5.221
ATOM	77	H	???	1	-0.121	5.897	4.547
ATOM	78	C	???	1	-1.128	5.348	6.396
ATOM	79	H	???	1	-1.430	6.374	6.614
ATOM	80	C	???	1	-1.514	4.330	7.270
ATOM	81	H	???	1	-2.129	4.554	8.146
ATOM	82	C	???	1	-1.131	3.002	6.985
ATOM	83	H	???	1	1.848	-6.243	-1.681
ATOM	84	C	???	1	2.183	-5.250	-1.311
ATOM	85	C	???	1	1.653	-4.735	-0.114
ATOM	86	H	???	1	0.867	-5.281	0.426
ATOM	87	C	???	1	2.120	-3.537	0.442
ATOM	88	H	???	1	1.724	-3.170	1.398
ATOM	89	C	???	1	3.152	-2.826	-0.208
ATOM	90	O	???	1	3.669	-1.681	0.278
ATOM	91	H	???	1	3.322	-1.466	1.202
ATOM	92	C	???	1	3.671	-3.319	-1.425
ATOM	93	H	???	1	4.449	-2.735	-1.930
ATOM	94	C	???	1	3.196	-4.520	-1.962
ATOM	95	H	???	1	3.626	-4.883	-2.900
ATOM	96	H	???	1	5.090	-8.251	5.431
ATOM	97	C	???	1	5.591	-7.247	5.344
ATOM	98	H	???	1	5.517	-6.729	6.311
ATOM	99	H	???	1	6.662	-7.394	5.109
ATOM	100	N	???	1	4.970	-6.459	4.283
ATOM	101	H	???	1	5.113	-6.770	3.307
ATOM	102	C	???	1	4.411	-5.235	4.375
ATOM	103	N	???	1	4.198	-4.609	5.544
ATOM	104	H	???	1	4.371	-5.043	6.459
ATOM	105	H	???	1	3.892	-3.624	5.497
ATOM	106	N	???	1	4.031	-4.623	3.239
ATOM	107	H	???	1	4.238	-5.109	2.354
ATOM	108	H	???	1	3.276	-3.907	3.269
ATOM	109	S	???	1	2.457	-1.492	3.919
ATOM	110	O	???	1	1.838	-2.849	3.556
ATOM	111	O	???	1	1.473	-0.658	4.694
ATOM	112	O	???	1	3.695	-1.759	4.786
ATOM	113	S	???	1	-1.001	1.770	-0.676
ATOM	114	C	???	1	0.028	2.381	-2.028
ATOM	115	C	???	1	1.260	1.850	-2.217
ATOM	116	S	???	1	1.840	0.511	-1.169
ATOM	117	C	???	1	2.126	2.266	-3.389
ATOM	118	H	???	1	2.590	1.366	-3.841
ATOM	119	N	???	1	3.235	3.154	-3.011
ATOM	120	H	???	1	2.919	3.906	-2.380
ATOM	121	C	???	1	3.938	3.644	-4.158
ATOM	122	C	???	1	5.333	3.968	-4.073
ATOM	123	O	???	1	6.035	3.922	-3.046
ATOM	124	N	???	1	5.907	4.400	-5.294
ATOM	125	H	???	1	6.904	4.701	-5.279
ATOM	126	C	???	1	5.226	4.442	-6.482
ATOM	127	N	???	1	5.896	4.792	-7.603
ATOM	128	H	???	1	6.868	5.161	-7.569
ATOM	129	H	???	1	5.359	5.017	-8.446
ATOM	130	N	???	1	3.934	4.110	-6.560
ATOM	131	C	???	1	3.313	3.747	-5.405
ATOM	132	N	???	1	1.975	3.428	-5.533
ATOM	133	H	???	1	1.538	3.575	-6.444
ATOM	134	C	???	1	1.202	2.910	-4.454
ATOM	135	H	???	1	0.483	2.164	-4.848
ATOM	136	O	???	1	0.453	3.977	-3.836
ATOM	137	C	???	1	-0.541	3.455	-2.944
ATOM	138	H	???	1	-1.367	3.024	-3.560
ATOM	139	C	???	1	-1.089	4.619	-2.134
ATOM	140	H	???	1	-0.305	5.015	-1.471
ATOM	141	H	???	1	-1.941	4.276	-1.522
ATOM	142	O	???	1	-1.525	5.680	-3.019
ATOM	143	P	???	1	-1.419	7.195	-2.373
ATOM	144	O	???	1	-1.059	8.105	-3.551
ATOM	145	O	???	1	-0.431	7.260	-1.245
ATOM	146	O	???	1	-2.924	7.520	-1.846
ATOM	147	H	???	1	-2.947	7.720	-0.871
ATOM	148	MO	???	1	-0.027	-0.192	0.131
ATOM	149	O	???	1	-0.896	-1.483	-0.643
ATOM	150	O	???	1	2.857	-0.775	2.614
ATOM	151	O	???	1	6.396	-2.354	1.372

```
ATOM    152  H   ???     1       6.189  -1.602   0.666
ATOM    153  H   ???     1       6.220  -3.276   1.055
ATOM    154  O   ???     1       6.189  -2.252   3.928
ATOM    155  H   ???     1       5.252  -2.115   4.261
ATOM    156  H   ???     1       6.144  -2.258   2.904
ATOM    157  O   ???     1       7.688  -0.242   4.671
ATOM    158  H   ???     1       7.167  -1.058   4.354
ATOM    159  H   ???     1       7.590  -0.287   5.675
ATOM    160  O   ???     1       9.100  -2.077   1.606
ATOM    161  H   ???     1       9.388  -3.018   1.501
ATOM    162  H   ???     1       8.103  -2.159   1.646
ATOM    163  O   ???     1       7.572  -4.563   3.774
ATOM    164  H   ???     1       7.053  -3.739   4.016
ATOM    165  H   ???     1       7.073  -4.878   2.974
END
MPO, state R
ATOM      1  H   ???     1       2.387   3.209   1.495
ATOM      2  C   ???     1       3.323   2.930   0.961
ATOM      3  H   ???     1       3.106   2.732  -0.103
ATOM      4  H   ???     1       4.056   3.745   1.035
ATOM      5  N   ???     1       3.873   1.727   1.582
ATOM      6  H   ???     1       3.324   0.867   1.471
ATOM      7  C   ???     1       4.965   1.664   2.368
ATOM      8  N   ???     1       5.779   2.735   2.513
ATOM      9  H   ???     1       5.402   3.686   2.426
ATOM     10  H   ???     1       6.587   2.646   3.146
ATOM     11  N   ???     1       5.272   0.511   2.973
ATOM     12  H   ???     1       4.604  -0.283   2.965
ATOM     13  H   ???     1       6.079   0.454   3.613
ATOM     14  H   ???     1       8.175   2.417  -1.055
ATOM     15  C   ???     1       7.448   1.617  -0.846
ATOM     16  N   ???     1       6.161   1.573  -1.341
ATOM     17  H   ???     1       5.751   2.283  -1.976
ATOM     18  C   ???     1       5.583   0.429  -0.881
ATOM     19  H   ???     1       4.556   0.145  -1.112
ATOM     20  N   ???     1       6.428  -0.258  -0.113
ATOM     21  C   ???     1       7.597   0.475  -0.079
ATOM     22  H   ???     1       8.469   0.116   0.475
ATOM     23  H   ???     1      -3.281   0.199   1.464
ATOM     24  C   ???     1      -2.686   0.815   2.175
ATOM     25  H   ???     1      -3.138   0.775   3.182
ATOM     26  H   ???     1      -2.718   1.851   1.802
ATOM     27  S   ???     1      -0.935   0.274   2.347
ATOM     28  H   ???     1      -2.836  -2.702   1.493
ATOM     29  C   ???     1      -2.833  -3.751   1.908
ATOM     30  H   ???     1      -3.770  -4.233   1.582
ATOM     31  H   ???     1      -1.964  -4.286   1.481
ATOM     32  C   ???     1      -2.750  -3.793   3.448
ATOM     33  O   ???     1      -3.352  -4.695   4.050
ATOM     34  N   ???     1      -1.981  -2.852   4.016
ATOM     35  H   ???     1      -1.444  -2.214   3.418
ATOM     36  C   ???     1      -1.795  -2.697   5.458
ATOM     37  H   ???     1      -1.801  -3.700   5.923
ATOM     38  H   ???     1      -0.799  -2.245   5.590
ATOM     39  H   ???     1      -2.546  -2.080   5.977
ATOM     40  H   ???     1       0.493  -3.545  10.224
ATOM     41  C   ???     1       1.463  -3.403   9.715
ATOM     42  H   ???     1       1.724  -2.337   9.733
ATOM     43  H   ???     1       2.238  -3.938  10.296
ATOM     44  N   ???     1       1.447  -3.921   8.359
ATOM     45  H   ???     1       1.488  -4.939   8.234
ATOM     46  C   ???     1       1.418  -3.208   7.220
ATOM     47  N   ???     1       1.388  -1.880   7.192
ATOM     48  H   ???     1       1.262  -1.314   8.040
ATOM     49  H   ???     1       1.554  -1.375   6.275
ATOM     50  N   ???     1       1.524  -3.891   6.048
ATOM     51  H   ???     1       1.370  -4.903   6.111
ATOM     52  H   ???     1       1.266  -3.413   5.130
ATOM     53  H   ???     1       3.321   1.102   8.351
ATOM     54  C   ???     1       2.979   1.684   7.460
ATOM     55  H   ???     1       2.127   1.134   7.020
ATOM     56  H   ???     1       2.595   2.666   7.801
ATOM     57  C   ???     1       4.060   1.902   6.376
ATOM     58  H   ???     1       4.459   0.924   6.028
ATOM     59  C   ???     1       3.370   2.536   5.161
ATOM     60  H   ???     1       2.765   3.409   5.465
ATOM     61  H   ???     1       4.115   2.869   4.421
ATOM     62  H   ???     1       2.699   1.804   4.677
ATOM     63  C   ???     1       5.256   2.751   6.848
```

ATOM	64	H	???	1	5.816	2.259	7.670
ATOM	65	H	???	1	5.966	2.905	6.009
ATOM	66	H	???	1	4.918	3.742	7.207
ATOM	67	H	???	1	-2.060	1.502	8.439
ATOM	68	C	???	1	-1.374	1.701	7.611
ATOM	69	C	???	1	-0.728	0.730	6.872
ATOM	70	H	???	1	-0.695	-0.347	7.019
ATOM	71	N	???	1	-0.091	1.301	5.790
ATOM	72	H	???	1	0.551	0.779	5.176
ATOM	73	C	???	1	-0.313	2.664	5.810
ATOM	74	C	???	1	0.052	3.660	4.892
ATOM	75	H	???	1	0.600	3.393	3.989
ATOM	76	C	???	1	-0.342	4.976	5.153
ATOM	77	H	???	1	-0.071	5.774	4.454
ATOM	78	C	???	1	-1.114	5.284	6.301
ATOM	79	H	???	1	-1.434	6.315	6.470
ATOM	80	C	???	1	-1.512	4.292	7.200
ATOM	81	H	???	1	-2.151	4.537	8.055
ATOM	82	C	???	1	-1.111	2.957	6.966
ATOM	83	H	???	1	1.851	-6.248	-1.677
ATOM	84	C	???	1	2.194	-5.264	-1.294
ATOM	85	C	???	1	1.696	-4.793	-0.064
ATOM	86	H	???	1	0.878	-5.323	0.447
ATOM	87	C	???	1	2.231	-3.667	0.567
ATOM	88	H	???	1	1.826	-3.326	1.526
ATOM	89	C	???	1	3.302	-2.975	-0.041
ATOM	90	O	???	1	3.908	-1.921	0.539
ATOM	91	H	???	1	3.595	-1.815	1.501
ATOM	92	C	???	1	3.765	-3.398	-1.307
ATOM	93	H	???	1	4.564	-2.821	-1.787
ATOM	94	C	???	1	3.229	-4.540	-1.915
ATOM	95	H	???	1	3.634	-4.864	-2.879
ATOM	96	H	???	1	5.095	-8.255	5.429
ATOM	97	C	???	1	5.608	-7.259	5.340
ATOM	98	H	???	1	5.537	-6.745	6.309
ATOM	99	H	???	1	6.679	-7.428	5.123
ATOM	100	N	???	1	5.039	-6.445	4.271
ATOM	101	H	???	1	5.231	-6.722	3.293
ATOM	102	C	???	1	4.415	-5.255	4.393
ATOM	103	N	???	1	4.255	-4.647	5.587
ATOM	104	H	???	1	4.491	-5.140	6.458
ATOM	105	H	???	1	3.414	-4.050	5.673
ATOM	106	N	???	1	3.919	-4.671	3.290
ATOM	107	H	???	1	4.125	-5.121	2.386
ATOM	108	H	???	1	3.618	-3.674	3.292
ATOM	109	S	???	1	1.873	-1.314	3.529
ATOM	110	O	???	1	1.101	-2.651	3.695
ATOM	111	O	???	1	1.977	-0.610	4.917
ATOM	112	O	???	1	3.395	-1.757	3.165
ATOM	113	S	???	1	-1.021	1.851	-0.761
ATOM	114	C	???	1	-0.026	2.395	-2.117
ATOM	115	C	???	1	1.245	1.908	-2.291
ATOM	116	S	???	1	1.899	0.643	-1.266
ATOM	117	C	???	1	2.071	2.486	-3.352
ATOM	118	N	???	1	3.347	2.724	-3.239
ATOM	119	C	???	1	3.991	3.362	-4.284
ATOM	120	C	???	1	5.369	3.788	-4.149
ATOM	121	O	???	1	6.067	3.730	-3.127
ATOM	122	N	???	1	5.917	4.352	-5.331
ATOM	123	H	???	1	6.905	4.679	-5.296
ATOM	124	C	???	1	5.239	4.439	-6.518
ATOM	125	N	???	1	5.903	4.818	-7.627
ATOM	126	H	???	1	6.883	5.168	-7.591
ATOM	127	H	???	1	5.366	5.063	-8.466
ATOM	128	N	???	1	3.941	4.132	-6.605
ATOM	129	C	???	1	3.335	3.665	-5.492
ATOM	130	N	???	1	1.986	3.421	-5.592
ATOM	131	H	???	1	1.530	3.621	-6.485
ATOM	132	C	???	1	1.197	2.942	-4.506
ATOM	133	H	???	1	0.528	2.131	-4.856
ATOM	134	O	???	1	0.340	4.001	-3.983
ATOM	135	C	???	1	-0.603	3.484	-3.026
ATOM	136	H	???	1	-1.474	3.068	-3.585
ATOM	137	C	???	1	-1.064	4.656	-2.178
ATOM	138	H	???	1	-0.216	5.034	-1.586
ATOM	139	H	???	1	-1.867	4.332	-1.494
ATOM	140	O	???	1	-1.553	5.716	-3.033
ATOM	141	P	???	1	-1.420	7.225	-2.383
ATOM	142	O	???	1	-1.035	8.125	-3.562

```
ATOM    143  O   ???     1       -0.447   7.264  -1.243
ATOM    144  O   ???     1       -2.922   7.594  -1.876
ATOM    145  H   ???     1       -2.956   7.755  -0.895
ATOM    146 MO   ???     1        0.110  -0.162   0.212
ATOM    147  O   ???     1       -0.792  -1.494  -0.497
ATOM    148  O   ???     1        1.532  -0.699   1.110
ATOM    149  O   ???     1        6.594  -2.419   1.424
ATOM    150  H   ???     1        6.330  -1.673   0.735
ATOM    151  H   ???     1        6.367  -3.340   1.125
ATOM    152  O   ???     1        5.994  -2.234   3.915
ATOM    153  H   ???     1        5.008  -2.097   4.002
ATOM    154  H   ???     1        6.145  -2.263   2.897
ATOM    155  O   ???     1        7.642  -0.275   4.661
ATOM    156  H   ???     1        7.065  -1.056   4.373
ATOM    157  H   ???     1        7.564  -0.306   5.668
ATOM    158  O   ???     1        9.234  -2.024   1.586
ATOM    159  H   ???     1        9.595  -2.931   1.437
ATOM    160  H   ???     1        8.247  -2.190   1.661
ATOM    161  O   ???     1        7.542  -4.490   3.800
ATOM    162  H   ???     1        6.961  -3.723   4.072
ATOM    163  H   ???     1        7.068  -4.818   2.993
END
MPO, state Ts1
ATOM      1  H   ???     1        2.388   3.217   1.498
ATOM      2  C   ???     1        3.327   2.951   0.968
ATOM      3  H   ???     1        3.120   2.753  -0.098
ATOM      4  H   ???     1        4.059   3.765   1.057
ATOM      5  N   ???     1        3.865   1.744   1.593
ATOM      6  H   ???     1        3.262   0.911   1.535
ATOM      7  C   ???     1        4.965   1.671   2.360
ATOM      8  N   ???     1        5.775   2.741   2.523
ATOM      9  H   ???     1        5.390   3.692   2.464
ATOM     10  H   ???     1        6.603   2.640   3.127
ATOM     11  N   ???     1        5.287   0.507   2.941
ATOM     12  H   ???     1        4.670  -0.312   2.851
ATOM     13  H   ???     1        6.089   0.441   3.586
ATOM     14  H   ???     1        8.175   2.419  -1.055
ATOM     15  C   ???     1        7.446   1.623  -0.847
ATOM     16  N   ???     1        6.161   1.592  -1.346
ATOM     17  H   ???     1        5.758   2.314  -1.976
ATOM     18  C   ???     1        5.567   0.456  -0.894
ATOM     19  H   ???     1        4.535   0.187  -1.125
ATOM     20  N   ???     1        6.404  -0.240  -0.122
ATOM     21  C   ???     1        7.582   0.479  -0.081
ATOM     22  H   ???     1        8.447   0.110   0.477
ATOM     23  H   ???     1       -3.287   0.197   1.473
ATOM     24  C   ???     1       -2.704   0.809   2.201
ATOM     25  H   ???     1       -3.230   0.826   3.173
ATOM     26  H   ???     1       -2.664   1.830   1.787
ATOM     27  S   ???     1       -0.996   0.184   2.489
ATOM     28  H   ???     1       -2.840  -2.703   1.488
ATOM     29  C   ???     1       -2.839  -3.751   1.904
ATOM     30  H   ???     1       -3.774  -4.236   1.578
ATOM     31  H   ???     1       -1.966  -4.286   1.480
ATOM     32  C   ???     1       -2.753  -3.793   3.444
ATOM     33  O   ???     1       -3.324  -4.715   4.047
ATOM     34  N   ???     1       -1.999  -2.838   4.008
ATOM     35  H   ???     1       -1.551  -2.127   3.412
ATOM     36  C   ???     1       -1.800  -2.683   5.445
ATOM     37  H   ???     1       -1.784  -3.686   5.911
ATOM     38  H   ???     1       -0.815  -2.204   5.564
ATOM     39  H   ???     1       -2.548  -2.075   5.973
ATOM     40  H   ???     1        0.489  -3.540  10.201
ATOM     41  C   ???     1        1.445  -3.388   9.652
ATOM     42  H   ???     1        1.712  -2.325   9.694
ATOM     43  H   ???     1        2.238  -3.947  10.184
ATOM     44  N   ???     1        1.388  -3.863   8.272
ATOM     45  H   ???     1        1.419  -4.880   8.130
ATOM     46  C   ???     1        1.365  -3.137   7.142
ATOM     47  N   ???     1        1.386  -1.804   7.115
ATOM     48  H   ???     1        1.240  -1.233   7.960
ATOM     49  H   ???     1        1.428  -1.303   6.198
ATOM     50  N   ???     1        1.441  -3.817   5.959
ATOM     51  H   ???     1        1.232  -4.819   6.012
ATOM     52  H   ???     1        1.137  -3.347   5.068
ATOM     53  H   ???     1        3.316   1.102   8.350
ATOM     54  C   ???     1        2.965   1.681   7.459
ATOM     55  H   ???     1        2.096   1.144   7.033
ATOM     56  H   ???     1        2.594   2.667   7.802
```

ATOM	57	C	???	1	4.029	1.890	6.357
ATOM	58	H	???	1	4.417	0.908	6.003
ATOM	59	C	???	1	3.325	2.531	5.153
ATOM	60	H	???	1	2.735	3.410	5.469
ATOM	61	H	???	1	4.062	2.857	4.403
ATOM	62	H	???	1	2.635	1.811	4.678
ATOM	63	C	???	1	5.238	2.729	6.813
ATOM	64	H	???	1	5.806	2.232	7.625
ATOM	65	H	???	1	5.936	2.881	5.964
ATOM	66	H	???	1	4.910	3.721	7.179
ATOM	67	H	???	1	-2.063	1.511	8.436
ATOM	68	C	???	1	-1.387	1.729	7.602
ATOM	69	C	???	1	-0.782	0.781	6.799
ATOM	70	H	???	1	-0.775	-0.301	6.890
ATOM	71	N	???	1	-0.163	1.391	5.730
ATOM	72	H	???	1	0.356	0.880	5.005
ATOM	73	C	???	1	-0.364	2.751	5.806
ATOM	74	C	???	1	-0.000	3.775	4.918
ATOM	75	H	???	1	0.535	3.541	3.996
ATOM	76	C	???	1	-0.378	5.083	5.236
ATOM	77	H	???	1	-0.116	5.905	4.563
ATOM	78	C	???	1	-1.129	5.352	6.407
ATOM	79	H	???	1	-1.438	6.376	6.623
ATOM	80	C	???	1	-1.517	4.332	7.278
ATOM	81	H	???	1	-2.140	4.553	8.149
ATOM	82	C	???	1	-1.127	3.006	6.995
ATOM	83	H	???	1	1.855	-6.253	-1.687
ATOM	84	C	???	1	2.205	-5.271	-1.324
ATOM	85	C	???	1	1.663	-4.740	-0.141
ATOM	86	H	???	1	0.848	-5.261	0.381
ATOM	87	C	???	1	2.145	-3.553	0.411
ATOM	88	H	???	1	1.702	-3.158	1.329
ATOM	89	C	???	1	3.198	-2.863	-0.228
ATOM	90	O	???	1	3.701	-1.710	0.255
ATOM	91	H	???	1	3.035	-1.323	0.911
ATOM	92	C	???	1	3.749	-3.386	-1.415
ATOM	93	H	???	1	4.541	-2.815	-1.913
ATOM	94	C	???	1	3.256	-4.580	-1.953
ATOM	95	H	???	1	3.691	-4.957	-2.879
ATOM	96	H	???	1	5.092	-8.254	5.429
ATOM	97	C	???	1	5.600	-7.254	5.341
ATOM	98	H	???	1	5.525	-6.738	6.309
ATOM	99	H	???	1	6.672	-7.415	5.123
ATOM	100	N	???	1	5.027	-6.447	4.268
ATOM	101	H	???	1	5.236	-6.721	3.292
ATOM	102	C	???	1	4.386	-5.269	4.379
ATOM	103	N	???	1	4.198	-4.658	5.569
ATOM	104	H	???	1	4.433	-5.140	6.447
ATOM	105	H	???	1	3.359	-4.059	5.632
ATOM	106	N	???	1	3.890	-4.696	3.267
ATOM	107	H	???	1	4.125	-5.146	2.368
ATOM	108	H	???	1	3.608	-3.697	3.254
ATOM	109	S	???	1	1.868	-1.364	3.403
ATOM	110	O	???	1	0.973	-2.597	3.469
ATOM	111	O	???	1	1.678	-0.476	4.639
ATOM	112	O	???	1	3.346	-1.805	3.312
ATOM	113	S	???	1	-0.960	1.740	-0.641
ATOM	114	C	???	1	0.051	2.311	-1.994
ATOM	115	C	???	1	1.332	1.839	-2.145
ATOM	116	S	???	1	1.979	0.583	-1.071
ATOM	117	C	???	1	2.142	2.402	-3.234
ATOM	118	N	???	1	3.411	2.685	-3.155
ATOM	119	C	???	1	4.021	3.320	-4.227
ATOM	120	C	???	1	5.384	3.786	-4.112
ATOM	121	O	???	1	6.097	3.752	-3.098
ATOM	122	N	???	1	5.908	4.352	-5.303
ATOM	123	H	???	1	6.893	4.688	-5.281
ATOM	124	C	???	1	5.213	4.429	-6.481
ATOM	125	N	???	1	5.869	4.830	-7.591
ATOM	126	H	???	1	6.848	5.183	-7.558
ATOM	127	H	???	1	5.332	5.058	-8.433
ATOM	128	N	???	1	3.918	4.094	-6.556
ATOM	129	C	???	1	3.346	3.588	-5.434
ATOM	130	N	???	1	2.015	3.260	-5.521
ATOM	131	H	???	1	1.530	3.465	-6.397
ATOM	132	C	???	1	1.250	2.791	-4.407
ATOM	133	H	???	1	0.612	1.944	-4.727
ATOM	134	O	???	1	0.368	3.845	-3.940
ATOM	135	C	???	1	-0.534	3.393	-2.910

```
ATOM    136  H   ???     1       -1.456   3.000  -3.399
ATOM    137  C   ???     1       -0.892   4.633  -2.105
ATOM    138  H   ???     1        0.023   5.032  -1.638
ATOM    139  H   ???     1       -1.626   4.381  -1.320
ATOM    140  O   ???     1       -1.442   5.652  -2.987
ATOM    141  P   ???     1       -1.409   7.177  -2.361
ATOM    142  O   ???     1       -1.085   8.094  -3.546
ATOM    143  O   ???     1       -0.421   7.310  -1.240
ATOM    144  O   ???     1       -2.927   7.434  -1.831
ATOM    145  H   ???     1       -2.947   7.658  -0.863
ATOM    146 MO   ???     1        0.180  -0.162   0.415
ATOM    147  O   ???     1       -0.563  -1.568  -0.296
ATOM    148  O   ???     1        1.715  -0.627   1.549
ATOM    149  O   ???     1        6.465  -2.358   1.402
ATOM    150  H   ???     1        6.264  -1.574   0.703
ATOM    151  H   ???     1        6.255  -3.267   1.067
ATOM    152  O   ???     1        6.040  -2.251   3.909
ATOM    153  H   ???     1        5.077  -2.133   4.099
ATOM    154  H   ???     1        6.107  -2.258   2.872
ATOM    155  O   ???     1        7.653  -0.271   4.660
ATOM    156  H   ???     1        7.100  -1.064   4.363
ATOM    157  H   ???     1        7.570  -0.305   5.666
ATOM    158  O   ???     1        9.144  -2.067   1.609
ATOM    159  H   ???     1        9.456  -2.998   1.481
ATOM    160  H   ???     1        8.147  -2.177   1.654
ATOM    161  O   ???     1        7.530  -4.530   3.762
ATOM    162  H   ???     1        6.978  -3.741   4.029
ATOM    163  H   ???     1        7.061  -4.826   2.935
END
MPO, state Im
ATOM      1  H   ???     1        2.390   3.217   1.500
ATOM      2  C   ???     1        3.333   2.951   0.974
ATOM      3  H   ???     1        3.130   2.740  -0.089
ATOM      4  H   ???     1        4.060   3.771   1.056
ATOM      5  N   ???     1        3.880   1.757   1.617
ATOM      6  H   ???     1        3.279   0.923   1.583
ATOM      7  C   ???     1        4.985   1.693   2.374
ATOM      8  N   ???     1        5.786   2.770   2.531
ATOM      9  H   ???     1        5.391   3.716   2.478
ATOM     10  H   ???     1        6.622   2.674   3.127
ATOM     11  N   ???     1        5.322   0.532   2.957
ATOM     12  H   ???     1        4.728  -0.299   2.850
ATOM     13  H   ???     1        6.126   0.471   3.600
ATOM     14  H   ???     1        8.175   2.419  -1.055
ATOM     15  C   ???     1        7.445   1.623  -0.846
ATOM     16  N   ???     1        6.156   1.597  -1.336
ATOM     17  H   ???     1        5.752   2.322  -1.962
ATOM     18  C   ???     1        5.561   0.463  -0.884
ATOM     19  H   ???     1        4.525   0.200  -1.107
ATOM     20  N   ???     1        6.401  -0.239  -0.119
ATOM     21  C   ???     1        7.582   0.476  -0.084
ATOM     22  H   ???     1        8.449   0.103   0.468
ATOM     23  H   ???     1       -3.291   0.196   1.474
ATOM     24  C   ???     1       -2.712   0.805   2.207
ATOM     25  H   ???     1       -3.254   0.837   3.170
ATOM     26  H   ???     1       -2.646   1.822   1.786
ATOM     27  S   ???     1       -1.027   0.125   2.501
ATOM     28  H   ???     1       -2.842  -2.704   1.487
ATOM     29  C   ???     1       -2.840  -3.751   1.904
ATOM     30  H   ???     1       -3.774  -4.238   1.578
ATOM     31  H   ???     1       -1.967  -4.286   1.480
ATOM     32  C   ???     1       -2.752  -3.794   3.443
ATOM     33  O   ???     1       -3.313  -4.722   4.047
ATOM     34  N   ???     1       -2.001  -2.836   4.005
ATOM     35  H   ???     1       -1.589  -2.096   3.412
ATOM     36  C   ???     1       -1.800  -2.680   5.441
ATOM     37  H   ???     1       -1.779  -3.683   5.908
ATOM     38  H   ???     1       -0.817  -2.195   5.558
ATOM     39  H   ???     1       -2.549  -2.074   5.972
ATOM     40  H   ???     1        0.487  -3.537  10.189
ATOM     41  C   ???     1        1.436  -3.379   9.620
ATOM     42  H   ???     1        1.704  -2.317   9.674
ATOM     43  H   ???     1        2.236  -3.948  10.129
ATOM     44  N   ???     1        1.361  -3.833   8.230
ATOM     45  H   ???     1        1.383  -4.850   8.080
ATOM     46  C   ???     1        1.356  -3.102   7.101
ATOM     47  N   ???     1        1.403  -1.767   7.072
ATOM     48  H   ???     1        1.249  -1.192   7.912
ATOM     49  H   ???     1        1.427  -1.273   6.156
```

ATOM	50	N	???	1	1.426	-3.779	5.915
ATOM	51	H	???	1	1.191	-4.775	5.958
ATOM	52	H	???	1	1.139	-3.299	5.026
ATOM	53	H	???	1	3.315	1.102	8.351
ATOM	54	C	???	1	2.963	1.682	7.460
ATOM	55	H	???	1	2.091	1.149	7.035
ATOM	56	H	???	1	2.595	2.669	7.805
ATOM	57	C	???	1	4.024	1.892	6.356
ATOM	58	H	???	1	4.410	0.910	5.998
ATOM	59	C	???	1	3.319	2.539	5.156
ATOM	60	H	???	1	2.729	3.417	5.478
ATOM	61	H	???	1	4.055	2.868	4.407
ATOM	62	H	???	1	2.627	1.822	4.678
ATOM	63	C	???	1	5.236	2.727	6.812
ATOM	64	H	???	1	5.806	2.226	7.621
ATOM	65	H	???	1	5.932	2.880	5.961
ATOM	66	H	???	1	4.911	3.718	7.182
ATOM	67	H	???	1	-2.062	1.511	8.433
ATOM	68	C	???	1	-1.385	1.728	7.596
ATOM	69	C	???	1	-0.796	0.782	6.779
ATOM	70	H	???	1	-0.798	-0.301	6.861
ATOM	71	N	???	1	-0.180	1.394	5.709
ATOM	72	H	???	1	0.282	0.890	4.943
ATOM	73	C	???	1	-0.375	2.755	5.795
ATOM	74	C	???	1	-0.016	3.781	4.908
ATOM	75	H	???	1	0.510	3.548	3.979
ATOM	76	C	???	1	-0.385	5.089	5.235
ATOM	77	H	???	1	-0.127	5.912	4.562
ATOM	78	C	???	1	-1.123	5.355	6.415
ATOM	79	H	???	1	-1.427	6.379	6.638
ATOM	80	C	???	1	-1.505	4.334	7.286
ATOM	81	H	???	1	-2.119	4.553	8.163
ATOM	82	C	???	1	-1.123	3.007	6.993
ATOM	83	H	???	1	1.854	-6.252	-1.687
ATOM	84	C	???	1	2.203	-5.269	-1.324
ATOM	85	C	???	1	1.645	-4.738	-0.150
ATOM	86	H	???	1	0.825	-5.261	0.363
ATOM	87	C	???	1	2.120	-3.551	0.410
ATOM	88	H	???	1	1.665	-3.167	1.327
ATOM	89	C	???	1	3.181	-2.862	-0.213
ATOM	90	O	???	1	3.689	-1.713	0.283
ATOM	91	H	???	1	3.063	-1.349	0.974
ATOM	92	C	???	1	3.744	-3.380	-1.396
ATOM	93	H	???	1	4.542	-2.810	-1.883
ATOM	94	C	???	1	3.259	-4.574	-1.941
ATOM	95	H	???	1	3.707	-4.951	-2.862
ATOM	96	H	???	1	5.092	-8.255	5.430
ATOM	97	C	???	1	5.602	-7.257	5.344
ATOM	98	H	???	1	5.526	-6.740	6.311
ATOM	99	H	???	1	6.674	-7.418	5.127
ATOM	100	N	???	1	5.031	-6.450	4.270
ATOM	101	H	???	1	5.241	-6.724	3.295
ATOM	102	C	???	1	4.383	-5.276	4.380
ATOM	103	N	???	1	4.189	-4.666	5.570
ATOM	104	H	???	1	4.420	-5.145	6.450
ATOM	105	H	???	1	3.355	-4.060	5.623
ATOM	106	N	???	1	3.886	-4.706	3.267
ATOM	107	H	???	1	4.128	-5.155	2.370
ATOM	108	H	???	1	3.616	-3.703	3.254
ATOM	109	S	???	1	1.930	-1.371	3.322
ATOM	110	O	???	1	0.994	-2.556	3.381
ATOM	111	O	???	1	1.680	-0.405	4.451
ATOM	112	O	???	1	3.385	-1.849	3.346
ATOM	113	S	???	1	-0.949	1.699	-0.602
ATOM	114	C	???	1	0.077	2.287	-1.956
ATOM	115	C	???	1	1.359	1.822	-2.090
ATOM	116	S	???	1	1.972	0.537	-0.995
ATOM	117	C	???	1	2.167	2.373	-3.187
ATOM	118	N	???	1	3.435	2.672	-3.119
ATOM	119	C	???	1	4.032	3.305	-4.201
ATOM	120	C	???	1	5.391	3.784	-4.095
ATOM	121	O	???	1	6.108	3.758	-3.083
ATOM	122	N	???	1	5.906	4.350	-5.288
ATOM	123	H	???	1	6.890	4.690	-5.272
ATOM	124	C	???	1	5.205	4.424	-6.463
ATOM	125	N	???	1	5.857	4.833	-7.573
ATOM	126	H	???	1	6.835	5.187	-7.543
ATOM	127	H	???	1	5.320	5.053	-8.418
ATOM	128	N	???	1	3.913	4.077	-6.534

```
ATOM    129  C   ???     1       3.352   3.558  -5.410
ATOM    130  N   ???     1       2.030   3.198  -5.493
ATOM    131  H   ???     1       1.535   3.405  -6.363
ATOM    132  C   ???     1       1.272   2.737  -4.369
ATOM    133  H   ???     1       0.643   1.879  -4.677
ATOM    134  O   ???     1       0.381   3.792  -3.927
ATOM    135  C   ???     1      -0.510   3.363  -2.877
ATOM    136  H   ???     1      -1.443   2.973  -3.346
ATOM    137  C   ???     1      -0.844   4.622  -2.089
ATOM    138  H   ???     1       0.083   5.024  -1.652
ATOM    139  H   ???     1      -1.564   4.390  -1.284
ATOM    140  O   ???     1      -1.405   5.631  -2.979
ATOM    141  P   ???     1      -1.406   7.158  -2.354
ATOM    142  O   ???     1      -1.106   8.083  -3.539
ATOM    143  O   ???     1      -0.414   7.317  -1.240
ATOM    144  O   ???     1      -2.928   7.372  -1.814
ATOM    145  H   ???     1      -2.945   7.619  -0.852
ATOM    146 MO   ???     1       0.138  -0.181   0.408
ATOM    147  O   ???     1      -0.616  -1.561  -0.329
ATOM    148  O   ???     1       1.802  -0.656   1.899
ATOM    149  O   ???     1       6.465  -2.360   1.405
ATOM    150  H   ???     1       6.265  -1.575   0.705
ATOM    151  H   ???     1       6.257  -3.269   1.070
ATOM    152  O   ???     1       6.070  -2.259   3.918
ATOM    153  H   ???     1       5.108  -2.145   4.116
ATOM    154  H   ???     1       6.126  -2.265   2.880
ATOM    155  O   ???     1       7.660  -0.266   4.663
ATOM    156  H   ???     1       7.113  -1.064   4.366
ATOM    157  H   ???     1       7.576  -0.300   5.669
ATOM    158  O   ???     1       9.146  -2.068   1.607
ATOM    159  H   ???     1       9.458  -2.999   1.481
ATOM    160  H   ???     1       8.150  -2.178   1.653
ATOM    161  O   ???     1       7.541  -4.544   3.765
ATOM    162  H   ???     1       6.993  -3.751   4.032
ATOM    163  H   ???     1       7.066  -4.841   2.942
END
MPO, state P
ATOM      1  H   ???     1       2.395   3.211   1.505
ATOM      2  C   ???     1       3.345   2.935   0.993
ATOM      3  H   ???     1       3.143   2.682  -0.062
ATOM      4  H   ???     1       4.054   3.773   1.037
ATOM      5  N   ???     1       3.923   1.785   1.684
ATOM      6  H   ???     1       3.336   0.947   1.787
ATOM      7  C   ???     1       5.061   1.764   2.402
ATOM      8  N   ???     1       5.852   2.852   2.498
ATOM      9  H   ???     1       5.432   3.789   2.457
ATOM     10  H   ???     1       6.694   2.774   3.088
ATOM     11  N   ???     1       5.430   0.618   2.988
ATOM     12  H   ???     1       4.787  -0.178   2.922
ATOM     13  H   ???     1       6.225   0.561   3.649
ATOM     14  H   ???     1       8.172   2.419  -1.057
ATOM     15  C   ???     1       7.439   1.624  -0.852
ATOM     16  N   ???     1       6.147   1.613  -1.338
ATOM     17  H   ???     1       5.748   2.340  -1.961
ATOM     18  C   ???     1       5.547   0.478  -0.892
ATOM     19  H   ???     1       4.509   0.222  -1.114
ATOM     20  N   ???     1       6.383  -0.237  -0.139
ATOM     21  C   ???     1       7.568   0.469  -0.101
ATOM     22  H   ???     1       8.433   0.087   0.448
ATOM     23  H   ???     1      -3.274   0.190   1.470
ATOM     24  C   ???     1      -2.662   0.785   2.188
ATOM     25  H   ???     1      -3.126   0.784   3.190
ATOM     26  H   ???     1      -2.604   1.814   1.796
ATOM     27  S   ???     1      -0.954   0.066   2.325
ATOM     28  H   ???     1      -2.842  -2.704   1.485
ATOM     29  C   ???     1      -2.840  -3.751   1.901
ATOM     30  H   ???     1      -3.773  -4.238   1.572
ATOM     31  H   ???     1      -1.967  -4.286   1.478
ATOM     32  C   ???     1      -2.753  -3.795   3.441
ATOM     33  O   ???     1      -3.320  -4.720   4.039
ATOM     34  N   ???     1      -1.994  -2.843   4.006
ATOM     35  H   ???     1      -1.599  -2.098   3.412
ATOM     36  C   ???     1      -1.799  -2.690   5.447
ATOM     37  H   ???     1      -1.794  -3.694   5.906
ATOM     38  H   ???     1      -0.806  -2.232   5.573
ATOM     39  H   ???     1      -2.548  -2.078   5.974
ATOM     40  H   ???     1       0.498  -3.540  10.231
ATOM     41  C   ???     1       1.478  -3.388   9.738
ATOM     42  H   ???     1       1.730  -2.320   9.757
```

ATOM	43	H	???	1	2.247	-3.907	10.341
ATOM	44	N	???	1	1.512	-3.918	8.390
ATOM	45	H	???	1	1.550	-4.937	8.282
ATOM	46	C	???	1	1.346	-3.237	7.237
ATOM	47	N	???	1	1.278	-1.904	7.199
ATOM	48	H	???	1	1.189	-1.332	8.047
ATOM	49	H	???	1	1.279	-1.406	6.285
ATOM	50	N	???	1	1.287	-3.962	6.105
ATOM	51	H	???	1	1.498	-4.960	6.181
ATOM	52	H	???	1	1.330	-3.527	5.161
ATOM	53	H	???	1	3.314	1.098	8.348
ATOM	54	C	???	1	2.959	1.671	7.454
ATOM	55	H	???	1	2.099	1.115	7.034
ATOM	56	H	???	1	2.577	2.656	7.790
ATOM	57	C	???	1	4.019	1.878	6.348
ATOM	58	H	???	1	4.409	0.897	6.002
ATOM	59	C	???	1	3.307	2.505	5.140
ATOM	60	H	???	1	2.704	3.378	5.449
ATOM	61	H	???	1	4.039	2.839	4.387
ATOM	62	H	???	1	2.627	1.773	4.666
ATOM	63	C	???	1	5.222	2.731	6.792
ATOM	64	H	???	1	5.795	2.245	7.607
ATOM	65	H	???	1	5.918	2.879	5.940
ATOM	66	H	???	1	4.890	3.725	7.150
ATOM	67	H	???	1	-2.063	1.511	8.434
ATOM	68	C	???	1	-1.388	1.727	7.597
ATOM	69	C	???	1	-0.790	0.775	6.794
ATOM	70	H	???	1	-0.773	-0.306	6.896
ATOM	71	N	???	1	-0.184	1.380	5.713
ATOM	72	H	???	1	0.342	0.866	4.998
ATOM	73	C	???	1	-0.380	2.742	5.789
ATOM	74	C	???	1	-0.018	3.764	4.898
ATOM	75	H	???	1	0.514	3.527	3.974
ATOM	76	C	???	1	-0.387	5.074	5.217
ATOM	77	H	???	1	-0.123	5.894	4.543
ATOM	78	C	???	1	-1.128	5.347	6.393
ATOM	79	H	???	1	-1.431	6.373	6.611
ATOM	80	C	???	1	-1.513	4.330	7.268
ATOM	81	H	???	1	-2.128	4.554	8.145
ATOM	82	C	???	1	-1.131	3.001	6.984
ATOM	83	H	???	1	1.849	-6.244	-1.681
ATOM	84	C	???	1	2.184	-5.251	-1.310
ATOM	85	C	???	1	1.652	-4.735	-0.114
ATOM	86	H	???	1	0.860	-5.279	0.420
ATOM	87	C	???	1	2.124	-3.542	0.447
ATOM	88	H	???	1	1.726	-3.174	1.401
ATOM	89	C	???	1	3.165	-2.838	-0.197
ATOM	90	O	???	1	3.691	-1.700	0.297
ATOM	91	H	???	1	3.336	-1.480	1.217
ATOM	92	C	???	1	3.685	-3.330	-1.413
ATOM	93	H	???	1	4.469	-2.750	-1.913
ATOM	94	C	???	1	3.204	-4.526	-1.956
ATOM	95	H	???	1	3.636	-4.890	-2.892
ATOM	96	H	???	1	5.090	-8.251	5.431
ATOM	97	C	???	1	5.591	-7.247	5.344
ATOM	98	H	???	1	5.517	-6.729	6.310
ATOM	99	H	???	1	6.662	-7.394	5.109
ATOM	100	N	???	1	4.971	-6.459	4.283
ATOM	101	H	???	1	5.114	-6.770	3.307
ATOM	102	C	???	1	4.410	-5.236	4.375
ATOM	103	N	???	1	4.194	-4.612	5.545
ATOM	104	H	???	1	4.369	-5.046	6.459
ATOM	105	H	???	1	3.888	-3.627	5.499
ATOM	106	N	???	1	4.031	-4.623	3.240
ATOM	107	H	???	1	4.239	-5.108	2.355
ATOM	108	H	???	1	3.275	-3.910	3.270
ATOM	109	S	???	1	2.456	-1.498	3.924
ATOM	110	O	???	1	1.837	-2.855	3.560
ATOM	111	O	???	1	1.469	-0.662	4.694
ATOM	112	O	???	1	3.690	-1.766	4.798
ATOM	113	S	???	1	-1.006	1.739	-0.725
ATOM	114	C	???	1	0.037	2.347	-2.060
ATOM	115	C	???	1	1.317	1.884	-2.172
ATOM	116	S	???	1	1.887	0.547	-1.106
ATOM	117	C	???	1	2.140	2.429	-3.256
ATOM	118	N	???	1	3.411	2.706	-3.172
ATOM	119	C	???	1	4.026	3.335	-4.241
ATOM	120	C	???	1	5.392	3.799	-4.124
ATOM	121	O	???	1	6.098	3.772	-3.107

```
ATOM    122  N   ???     1       5.920   4.356  -5.317
ATOM    123  H   ???     1       6.904   4.695  -5.293
ATOM    124  C   ???     1       5.228   4.431  -6.496
ATOM    125  N   ???     1       5.881   4.829  -7.606
ATOM    126  H   ???     1       6.860   5.182  -7.576
ATOM    127  H   ???     1       5.343   5.054  -8.450
ATOM    128  N   ???     1       3.933   4.096  -6.573
ATOM    129  C   ???     1       3.356   3.598  -5.454
ATOM    130  N   ???     1       2.026   3.273  -5.547
ATOM    131  H   ???     1       1.546   3.481  -6.425
ATOM    132  C   ???     1       1.254   2.821  -4.435
ATOM    133  H   ???     1       0.605   1.981  -4.749
ATOM    134  O   ???     1       0.380   3.890  -3.976
ATOM    135  C   ???     1      -0.542   3.425  -2.971
ATOM    136  H   ???     1      -1.449   3.027  -3.483
ATOM    137  C   ???     1      -0.930   4.641  -2.146
ATOM    138  H   ???     1      -0.032   5.035  -1.643
ATOM    139  H   ???     1      -1.680   4.359  -1.385
ATOM    140  O   ???     1      -1.473   5.671  -3.010
ATOM    141  P   ???     1      -1.412   7.192  -2.370
ATOM    142  O   ???     1      -1.066   8.105  -3.551
ATOM    143  O   ???     1      -0.430   7.287  -1.241
ATOM    144  O   ???     1      -2.927   7.481  -1.850
ATOM    145  H   ???     1      -2.952   7.685  -0.877
ATOM    146 MO   ???     1      -0.012  -0.188   0.131
ATOM    147  O   ???     1      -0.856  -1.496  -0.641
ATOM    148  O   ???     1       2.863  -0.784   2.621
ATOM    149  O   ???     1       6.421  -2.375   1.401
ATOM    150  H   ???     1       6.231  -1.616   0.693
ATOM    151  H   ???     1       6.248  -3.298   1.083
ATOM    152  O   ???     1       6.191  -2.260   3.951
ATOM    153  H   ???     1       5.253  -2.120   4.278
ATOM    154  H   ???     1       6.152  -2.271   2.926
ATOM    155  O   ???     1       7.688  -0.241   4.671
ATOM    156  H   ???     1       7.165  -1.059   4.361
ATOM    157  H   ???     1       7.591  -0.284   5.676
ATOM    158  O   ???     1       9.117  -2.076   1.608
ATOM    159  H   ???     1       9.414  -3.014   1.497
ATOM    160  H   ???     1       8.120  -2.171   1.659
ATOM    161  O   ???     1       7.577  -4.569   3.784
ATOM    162  H   ???     1       7.056  -3.748   4.032
ATOM    163  H   ???     1       7.076  -4.882   2.985
END
```
